# Taxonomic review of the *Ornithocheirus* complex (Pterosauria) from the Cretaceous of England

**DOI:** 10.3897/zookeys.308.5559

**Published:** 2013-06-12

**Authors:** Taissa Rodrigues, Alexander Wilhelm Armin Kellner

**Affiliations:** 1 Department of Biology, Agrarian Sciences Center, Universidade Federal do Espírito Santo. Alto Universitário s/n, Caixa Postal 16, Guararema, CEP 29500–000, Alegre, ES, Brazil; 2 Laboratory of Systematics and Taphonomy of Fossil Vertebrates, Department of Geology and Paleontology, Museu Nacional / Universidade Federal do Rio de Janeiro. Quinta da Boa Vista s/n, São Cristóvão, CEP 20940–040, Rio de Janeiro, RJ, Brazil

**Keywords:** Pterodactyloidea, Ornithocheiridae, Anhangueridae, Lonchodraconidae, Anhangueria, Cretaceous, Cambridge Greensand

## Abstract

Over a decade after the last major review of the Cambridge Greensand pterosaurs, their systematics remains one of the most disputed points in pterosaur taxonomy. Ornithocheiridae is still a wastebasket for fragmentary taxa, and some nomenclatural issues are still a problem. Here, the species from the Cretaceous of England that, at some point, were referred in *Ornithocheirus*, are reviewed. Investigation of the primary literature confirmed that *Criorhynchus* should be considered an objective junior synonym of *Ornithocheirus*. Taxonomic review of more than 30 species known from fragmentary remains showed that 16 of them are undiagnosable (*nomina dubia*): *Palaeornis cliftii*, *Cimoliornis diomedeus*, *Pterodactylus compressirostris*, *Pterodactylus fittoni*, *Pterodactylus woodwardi*, *Ornithocheirus brachyrhinus*, *Ornithocheirus carteri*, *Ornithocheirus crassidens*, *Ornithocheirus dentatus*, *Ornithocheirus enchorhynchus*, *Ornithocheirus eurygnathus*, *Ornithocheirus oxyrhinus*, *Ornithocheirus scaphorhynchus*, *Ornithocheirus tenuirostris*, *Ornithocheirus xyphorhynchus*, and *Pterodactylus sagittirostris*. Fourteen species are considered valid, and diagnoses are provided to all of them: *Ornithocheirus simus*, *Lonchodraco giganteus* **comb. n.**, *Lonchodraco machaerorhynchus* **comb. n.**, *Lonchodraco(?) microdon* **comb. n.**, *Coloborhynchus clavirostris*, *‘Ornithocheirus’ capito*, *Camposipterus nasutus* **comb. n.**, *Camposipterus(?) sedgwickii* **comb. n.**, *Camposipterus(?) colorhinus* **comb. n.**, *Cimoliopterus cuvieri* **comb. n.**, *‘Ornithocheirus’ polyodon*, *‘Ornithocheirus’ platystomus*, *‘Pterodactylus’ daviesii*, and *‘Ornithocheirus’ denticulatus*. These species are referred in the genera *Ornithocheirus*, *Lonchodraco* **gen. n.**, *Coloborhynchus*, *Cimoliopterus* **gen. n.**, and *Camposipterus* **gen. n.**, but additional genera are probably present, as indicated by the use of single quotation marks throughout the text. A cladistic analysis demonstrates that Anhangueridae lies within a newly recognized clade, here named Anhangueria, which also includes the genera *Cearadactylus*, *Brasileodactylus*, *Ludodactylus*, and *Camposipterus*. The anhanguerian *‘Cearadactylus’ ligabuei* belongs to a different genus than *Cearadactylus atrox*. Lonchodraconidae **fam. n.** (more or less equivalent to Lonchodectidae *sensu* Unwin 2001) is a monophyletic entity, but its exact phylogenetic position remains uncertain, as is the case of *Ornithocheirus simus*. Therefore, it is proposed that Ornithocheiridae should be constricted to its type species and thus is redundant. Other taxa previously referred as “ornithocheirids” are discussed in light of the revised taxonomy.

## Introduction

The Cretaceous of England is exceptionally rich in pterosaur fossils, which are of historical, morphological, and taxonomic importance. Several deposits contain pterosaur remains, among them the Hastings Group (late Berriasian / Valanginian), Wessex Formation (Barremian), Vectis Formation (Barremian / early Aptian), Gault Clay Formation (Albian), Cambridge Greensand (Cenomanian deposit with reworked fossils thought to be Albian in age) and Chalk Formation (Cenomanian / Turonian) (Rawson et al. 1978; Unwin et al. 2000; Unwin 2001; Barrett et al. 2008; Witton et al. 2009; Naish et al. 2013). The Cambridge Greensand in itself is one of the richest pterosaur deposits in the world (Wellnhofer 1991a; Kellner 1994), with over 2000 known specimens. Unlike that from most other pterosaur–bearing deposits, the Cambridge Greensand material is not flattened; it is, however, quite fragmentary and found isolated (Hooley 1914; Wellnhofer 1978; Kellner 1990; Wellnhofer 1991a; Unwin 2001). The nature of these fossils, along with decades of competing taxonomic proposals, synonymisations, and misunderstandings, as detailed below, made their taxonomy controversial (see Unwin 2001 and Table 1).

**Table 1.** Abbreviated taxonomic history of the *Ornithocheirus* complex from the Cretaceous of England. Single quotation marks indicate provisional genera.<br/>

**Original description**	**Seeley, 1870**	**Hooley, 1914**	**Wellnhofer, 1978**	**Unwin, 2001**	**This work**
*Ornithocheirus brachyrhinus* Seeley, 1870	*Ornithocheirus brachyrhinus*	*Ornithocheirus brachyrhinus*	*Ornithocheirus brachyrhinus*	*Anhanguera cuvieri*	*nomen dubium*
*Ornithocheirus capito* Seeley, 1870	*Ornithocheirus capito*	*Criorhynchus capito*	*Criorhynchus capito*	*Coloborhynchus capito*	*‘Ornithocheirus’ capito*
*Ornithocheirus carteri* Seeley, 1870	*Ornithocheirus carteri*	–	*Criorhynchus simus*	*Ornithocheirus simus*	*nomen dubium*
*Coloborhynchus clavirostris* Owen, 1874	–	*Criorhynchus simus*	*Criorhynchus simus*	*Coloborhynchus clavirostris*	*Coloborhynchus clavirostris*
*Ornithocheirus colorhinus* Seeley, 1870	*Ornithocheirus colorhinus*	*Ornithocheirus colorhinus*	*Ornithocheirus colorhinus*	*Anhanguera cuvieri*	*Camposipterus(?) colorhinus*
*Pterodactylus compressirostris* Owen, 1852	–	*Lonchodectes compressirostris*	*Ornithocheirus compressirostris*	*Lonchodectes compressirostris*	*nomen dubium*
*Pterodactylus cuvieri* Bowerbank, 1852	*Ornithocheirus cuvieri*	*Ornithocheirus cuvieri*	*Ornithocheirus cuvieri*	*Anhanguera cuvieri*	*Cimoliopterus cuvieri*
*Palaeornis cliftii* Mantell, 1844	–	*Ornithocheirus clifti [sic]*	*Ornithocheirus clifti [sic]*	–	*nomen dubium*
*Ornithocheirus crassidens* Seeley, 1870	*Ornithocheirus crassidens*	*Amblydectes crassidens*	*Criorhynchus crassidens*	*Coloborhynchus sedgwickii*	*nomen dubium*
*Coloborhynchus clavirostris* Owen, 1874	–	*Criorhynchus simus*	*Criorhynchus simus*	*Coloborhynchus clavirostris*	*Coloborhynchus clavirostris*
*Pterodactylus curtus* Owen, 1874	–	*Ornithocheirus curtus*	*Ornithocheirus curtus*	–	*nomen nudum*
*Pterodactylus daviesii* Owen, 1874	–	*Lonchodectes daviesii*	*Ornithocheirus daviesi [sic]*	*Lonchodectes platystomus*	*‘Pterodactylus’ daviesii*
*Ornithocheirus dentatus* Seeley, 1870	*Ornithocheirus dentatus*	*Ornithocheirus dentatus*	*Ornithocheirus dentatus*	*Anhanguera cuvieri*	*nomen dubium*
*Ornithocheirus denticulatus* Seeley, 1870	*Ornithocheirus denticulatus*	*Ornithocheirus denticulatus*	–	*Anhanguera cuvieri*	*‘Ornithocheirus’ denticulatus*
*Cimoliornis diomedeus* Owen, 1846	–	*Ornithocheirus diomedius [sic]*	*Ornithocheirus diomedius [sic]*	–	*nomen dubium*
*Ornithocheirus enchorhynchus* Seeley, 1870	*Ornithocheirus enchorhynchus*	*Ornithocheirus enchorhynchus*	*Ornithocheirus enchorhynchus*	*Anhanguera cuvieri*	*nomen dubium*
*Ornithocheirus eurygnathus* Seeley, 1870	*Ornithocheirus eurygnathus*	*Amblydectes eurygnathus*	*Criorhynchus eurygnathus*	*Coloborhynchus capito*	*nomen dubium*
*Pterodactylus fittoni* Owen, 1859	*Ornithocheirus fittoni*	*Ornithocheirus fittoni*	*Ornithocheirus fittoni*	*Anhanguera fittoni*	*nomen dubium*
*Pterodactylus giganteus* Bowerbank, 1846	–	*Lonchodectes giganteus*	*Ornithocheirus giganteus*	*Lonchodectes giganteus*	*Lonchodraco giganteus*
*Ornithocheirus huxleyi* Seeley, 1870	*Ornithocheirus huxleyi*	–	*Ornithocheirus huxleyi*	*Lonchodectes microdon*	–
*Ornithocheirus machaerorhynchus* Seeley, 1870	*Ornithocheirus machaerorhynchus*	*Lonchodectes machaeorhynchus [sic]*	*Ornithocheirus machaeorhynchus [sic]*	*Lonchodectes machaerorhynchus*	*Lonchodraco machaerorhynchus*
*Ornithocheirus microdon* Seeley, 1870	*Ornithocheirus microdon*	*Lonchodectes microdon*	*Ornithocheirus microdon*	*Lonchodectes microdon*	*Lonchodraco(?) microdon*
*Ornithocheirus nasutus* Seeley, 1870	*Ornithocheirus nasutus*	*Ornithocheirus nasutus*	*Ornithocheirus nasutus*	*Anhanguera fittoni*	*Camposipterus nasutus*
*Pterodactylus nobilis*	–	*Ornithocheirus nobilis*	–	–	*nomen nudum*
*Ornithocheirus oweni* Seeley, 1870	*Ornithocheirus oweni*	*Lonchodectes oweni*	*Ornithocheirus oweni*	*Lonchodectes microdon*	*Lonchodraco(?) microdon*
*Ornithocheirus oxyrhinus* Seeley, 1870	*Ornithocheirus oxyrhinus*	*Ornithocheirus oxyrhinus*	*Ornithocheirus oxyrhinus*	*nomen nudum*	*nomen dubium*
*Ornithocheirus platyrhinus* Seeley, 1870	*Ornithocheirus platyrhinus*	*Criorhynchus platyrhinus*	*Criorhynchus simus*	*Ornithocheirus simus*	*Ornithocheirus simus*
*Ornithocheirus platystomus* Seeley, 1870	*Ornithocheirus platystomus*	*Amblydectes platystomus*	*Criorhynchus platystomus*	*Lonchodectes platystomus*	*‘Ornithocheirus’ platystomus*
*Ornithocheirus polyodon* Seeley, 1870	*Ornithocheirus polyodon*	*Ornithocheirus polyodon*	*Ornithocheirus polyodon*	*Anhanguera fittoni*	*‘Ornithocheirus’ polyodon*
*Ornithocheirus reedi* Seeley, 1870	*Ornithocheirus reedi*	*Criorhynchus reedi*	*Criorhynchus reedi*	*Coloborhynchus capito*	*‘Ornithocheirus’ capito*
*Pterodactylus sagittirostris* Owen, 1874	–	*Lonchodectes sagittirostris*	*Ornithocheirus sagittirostris*	*Lonchodectes sagittirostris*	*nomen dubium*
*Ornithocheirus scaphorhynchus* Seeley, 1870	*Ornithocheirus scaphorhynchus*	*Lonchodectes scaphorhynchus*	*Ornithocheirus scaphorhynchus*	*Anhanguera cuvieri*	*nomen dubium*
*Pterodactylus sedgwickii* Owen, 1859	*Ornithocheirus sedgwicki [sic]*	*Ornithocheirus sedgwicki [sic]*	*Ornithocheirus sedgwicki [sic]*	*Coloborhynchus sedgwickii*	*Camposipterus(?) sedgwickii*
*Pterodactylus simus* Owen, 1861	*Ornithocheirus simus*	*Criorhynchus simus*	*Criorhynchus simus*	*Ornithocheirus simus*	*Ornithocheirus simus*
*Ornithocheirus tenuirostris* Seeley, 1870	*Ornithocheirus tenuirostris*	*Lonchodectes tenuirostris*	*Ornithocheirus tenuirostris*	*Lonchodectes compressirostris*	*nomen dubium*
*Pterodactylus woodwardi* Owen, 1861	*Ornithocheirus woodwardi*	*Criorhynchus woodwardi*	*Criorhynchus simus*	*Coloborhynchus sedgwicki*	*nomen dubium*
*Ornithocheirus xyphorhynchus* Seeley, 1870	*Ornithocheirus xyphorhynchus*	*Ornithocheirus xyphorhynchus*	*Ornithocheirus xyphorhynchus*	*Anhanguera cuvieri*	*nomen dubium*

Pioneer works on this fauna, such as by James Scott Bowerbank (1797–1877) and Richard Owen (1804–1892), initially attributed the pterosaurs from the Cretaceous of England in the genus *Pterodactylus*, nowadays considered restricted to the Jurassic Solnhofen Limestone of Germany. Harry Govier Seeley (1839–1909) was the first researcher to separate the British forms in new genera.

In 1869, Seeley published an index of specimens from the collection of the Woodwardian Museum (now Sedgwick Museum of Earth Sciences) of the University of Cambridge. This index presented 24 named pterosaur species from the Cretaceous of England, divided in two genera, *“Ptenodactylus”* and *Ornithocheirus*. At first glance, this work presents nomenclatural problems as all new species lacked descriptions and would, in principle, be considered *nomina nuda* based on article 12.1 of the International Code of Zoological Nomenclature (ICZN) (International Commission On Zoological Nomenclature 1999). What apparently has gone unnoticed is that the nomenclatural acts concerning the naming of new species in Seeley’s 1869 work were disclaimed and therefore intentionally not available. In the first paragraph of page xv, it can be read in the definition of the work: “An approximate list of the species included in the following Catalogue, **with provisional names for new species** and reference to the specimens on which they are founded, and to the pages of the Index in which they are described. ” [emphasis added] and also a footnote also on page xv explaining the term “provisional names”: “These names are only intended for the convenience of students using the Museum, **and not necessarily to take rank as names of described species**” [emphasis added]. Disclaimed acts are recognized in ICZN’s article 8.3, with the result that the names then given for new species were not available.

It is also possible to interpret these sentences (Seeley 1869: xv) as stating that the new names for genera were not disclaimed. Concerning *Ornithocheirus*, it had a description that, albeit inadequate (“This genus has no teeth anterior to the palate.”), can be regarded as an action that makes the name available. Three species were originally referred to the genus: *Ornithocheirus simus*, “*Ornithocheirus carteri*” and “*Ornithocheirus platyrhinus*”. Since the last two were not available (as explained above), the only available species left on the original description, *Ornithocheirus simus*, is thus the type species of the genus *Ornithocheirus* by monotypy (ICZN articles 67.2, 67.2.1 and 68.3) (Unwin and Bakhurina 2000; Unwin 2001). *“Ptenodactylus”* Seeley, 1869, with 21 referred ‘species’, was not only preoccupied by *Ptenodactylus* Gray, 1845 (see Unwin 2001), but it was not associated with a description and thus can be considered a *nomen nudum*.

Seeley (1870) placed 27 species from the Cretaceous of England in the genus *Ornithocheirus* (Table 2). Although recognizing that *Ornithocheirus simus*, *Ornithocheirus carteri* and *Ornithocheirus platyrhinus* are known by much more massive jaws than the lanceolate tips of the other species, Seeley (1870) mistakenly reinterpreted the holotype of *Ornithocheirus simus* as a mandible, and thus his 1869 diagnosis of *Ornithocheirus*, based on the absence of anteriorly directed teeth in the premaxilla, would be invalidated. So he referred all these species to the same genus and re–diagnosed it as “in which teeth are prolonged anterior to the muzzle, and the palate has a longitudinal ridge”. The lumping of all these species into one single genus was the beginning of what can be referred as the *Ornithocheirus* complex: a wastebasket genus for species of uncertain relationships and represented by fragmentary type material (see Kellner 1994).

**Table 2.** List of taxa of the *Ornithocheirus* complex from the Cretaceous of England, after Seeley (1870).<br/>

Ornithocheiridae
*Ornithocheirus brachyrhinus*
*Ornithocheirus capito*
*Ornithocheirus carteri*
*Ornithocheirus colorhinus*
*Ornithocheirus crassidens*
*Ornithocheirus cuvieri*
*Ornithocheirus dentatus*
*Ornithocheirus denticulatus*
*Ornithocheirus enchorhynchus*
*Ornithocheirus eurygnathus*
*Ornithocheirus fittoni*
*Ornithocheirus huxleyi*
*Ornithocheirus machaerorhynchus*
*Ornithocheirus microdon*
*Ornithocheirus nasutus*
*Ornithocheirus oweni*
*Ornithocheirus oxyrhinus*
*Ornithocheirus platystomus*
*Ornithocheirus platyrhinus*
*Ornithocheirus polyodon*
*Ornithocheirus reedi*
*Ornithocheirus scaphorhynchus*
*Ornithocheirus sedgwicki*
*Ornithocheirus simus*
*Ornithocheirus tenuirostris*
*Ornithocheirus woodwardi*
*Ornithocheirus xyphorhynchus*

In 1874, Owen proposed two new genera for the Cretaceous British pterosaurs, *Criorhynchus* and *Coloborhynchus*. *Criorhynchus* was created as a monotypic genus including only *Criorhynchus simus* because Owen (1874) considered the name *Ornithocheirus* inappropriate (*ornitho*, bird, and *cheirus*, hand). At the time, the British Association Code allowed such emendations on ‘inappropriate’ names (Dayrat 2010), but the present ICZN has modified that. As both *Ornithocheirus* Seeley, 1869 and *Criorhynchus* Owen, 1874 have the same type species, they are objective synonyms, and the former has priority over the later (Principle of Priority, ICZN articles 23.1 and 61.3.3). *Coloborhynchus* had three species referred to it, and no type species was designated. Many years later, *Coloborhynchus clavirostris* was subsequently designated as type species by Kuhn (1967).

Lydekker (1888), following the suggestions by Seeley (1870), provided a catalogue in which then other named genera from the Cretaceous of England were synonymized with *Ornithocheirus* (with the incorrect spelling *Ornithochirus*) (Table 3). In addition, he included 14 species in *Ornithocheirus*, as “family uncertain”. He considered that “all the species are known by such fragmentary remains that no accurate diagnosis can be given” (Lydekker 1888: 10) and also that “many of the species are probably invalid” (Lydekker 1888: 11).

**Table 3.** List of taxa of the *Ornithocheirus* complex from the Cretaceous of England, after Lydekker (1888). = indicate synonymies.<br/>

Family uncertain
*Ornithochirus [sic] compressirostris*
*Ornithochirus [sic](?) clifti [sic]*
*Ornithochirus [sic](?) curtus*
*Ornithochirus [sic] cuvieri*
*Ornithochirus [sic] daviesi [sic]*
*Ornithochirus [sic] diomedius [sic]*
*Ornithochirus [sic] fittoni*
*Ornithochirus [sic](?) giganteus*
*Ornithochirus [sic] hlavatschi*
*Ornithochirus [sic] nobilis*
*Ornithochirus [sic] sedgwicki [sic]*
*Ornithochirus [sic](?) simus*
=(?) *Pterodactylus woodwardi*
*Ornithochirus [sic](?)* sp.
*Ornithochirus [sic] validus*
= ? *Pterodactylus macrurus*
= *Doratorhynchus validus*

Newton (1888) agreed with Lydekker (1888) that these species, based on fragmentary remains, should be included in a single genus, *Ornithocheirus*. However, he claimed the existence of 40 species, a number much higher than proposed by both Seeley (1870) and Lydekker (1888) but, unfortunately, did not list these species or provide information on how he achieved this number. Newton (1888) pointed out that the discovery of new, more complete specimens would probably at the same time reduce the number of species but increase the number of genera.

Woods (1891) provided a catalogue of the type fossils in the Woodwardian Museum in Cambridge and listed 25 species in the genus *Ornithocheirus*, similar to Seeley (1870), Lydekker (1888), and Newton (1888) (Table 4).

The first major review of the *Ornithocheirus* complex was provided by Hooley (1914) (Table 5). He provided an appraisal of previous reviews of the genus, reviewed the species present in the collection of the Sedgwick Museum and in other museums, and divided them in five groups or genera based on morphological characters. His group n. 1 had 16 species and was named *Ornithocheirus*; group n. 2, with nine species, *Lonchodectes*; group n. 3 held three species and was named *Amblydectes*; and group n. 4, with six species, was termed *Criorhynchus* (including *Coloborhynchus clavirostris* as a synonym of *Criorhynchus simus*). He added a fifth group, restricted to the edentulous form *Ornithostoma*, which was not part of the *Ornithocheirus* complex used by him.

**Table 4.** List of taxa of the *Ornithocheirus* complex from the Cretaceous of England, after Woods (1891). = indicate synonymies.<br/>

*Doratohynchus validum [sic]*
=*Pterodactylus macrurus*
*Ornithocheirus brachyrhinus*
*Ornithocheirus capito*
*Ornithocheirus carteri*
*Ornithocheirus colorhinus*
*Ornithocheirus compressirostris*
*Ornithocheirus crassidens*
*Ornithocheirus cuvieri*
*Ornithocheirus dentatus*
*Ornithocheirus denticulatus*
*Ornithocheirus enchorhynchus*
*Ornithocheirus eurygnathus*
*Ornithocheirus fittoni*
*Ornithocheirus machaerorhynchus*
*Ornithocheirus microdon*
*Ornithocheirus nasutus*
*Ornithocheirus oweni*
*Ornithocheirus oxyrhinus*
*Ornithocheirus platyrhinus*
*Ornithocheirus platysomus [sic]*
*Ornithocheirus polyodon*
*Ornithocheirus scaphorhynchus*
*Ornithocheirus sedgwicki [sic]*
*Ornithocheirus simus*
=*Pterodactylus woodwardi*
*Ornithocheirus tenuirostris*
*Pterodactylus hopkinsi*
*Pterodactylus oweni*

**Table 5.** List of taxa of the *Ornithocheirus* complex from the Cretaceous of England, after Hooley (1914). = indicate synonymies.<br/>

Ornithocheiridae
Ornithocheirinae
*Ornithocheirus brachyrhinus*
*Ornithocheirus clifti [sic]*
*Ornithocheirus colorhinus*
*Ornithocheirus curtus*
*Ornithocheirus cuvieri*
*Ornithocheirus dentatus*
*Ornithocheirus denticulatus*
*Ornithocheirus diomedius [sic]*
*Ornithocheirus enchorhynchus*
*Ornithocheirus fittoni*
*Ornithocheirus nasutus*
*Ornithocheirus nobilis*
*Ornithocheirus oxyrhinus*
*Ornithocheirus polyodon*
*Ornithocheirus sedgwicki [sic]*
*Ornithocheirus xyphorhynchus*
*Lonchodectes compressirostris*
*Lonchodectes daviesii*
*Lonchodectes giganteus*
*Lonchodectes machaeorhynchus [sic]*
*Lonchodectes microdon*
*Lonchodectes oweni*
*Lonchodectes sagittirostris*
*Lonchodectes scaphorhynchus*
*Lonchodectes tenuirostris*
Criorhynchinae
*Amblydectes crassidens*
*Amblydectes eurygnathus*
*Amblydectes platysomus [sic]*
*Criorhynchus capito*
*Criorhynchus carteri*
*Criorhynchus platyrhinus*
*Criorhynchus reedi*
*Criorhynchus simus*
=*Coloborhynchus clavirostris*
*Criorhynchus woodwardi*

*Criorhynchus* is a taxonomic problem by itself. Seeley (1869) was the first to recognize that *Ornithocheirus simus* was quite distinct from most other pterosaurs from this complex and introduced the genus *Ornithocheirus* for its reception, separating this species from the ones known by lanceolate jaws. As noted above, Owen (1874) accepted the distinction but regarded the name inappropriate, and thus assigned *Ornithocheirus simus* to a different genus (*Criorhynchus*). Lydekker (1888: 16, footnote) acknowledged that *Ornithocheirus simus* was the type species of the genus *Ornithocheirus* and both Lydekker (1888) and Hooley (1914) agreed that *Ornithocheirus simus* was distinguished by its tall rostrum, whereas most other species then referred in *Ornithocheirus* had lanceolate jaw tips. In order to avoid confusion, Lydekker (1888) preferred to use *Criorhynchus* for *Ornithocheirus simus* and *Ornithocheirus* for the species with lanceolate tips. Hooley (1914) was of similar opinion and favored the name *Criorhynchus* for *Ornithocheirus simus* and other species with tall rostra, and *Ornithocheirus* for the other taxa.

Subsequent authors tended to divide the species of the *Ornithocheirus* complex in only two genera, *Ornithocheirus* and *Criorhynchus* (e.g., Kuhn 1967; Wellnhofer 1978; Kellner and Tomida 2000), excluding *Pterodactylus simus* from *Ornithocheirus* and referring it to *Criorhynchus*, following Lydekker (1888). One major consequence was the uncertainty regarding a type species for *Ornithocheirus*. Khozatskii and Yur’ev (1964) referred *Pterodactylus compressirostris* as type species of *Ornithocheirus* and *Pterodactylus simus*, of *Criorhynchus*, both in the family Ornithocheiridae. They also considered *Amblydectes* and *Lonchodectes* as Pterosauria *incertae sedis*.

Kuhn (1967) recognized the genus *Criorhynchus*, with *Criorhynchus simus* as type species, in the family Criorhynchidae, with seven species which, according to him, were almost all indefinable. He also recognized the genus *Ornthocheirus [sic]*, with approximately 25 described speciesand *Pterodactylus compressirostris* as its type species, in the family Ornithocheiridae and subfamily Ornithocheirinae. He was the first author to define type species for the genera *Amblydectes* and *Lonchodectes*, respectively *Amblydectes crassidens* and *Lonchodectes compressirostris* (Kuhn 1967: 46, using the term “Genotypus”), but considered both genera indeterminate and deemed as synonyms of *Ornithocheirus*.

Wellnhofer (1978) published a major reference work on pterosaurs. He discussed the species included in *Ornithocheirus* based on the diagnoses provided by Seeley (1869, 1870), and drew attention to which was the first species cited in the lists provided by this author, probably trying to elucidate Seeley’s original intentions. He did not recognize *Pterodactylus simus* as the type species of *Ornithocheirus* and referred it as the type and only valid species of *Criorhynchus*, in the family Criorhynchidae. Four species were considered synonymous with *Criorhynchus simus*, and others were referred as Criorhynchidae *incertae sedis*. All other species of the *Ornithocheirus* complex were referred to the family Ornithocheiridae. Following Kuhn (1967), he incorrectly referred *Pterodactylus compressirostris* as the type species of *Ornithocheirus* and, from the *Ornithocheirus* complex, attributed eight species to the genus *Ornithocheirus*, four to Ornithocheiridae *incertae sedis*, and considered 17 of uncertain systematic position, among them four non–British species (Table 6).

**Table 6.** List of taxa of the *Ornithocheirus* complex from the Cretaceous of England, after Wellnhofer (1978). = indicate synonymies.<br/>

Ornithocheiridae
*Ornithocheirus compressirostris*
*Ornithocheirus cuvieri*
*Ornithocheirus daviesi [sic]*
*Ornithocheirus fittoni*
*Ornithocheirus giganteus*
*Ornithocheirus microdon*
*Ornithocheirus sagittirostris*
*Ornithocheirus sedgwicki [sic]*
Ornithocheiridae *incertae sedis*
*Ornithocheirus clifti [sic]*
*Ornithocheirus curtus*
*Ornithocheirus diomedius [sic]*
*Ornithocheirus validus*
Uncertain systematic position
*Ornithocheirus brachyrhinus*
*Ornithocheirus colorhinus*
*Ornithocheirus dentatus*
*Ornithocheirus enchorhynchus*
*Ornithocheirus huxleyi*
*Ornithocheirus nasutus*
*Ornithocheirus oxyrhinus*
*Ornithocheirus polyodon*
*Ornithocheirus machaeorhynchus [sic]*
*Ornithocheirus oweni*
*Ornithocheirus scaphorhynchus*
*Ornithocheirus tenuirostris*
*Ornithocheirus xyphorhynchus*
*“Ornithocheirus” bunzeli*
*“Ornithocheirus” hilsensis*
*“Ornithocheirus” hlavatschi*
cf. *Ornithocheirus*
Criorhynchidae
*Criorhynchus simus*
=*Coloborhynchus clavirostris*
= ? *Criorhynchus woodwardi*
= ? *Criorhynchus carteri*
= ? *Criorhynchus platyrhinus*
Criorhynchidae *incertae sedis*
*Criorhynchus eurygnathus*
*Criorhynchus capito*
*Criorhynchus crassidens*
*Criorhynchus platystomus*
*Criorhynchus reedi*

Unwin (2001) undertook the most recent review. He revised the taxonomic history of the pterosaurs from the Cambridge Greensand and, although more complete specimens of the *Ornithocheirus* complex from the Cretaceous of England had not been discovered, he compared them with the more complete and more recently described pterosaurs from the Santana Group of Brazil. Unwin (2001) designated *Pterodactylus simus* as the type species of *Ornithocheirus* and divided the Cambridge Greensand species in two families and four genera: Ornithocheiridae with a monospecific *Ornithocheirus*, *Coloborhynchus* with two species, *Anhanguera* with two species, and Lonchodectidae comprising six species of the genus *Lonchodectes* (Table 7). Several named species were synonymized with others but diagnoses for the species considered valid were not presented and thus it is not clear which characters were used for these referrals.

**Table 7.** List of taxa of the *Ornithocheirus* complex from the Cretaceous of England, after Unwin (2001). = indicate synonymies.<br/>

Ornithocheiridae
*Ornithocheirus simus*
=*Ornithocheirus carteri*
=*Ornithocheirus platyrhinus*
*Ornithocheirus* sp.
*Coloborhynchus clavirostris*
*Coloborhynchus capito*
=*Ornithocheirus eurygnathus*
=*Ornithocheirus reedi*
*Coloborhynchus sedgwickii*
=*Ornithocheirus crassidens*
=*Ornithocheirus woodwardi*
*Anhanguera cuvieri*
=*Ornithocheirus brachyrhinus*
=*Ornithocheirus colorhinus*
=*Ornithocheirus dentatus*
=*Ornithocheirus denticulatus*
=*Ornithocheirus enchorhynchus*
=*Ornithocheirus scaphorhynchus*
=*Ornithocheirus xyphorhynchus*
*Anhanguera fittoni*
=*Ornithocheirus nasutus*
=*Ornithocheirus polyodon*
Lonchodectidae
*Lonchodectes giganteus*
*Lonchodectes compressirostris*
=*Ornithocheirus tenuirostris*
*Lonchodectes machaerorhynchus*
*Lonchodectes microdon*
=*Ornithocheirus huxleyi*
=*Ornithocheirus oweni*
*Lonchodectes platystomus*
=*Pterodactylus daviesii*
*Lonchodectes sagittirostris*

As part of the unpublished PhD thesis of the first author, a careful study and revision of the species referred to the Anhangueridae, Ornithocheiridae and Lonchodectidae was performed. Based on the results from this work, a review of the species from the so–called *Ornithocheirus* complex is presented here (Tables 1, 8, 9 and 10). Among these species, *Ornithocheirus huxleyi* Seeley, 1870 (misspelled *huxleyii* by Seeley [1881]) has never been figured and the holotype could not be located. Wellnhofer (1978) listed this species under the name *Ornithocheirus*, but among the species that, according to him, had uncertain systematic positions. Unwin (2001) recently synonymized it with *Lonchodectes microdon* (but see below). Here, we note these referrals but refrain from providing a discussion about it.

**Table 8.** List of taxa of the *Ornithocheirus* complex from the Cretaceous of England, after the present work. Single quotation marks indicate provisional genera. = indicate synonymies.<br/>

Ornithocheiridae Seeley, 1870
*Ornithocheirus simus* (Owen, 1861)
=*Ornithocheirus platyrhinus* Seeley, 1870
Lonchodraconidae fam. n.
*Lonchodraco giganteus* (Bowerbank, 1846) comb. n.
*Lonchodraco machaerorhynchus* (Seeley, 1870) comb. n.
*Lonchodraco(?) microdon* (Seeley, 1870) comb. n.
=*Ornithocheirus oweni* Seeley, 1870
Anhangueridae Campos and Kellner, 1985
*Coloborhynchus clavirostris* Owen, 1874
*‘Ornithocheirus’ capito* Seeley, 1870
=*Ornithocheirus reedi* Seeley, 1870
Anhangueria *incertae sedis*
*Camposipterus nasutus* (Seeley, 1870) comb. n.
*Camposipterus(?) sedgwickii* (Owen, 1859) comb. n.
*Camposipterus(?) colorhinus* (Seeley, 1870) comb. n.
Pteranodontoidea *incertae sedis*
*Cimoliopterus cuvieri* (Bowerbank, 1851) comb. n.
*‘Ornithocheirus’ polyodon* Seeley, 1870
Pterodactyloidea *incertae sedis*
*‘Ornithocheirus’ platystomus* Seeley, 1870
*‘Pterodactylus’ daviesii* Owen, 1874
*‘Ornithocheirus’ denticulatus* Seeley, 1870

**Table 9.** List of taxa of the *Ornithocheirus* complex from the Cretaceous of England here considered *nomina dubia*. <br/>

*Palaeornis cliftii* Mantell, 1844
*Cimoliornis diomedeus* Owen, 1846
*Pterodactylus compressirostris* Owen, 1851
*Pterodactylus fittoni* Owen, 1859
*Pterodactylus woodwardi* Owen, 1861
*Ornithocheirus brachyrhinus* Seeley, 1870
*Ornithocheirus carteri* Seeley, 1870
*Ornithocheirus crassidens* Seeley, 1870
*Ornithocheirus dentatus* Seeley, 1870
*Ornithocheirus enchorhynchus* Seeley, 1870
*Ornithocheirus eurygnathus* Seeley, 1870
*Ornithocheirus oxyrhinus* Seeley, 1870
*Ornithocheirus scaphorhynchus* Seeley, 1870
*Ornithocheirus tenuirostris* Seeley, 1870
*Ornithocheirus xyphorhynchus* Seeley, 1870
*Pterodactylus sagittirostris* Owen, 1874

**Table 10.** List of taxa of the *Ornithocheirus* complex from the Cretaceous of England that are *nomina nuda*. <br/>

*Ptenodactylus oweni* Seeley, 1869
*Ptenodactylus polyodon* Seeley, 1869
*Ptenodactylus microdon* Seeley, 1869
*Ptenodactylus scaphorhynchus* Seeley, 1869
*Ptenodactylus macrorhinus* Seeley, 1869
*Ptenodactylus brachyrhinus* Seeley, 1869
*Ptenodactylus crassidens* Seeley, 1869
*Ptenodactylus dentatus* Seeley, 1869
*Ptenodactylus nasutus* Seeley, 1869
*Ptenodactylus tenuirostris* Seeley, 1869
*Ptenodactylus capito* Seeley, 1869
*Ptenodactylus eurygnathus* Seeley, 1869
*Ptenodactylus machaerorhynchus* Seeley, 1869
*Ptenodactylus platystomus* Seeley, 1869
*Ptenodactylus enchorhynchus* Seeley, 1869
*Ptenodactylus colorhinus* Seeley, 1869
*Ptenodactylus oxyrhinus* Seeley, 1869
*Ornithocheirus carteri* Seeley, 1869
*Ornithocheirus platyrhinus* Seeley, 1869
*Pterodactylus curtus* Owen, 1874
*Pterodactylus nobilis* Owen, 1874
*Pterodactylus validus* Owen, 1874

In this paper, diagnoses are provided for all species and genera considered valid, in addition to photographs and illustrations, which, we hope, will facilitate future discussions about the diversity of pterosaurs in England during the Cretaceous and their relationships with species elsewhere. *Nomina nuda* are marked with double quotation marks, and single quotation marks around genera names indicate that the species is cited as in its original description, but may belong to a different genus. Rodrigues and Kellner (2008) reviewed the genus *Coloborhynchus* and its presence in the Santana Group, and the species from the Cambridge Greensand that were briefly cited in that work are also examined here.

Institutional abbreviations: BSP – Bayerische Staatssammlung für Paläontologie und historische Geologie, Munich, Germany; CAMSM – Sedgwick Museum of Earth Sciences, Cambridge, England; IVPP – Institute of Vertebrate Paleontology and Paleoanthropology, Beijing, China; MANCH – Manchester Museum, Manchester, England; MN, Museu Nacional / Universidade Federal do Rio de Janeiro, Rio de Janeiro, Brazil; NHMUK – Natural History Museum, London, England; QM – Queensland Museum, Brisbane, Australia; SMNS – Staatliches Museum für Naturkunde, Stuttgart, Germany; SMU, Shuler Museum of Paleontology, Southern Methodist University, Dallas, USA; UERJ – Universidade do Estado do Rio de Janeiro, Rio de Janeiro, Brazil; ZIN – Zoological Institute, Russian Academy of Sciences, St. Petersburg, Russia.

## Systematic palaeontology

### Pterosauria Kaup, 1834 Pterodactyloidea Plieninger, 1901

#### Ornithocheiridae

Seeley, 1870

http://species-id.net/wiki/Ornithocheiridae

Ornithocheiridae Seeley: Seeley 1870: p. 110
Criorhynchidae Hooley: Hooley 1914: p. 557

##### Type genus.

*Ornithocheirus* Seeley, 1869.

##### Included genus.

*Ornithocheirus*.

##### Recorded temporal range.

Albian.

##### Recorded stratigraphic range.

Cambridge Greensand, England.

##### Diagnosis.

the same as for the type genus.

##### Remarks.

Seeley erected the name Ornithocheirae in 1870, including only the genus *Ornithocheirus*. It is corrected to Ornithocheiridae Seeley, 1870 following the article 11.7.1.3 of the ICZN.

#### Ornithocheirus

Seeley, 1869

http://species-id.net/wiki/Ornithocheirus

Ornithocheirus Seeley: Seeley 1869: p. xvi
Ornithocheirus Seeley: Seeley 1870: p. 112
Criorhynchus Owen: Owen 1874: p. 7
Ornithochirus [sic] Seeley: Lydekker 1888: p. 10
Criorhynchus Owen: Kuhn 1967: 38

##### Type species.

*Pterodactylus simus* Owen, 1861, by monotypy.

##### Recorded temporal range.

Albian.

##### Recorded stratigraphic range.

Cambridge Greensand, England.

##### Diagnosis.

As for the type species.

#### Ornithocheirus simus

(Owen, 1861)

http://species-id.net/wiki/Ornithocheirus_simus

Figs 1 –3

**Figure 1.**
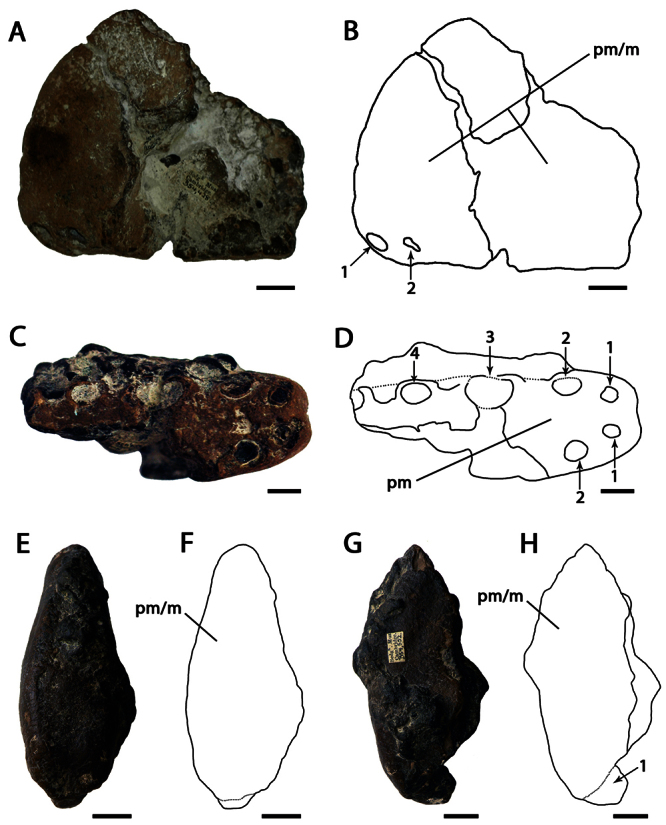
*Ornithocheirus simus*. **A–D** holotype CAMSM B54428 (Albian, Cambridge Greensand), anterior part of the rostrum **A** left lateral view **B** respective line drawing **C** ventral view **D** respective line drawing **E–H** referred specimen CAMSM B54552 (Albian, Cambridge Greensand), anterior part of the rostrum **E** anterior view **F** respective line drawing **G** left lateral view **H** respective line drawing. Abbreviations: **m** – maxillae, **pm** – premaxillae. Arrows and numbers indicate alveoli or teeth and their respective position. Scale bar = 10 mm.

**Figure 2.**
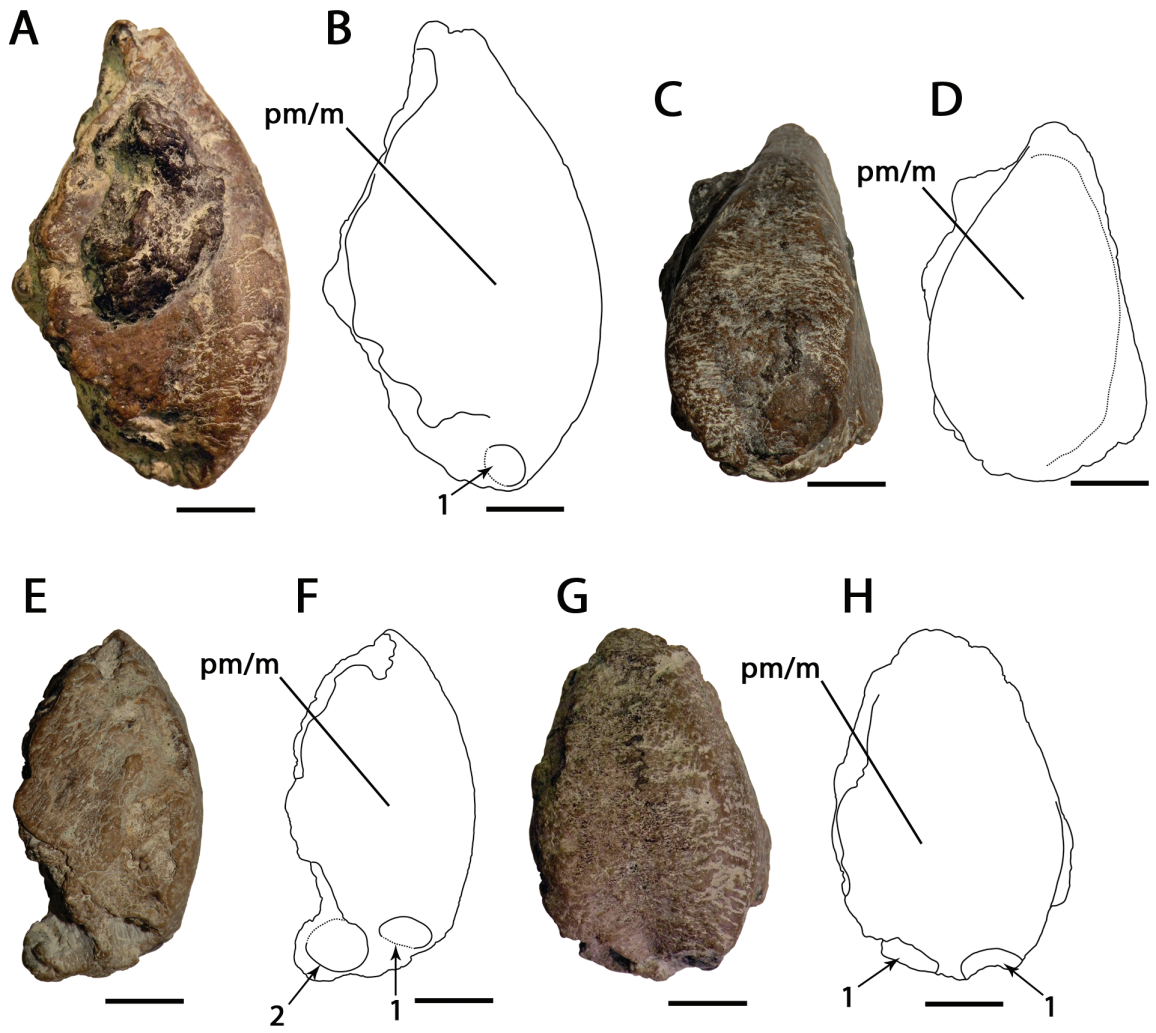
*Ornithocheirus simus*. **A–D** referred specimen CAMSM 54429 (Albian, Cambridge Greensand), anterior part of the rostrum **A** right lateral view **B** respective line drawing **C** anterior view **D** respective line drawing **E–H** referred specimen CAMSM 54677 (Albian, Cambridge Greensand), anterior part of the rostrum **E** right lateral view **F** respective line drawing **G** anterior view **H** respective line drawing. Abbreviations: **m** – maxillae, **pm** – premaxillae. Arrows and numbers indicate alveoli or teeth and their respective position. Scale bar = 10 mm.

**Figure 3.**
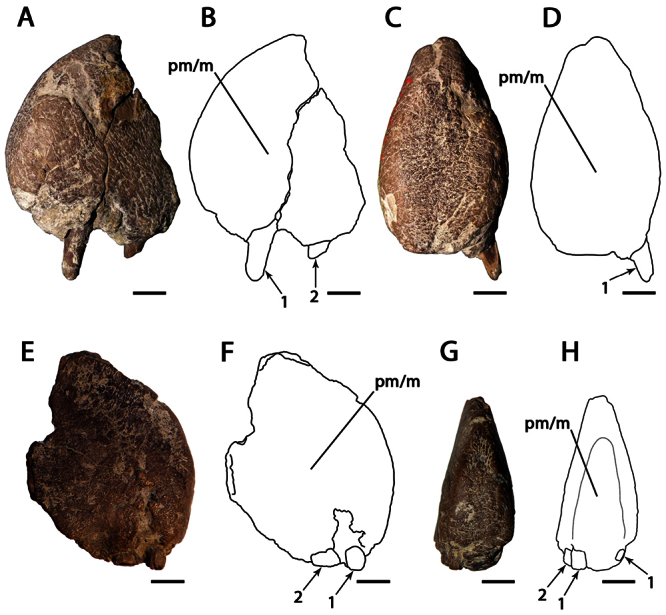
*Ornithocheirus simus*. **A–D** referred specimen MANCH L10832 (Albian, Cambridge Greensand), anterior part of the rostrum **A** left lateral view **B** respective line drawing **C** anterior view **D** respective line drawing **E–H** referred specimen NHMUK PV 35412 (Albian, Cambridge Greensand), anterior part of the rostrum **E** right lateral view **F** respective line drawing **G** anterior view **H** respective line drawing. Abbreviations: **m** – maxillae, **pm** – premaxillae. Arrows and numbers indicate alveoli or teeth and their respective position. Scale bar = 10 mm. Photos **E** and **G** courtesy of The Natural History Museum.

**Figure 4.**
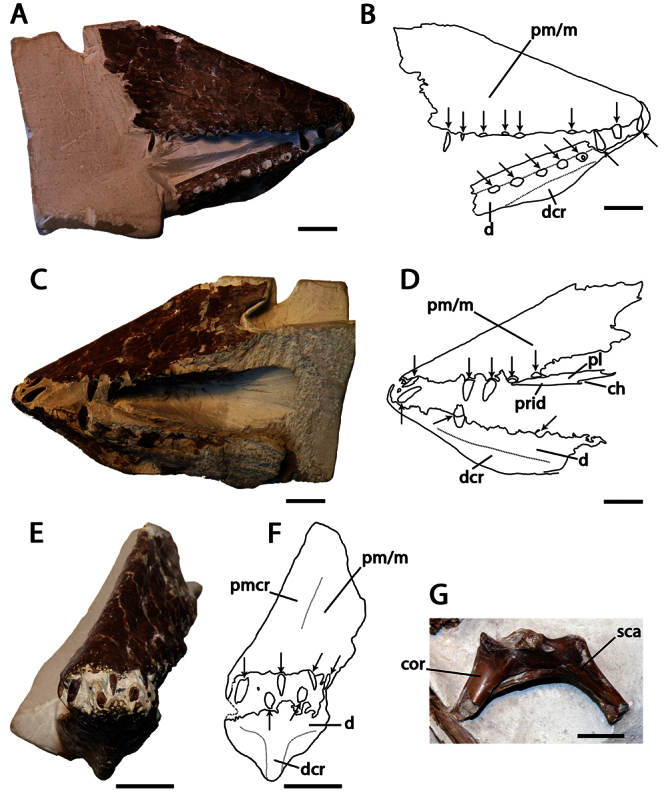
*Lonchodraco giganteus* comb. n. Lectotype NHMUK PV 39412 (Cenomanian / Turonian, Chalk Formation). **A–F** articulated anterior parts of the rostrum and mandible **A** right lateral view **B** respective line drawing **C** left lateral view **D** respective line drawing **E** anterior view **F** respective line drawing **G** associated scapulocoracoid in posterior view. Abbreviations: **ch** – choanae, **cor** – coracoid, **d** – dentary, **dcr** – dentary crest, **m** – maxillae, **pl** – palatine, **pm** – premaxillae, **pmcr** – premaxillaery crest, **prid** – palatal ridge, **sca** – scapula. Arrows indicate alveoli or teeth. Scale bar = 10 mm. Photos courtesy of The Natural History Museum.

**Figure 5.**
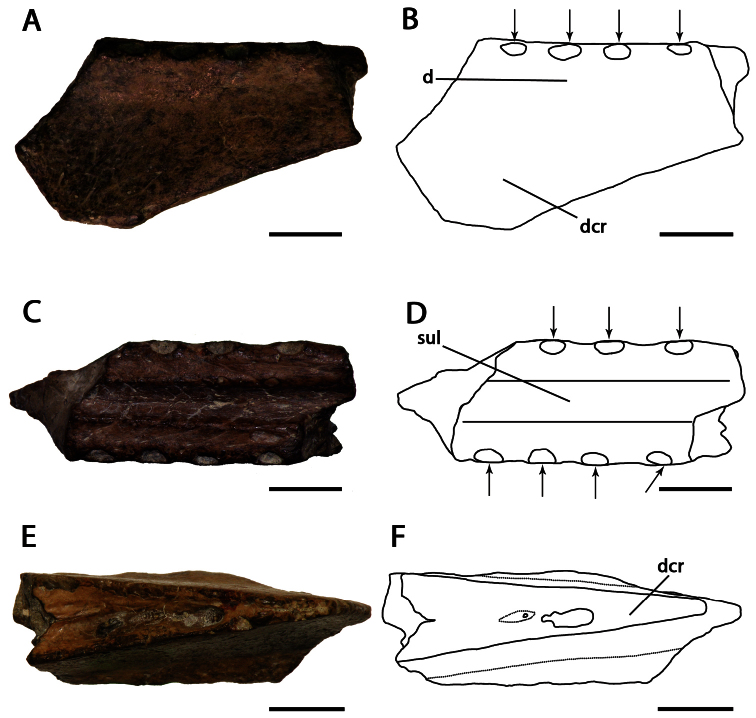
*Lonchodraco machaerorhynchus* comb. n. Holotype CAMSM B54855 (Albian, Cambridge Greensand), fragment of the mandibular symphysis **A** right lateral view **B** respective line drawing **C** dorsal view **D** respective line drawing **E** ventral view **F** respective line drawing. Abbreviations: **d** – dentary, **dcr** – dentary crest, **sul** – sulcus. Arrows indicate alveoli or teeth. Scale bar = 10 mm.

Pterodactylus simus Owen: Owen 1861: p. 2, pl. I, fig. 1–5
Ornithocheirus simus (Owen): Seeley 1869: p. xvi
Ornithocheirus simus (Owen): Seeley 1870: p. 127
Criorhynchus simus (Owen): Owen 1874: p. 7
Ornithochirus [sic](?) simus (Owen): Lydekker 1888: p. 16
Criorhynchus simus (Owen): Hooley 1914: p. 536
Criorhynchus simus (Owen): Arthaber 1922: p. 18, fig. 7a, b
Criorhynchus simus (Owen): Kuhn 1967: 38
Criorhynchus simus (Owen): Wellnhofer 1978: p. 60, fig. 8, 29
Ornithocheirus simus (Owen): Unwin 2001: p. 194, table 1
Ornithocheirus platyrhinus Seeley: Seeley 1869: p. xvii [disclaimed]
Ornithocheirus platyrhinus Seeley: Seeley 1870: p. 128
Criorhynchus platyrhinus (Seeley): Hooley 1914: p. 536
Criorhynchus simus (Owen): Wellnhofer 1978: p. 60 [synonymy]
Ornithocheirus simus (Owen): Unwin 2001: fig. 7, table 1 [synonymy]

##### Holotype.

CAMSM B54428, anterior portion of the rostrum (Fig. 1A–D).

##### Type locality.

Cambridge, Cambridgeshire, England.

##### Type horizon.

Cambridge Greensand (Cenomanian; fossils Albian in age).

##### Referred specimens.

CAMSM B54552 (Fig. 1E–H), CAMSM B54429 (Fig. 2A–D), CAMSM B54677 (Fig. 2E–H), MANCH L.10832 (Fig. 3A–D), and NHMUK PV 35412 (Fig. 3E–H) (all from the Cambridge Greensand).

##### Diagnosis.

Pterodactyloid pterosaur with the following combination of characters that distinguishes it from other members of the clade (autapomorphies are marked with an asterisk): tall rostrum*; first pair of premaxillary teeth directed ventrally*; first pair of upper alveoli slightly displaced posteriorly from the anterior margin of the premaxilla*; ventral margin of the palate straight; rostrum not expanded anteriorly.

##### Description.

*Ornithocheirus simus* was first described on the basis of a fragmentary anterior portion the premaxillae and maxillae (CAMSM B54428), which remains the best preserved specimen undoubtly referable to this species. This fragment has, in lateral view, a rounded profile, and all preserved tooth sockets are oriented ventrally. Owen (1861) described the specimen in detail and noted its very large size, that its first pair of alveoli were directed downwards, and that the anterior margin of the rostrum is tall above the first pair of alveoli. Owen (1861) thus separated it from *Pterodactylus sedgwickii* [=*Camposipterus(?) sedgwickii*, see below], in which the first pair of alveoli opens on the anterior margin of the rostrum, facing somewhat forwards, and the anterior margin of the rostrum was not as tall. Owen (1861) also noted that the anterior depression present in the holotype of *Ornithocheirus simus* was not as marked in another specimen referable to this species. Personal observations of several rostra referable to *Ornithocheirus simus* (for example, CAMSM B54429, B54552, and B54677, MANCH L.10832, and NHMUK PV 35412) suggest that this depression could have been produced by postmortem abrasion and should be avoided as a character (contra Fastnacht 2001).

*Ornithocheirus simus* lacks an anterior expansion of the rostrum. As noticed by Owen (1861), there is matrix adhered on the right side of the specimen, which can give the false impression that the palate is broader at the fourth pair of alveoli. Another interesting feature noted by Owen (1861) is the separation between the alveoli of the first pair, equivalent to the largest diameter of the alveolus, and that the bone between these two alveoli projects below from the level of the palate, but not forming an elongated structure. Owen (1861) observed that no median ridge is preserved.

In the original description and illustration, CAMSM B54428 had a tooth preserved in the first left alveolus (Owen 1861: table I, figures 1 to 5). Unwin (2001) suggested that the tooth was possibly glued in this position. During examination of the holotype in 2007, it was observed that the tooth was not preserved with the holotype anymore and could not be found.

Aside from the taxonomic and nomenclatural problems surrounding *Ornithocheirus simus*, its basic structure is controversial. Several authors considered it a long–snouted animal with a robust premaxillary crest (e.g., Wellnhofer 1987, 1991; Fastnacht 2001; Unwin 2001; Veldmeijer 2006), whereas others have suggested that it was actually a short–snouted pterosaur with a tall and massive rostrum (e.g., Hooley 1914; Arthaber 1922; Kuhn 1967; Kellner 1990). References to a reconstruction as a longirostrine pterosaur with a thick premaxillary crest are based on the alleged similarities between *Ornithocheirus simus* and the more complete holotype of *Tropeognathus mesembrinus* Wellnhofer, 1987. As the holotype and the several rostra referable to *Ornithocheirus simus* are fragmentary, it is very difficult to assess which view is correct. Therefore, we refrained from using the presence or absence of a crest in the diagnosis, but several other features (e.g., tall rostrum, position of the first pair of premaxillary teeth) support the distinctiveness of this species among the Cambridge Greensand pterosaur assemblage and in comparison with *Tropeognathus mesembrinus* (see *Taxa from other deposits*, below), leading us to propose here that Ornithocheiridae should be restricted to *Ornithocheirus simus*.

##### Remarks.

As detailed above, the taxonomic history of the genus *Ornithocheirus* and of the species *Ornithocheirus simus* is quite complex. To summarize, CAMSM B54428 was first described by Owen (1861) as *Pterodactylus simus*. Upon recognition that *Ornithocheirus simus* differed from the species of *Pterodactylus*, Seeley (1869, 1870) referred it to *Ornithocheirus*, whereas Owen (1874) subsequently transferred it to *Criorhynchus*. Both *Ornithocheirus* and *Criorhynchus* are based on the same type species (*Ornithocheirus simus*), and are therefore objective synonyms.

Seeley (1870: 128) named the species *Ornithocheirus platyrhinus* based on CAMSM B54552, an anterior portion of the rostrum (Fig. 1E–H), with the description: “another fragment, with the area very long, is marked *Ornithocheirus platyrhinus*”. The area to which Seeley (1870) referred is the tall rostrum. Even this a short characterization makes the name available. CAMSM B54552 is quite incomplete but shows features diagnostic of *Ornithocheirus simus*: tall rostrum; first pair of premaxillary teeth ventral; first pair of upper dental alveoli slightly placed back from the anterior margin of the premaxilla and ventral profile of the palate straight. Due to the fragmentary nature of this material, it cannot be determined if the anterior expansion of the rostrum was also absent or if the other alveoli have the same sizes and spacing as in CAMSM B54428. Both specimens differ slightly in size, CAMSM B54552 being approximately 7.5 cm high anteriorly and CAMSM B54428 is approximately 6.5 cm high. This difference may be due to ontogenetic or individual variation. Hence, we follow Unwin (2001) in considering *Ornithocheirus platyrhinus* a subjective junior synonym of *Ornithocheirus simus*.

#### Lonchodraconidae fam. n.

urn:lsid:zoobank.org:act:0659A30F-E4F3-4C31-8C93-C953D89493EA

http://species-id.net/wiki/Lonchodraconidae

##### Type genus.

*Lonchodraco* gen. n.

##### Included genus.

*Lonchodraco*.

##### Recorded temporal range.

Albian to Cenomanian / Turonian.

##### Recorded stratigraphic range.

Cambridge Greensand and Chalk Formation, England.

##### Diagnosis.

the same as for the type genus.

#### Lonchodraco gen. n.

urn:lsid:zoobank.org:act:21B06042-1ED5-4368-90A7-07485E87B00B

http://species-id.net/wiki/Lonchodraco

##### Etymology.

Derived from the Greek *lonchos*, meaning lance, and Latin *draco*, meaning dragon.

##### Type species.

*Pterodactylus giganteus* Bowerbank, 1846.

##### Included species.

*Lonchodraco giganteus*, *Lonchodraco machaerorhynchus*, and *Lonchodraco(?) microdon*.

##### Recorded temporal range.

Albian to Cenomanian / Turonian.

##### Recorded stratigraphic range.

Cambridge Greensand and Chalk Formation, England.

##### Diagnosis.

Pterodactyloid pterosaur with the following combination of characters that distinguishes it from other members of the clade (autapomorphies are marked with an asterisk): comparatively small alveoli (up to 4 mm in diameter) in the anterior portions of the upper and lower jaws; alveoli of the anterior portions of the upper and lower jaws without significant variation in size; alveoli placed in an elevation in relation to the palate and to the dorsal margin of the mandible*; deep palatal ridge; mandibular crest present; spacing between alveoli roughly equivalent to their diameters (modified from Unwin 2001).

##### Remarks.

Hooley (1914) created the genus *Lonchodectes*, to which he assigned nine species. Six of them were listed in alphabetical order: *Lonchodectes compressirostris*, *Lonchodectes machaeorhynchus [sic]*, *Lonchodectes microdon*, *Lonchodectes oweni*, *Lonchodectes scaphorhynchus* and *Lonchodectes tenuirostris*. Other three were cited latter and referred to the genus, still in the same publication: *Lonchodectes daviesii*, *Lonchodectes giganteus* and *Lonchodectes sagittirostris* (Hooley 1914). There was no designation of a type species.

Latter workers did not accept *Lonchodectes* as a valid genus. Kuhn (1967) and Wellnhofer (1978) considered it synonymous with *Ornithocheirus*. Kuhn (1967: 46) designated *Lonchodectes compressirostris* as the type species of the genus, using the term “Genotypus” (see above). This is a valid subsequent designation under Article 69 of ICZN. Unwin (2001), while reviewing the Cambridge Greensand pterosaurs, re–instated the genus and concluded that, of the nine species referred by Hooley (1914), only five were valid: *Lonchodectes compressirostris*, *Lonchodectes machaerorhynchus*, *Lonchodectes microdon*, *Lonchodectes giganteus* and *Lonchodectes sagittirostris*. He also added another species to the genus, *Lonchodectes platystomus*, which Hooley (1914) had placed in the genus *Amblydectes*.

In the present work, *Lonchodectes compressirostris* is considered a *nomen dubium* (see below) and, therefore,a new genus, *Lonchodraco*, is here erected to include three of the species previously referred to *Lonchodectes*: *Lonchodraco giganteus*, *Lonchodraco machaerorhynchus*, and *Lonchodraco(?) microdon*.

#### Lonchodraco giganteus

(Bowerbank, 1846) comb. n.

http://species-id.net/wiki/Lonchodraco_giganteus

Fig. 4

**Figure 6.**
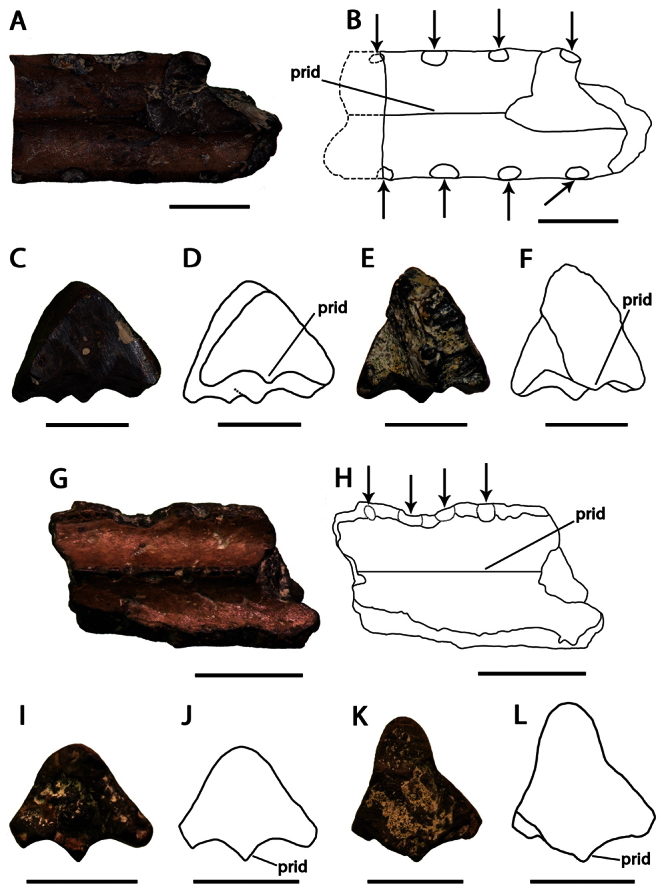
*Lonchodraco(?) microdon* comb. n. **A–F** holotype CAMSM B54486 (Albian, Cambridge Greensand), anterior fragment of the rostrum **A** ventral view **B** respective line drawing **C** anterior view **D** respective line drawing **E** posterior view **F** respective line drawing. In **B** dashed lines indicate the portion lost since the original description **G–L** referred specimen CAMSM B 54439 (Albian, Cambridge Greensand), anterior fragment of the rostrum **G** ventral view **H** respective line drawing **I** anterior view **J** respective line drawing **K** posterior view **L** respective line drawing. Abbreviation: **prid** – palatal ridge. Arrows indicate alveoli or teeth. Scale bar = 10 mm.

**Figure 7.**
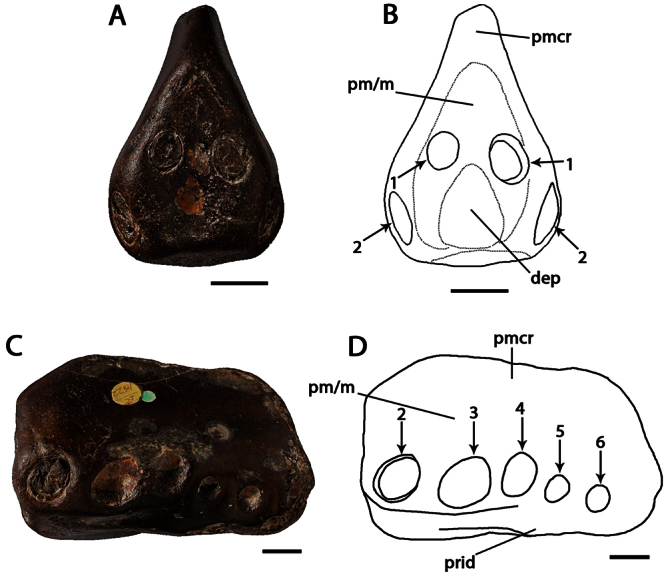
*Coloborhynchus clavirostris*. Holotype, NHMUK PV R 1822 (late Berriasian / Valanginian, Hastings Group), anterior part of the rostrum **A** anterior view **B** respective line drawing **C** left lateral view **D** respective line drawing. Abbreviations: **dep** – depression, **m** – maxillae, **pm** – premaxillae, **pmcr** – premaxillaery crest, **prid** – palatal ridge. Arrows and numbers indicate alveoli or teeth and their respective position. Scale bar = 10 mm. Photos courtesy of The Natural History Museum.

**Figure 8.**
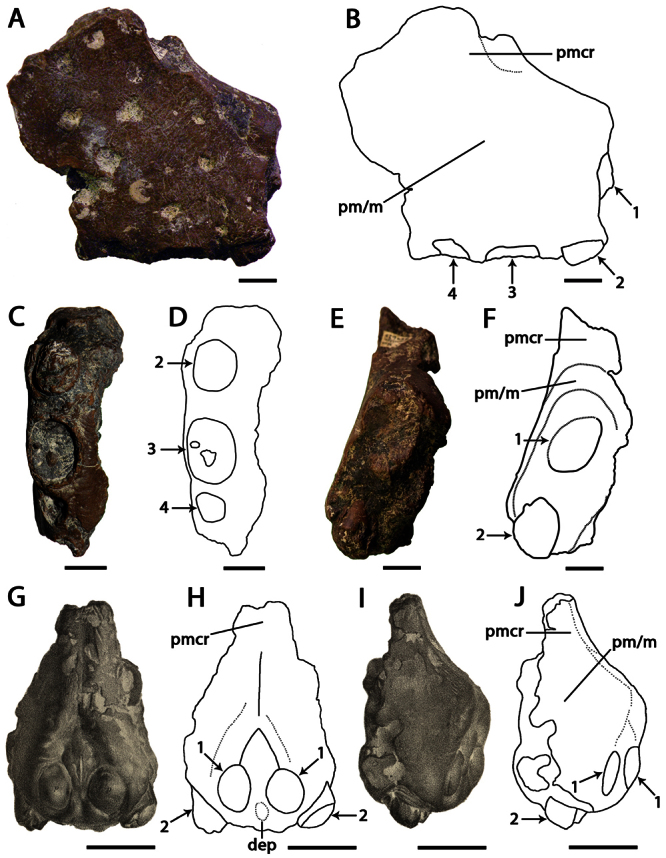
*‘Ornithocheirus’ capito*. **A–F** holotype CAMSM B 54625 (Albian, Cambridge Greensand), anterior part of the rostrum **A** right lateral view **B** respective line drawing **C** ventral view **D** respective line drawing **E** anterior view **F** respective line drawing. **G–J** referred specimen, whereabout unknown, holotype of *Ornithocheirus reedi* (Albian, Cambridge Greensand), anterior part of the rostrum **G** anterior view **H** respective line drawing **I** right lateral view **J** respective line drawing. Abbreviations: **dep** – depression, **m** – maxillae, **pm** – premaxillae, **pmcr** – premaxillary crest. Arrows and numbers indicate alveoli or teeth and their respective position. Scale bar = 10 mm. G and I from Seeley 1881.

**Figure 9.**
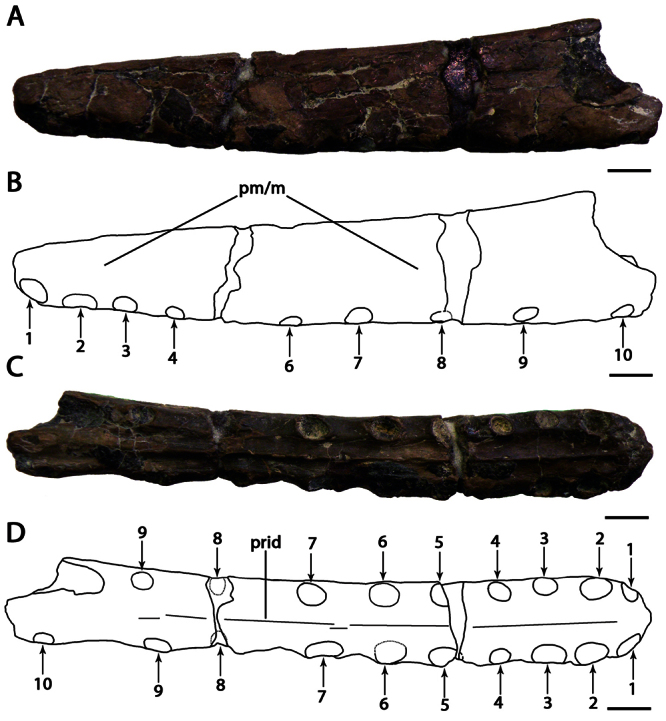
*Camposipterus nasutus* comb. n. Holotype CAMSM B 54556 (Albian, Cambridge Greensand), anterior part of the rostrum **A** left lateral view **B** respective line drawing **C** ventral view **D** respective line drawing. Abbreviations: **m** – maxillae, **pm** – premaxillae, **prid** – palatal ridge. Arrows and numbers indicate alveoli or teeth and their respective position. Scale bar = 10 mm.

Pterodactylus giganteus Bowerbank: Bowerbank 1846: p. 8, fig. 1, 2, 5.
Pterodactylus giganteus Bowerbank: Bowerbank 1848: pl. I, fig. 1.
Pterodactylus conirostris Owen: Owen in Dixon 1850: p. 401, pl. XXXVIII, fig. 4–7
Pterodactylus giganteus Bowerbank: Bowerbank 1851: p. 19
Cimoliornis diomedaeus [sic] (Gervais): Owen 1851b: p. 21
Pterodactylus giganteus Bowerbank: Owen 1851a: p. 91, pl. XXXI, fig. 1–9, 12–13
Ornithochirus [sic](?) giganteus (Bowerbank): Lydekker 1888: p. 12
Lonchodectes giganteus (Bowerbank): Hooley 1914: p. 538
Ornithodesmus(?) giganteus (Bowerbank): Arthaber 1922: p. 20, fig. 10
Ornithocheirus giganteus (Bowerbank): Wellnhofer 1978: p. 57, fig. 28
Lonchodectes giganteus (Bowerbank): Unwin 2001: p. 210

##### Lectotype.

NHMUK PV 39412, anterior portions of the rostrum and mandible, incomplete scapulocoracoid, proximal ends of the humerus and ulna, and a partial wing phalanx (Fig. 4A–G).

##### Type locality.

Near Maidstone, Burham, Kent, England.

##### Type horizon.

Chalk Formation (Cenomanian / Turonian).

##### Diagnosis.

Lonchodraconid pterosaur with the following combination of characters that distinguishes it from other members of the clade (autapomorphies are marked with an asterisk): anterior portion of the premaxillae rounded; anterior portion of the dentaries rounded; divergent alveolar margins of the anterior end of the upper and lower jaws; presence of a premaxillary crest; short, low, blade–like dentary crest*; approximately6 alveoli per 3 cm of jaw margin*.

##### Description.

*Lonchodraco giganteus* was briefly described by Bowerbank (1846), and then in more detail by Owen (1851a). The lectotype, NHMUK PV 39412, includes the anterior parts of the rostrum and mandible preserved, and, contra Bowerbank (1846) and subsequent authors (Wellnhofer 1978; Martill 2011), does not include the anterior portion of the nasoantorbital fenestra because what appears to be the anterior margin of the fenestra is not present on both sides of the specimen and most likely represents breakage. The lectotype of *Lonchodraco giganteus* is readily distinguishable from pterosaurs from other Cretaceous deposits in Britain. Owen (in Dixon 1850) described it as deep–jawed and cone–beaked. The tips of the jaws are dorsoventrally flattened, and there is no upward curvature of the palate. The alveolar margins of the upper and lower jaw are divergent even in their anterior portions. The premaxilla is tall and triangular in lateral view, indicating the presence of a crest. The crest is not thin as seen in *Anhanguera* or thick as in *Coloborhynchus* (Fastnacht 2001; Rodrigues and Kellner 2008). The mandibular symphysis also has a distinctive crest from that in anhanguerids because it does not start at the tip of the mandible. The crest is blade–like, short, and located medially in the relatively wide symphysis. Unfortunately, incomplete preparation of the specimen precludes more detailed observations of its oral region, including the palatal ridge. The mandibular groove appears to be deep but cannot be accurately measured. The teeth are conical and elongated, smaller than the ones in anhanguerids; similarly, the alveoli are small and oval to round. *Lonchodraco giganteus* has a shorter mandibular crest and a larger tooth density than *Lonchodraco machaerorhynchus* and a tall rostrum as opposed to the elongated premaxillae and maxillae in *Lonchodraco(?) microdon* and *‘Ornithocheirus’ polyodon*.

##### Remarks.

*Lonchodraco giganteus* has a complex taxonomic history. The species was named *Pterodactylus giganteus* by Bowerbank (1846). He referred several specimens to the species, including both cranial and postcranial material. Some of these specimens were found associated (NHMUK PV 39412), whereas others were not found associated but came from the same locality as the associated material; additional material was collected at different localities. It is unclear which specimen was considered the holotype. Bowerbank (1848) described the paleohistology of some bones that he referred to *Pterodactylus giganteus*, including the cranial material (NHMUK PV 39412; Bowerbank 1848: fig. (1). Owen (in Dixon 1850) proposed a new name, *Pterodactylus conirostris*, for NHMUK PV 39412, because he argued that the specimen was not gigantic in size and thus deemed the specific epithet *giganteus* inappropriate. Bowerbank (1851) responded that at the time of the description larger pterosaurs were unknown, that modifications of the names of species based on them being inappropriate would cause much instability, and refused to adopt *Pterodactylus conirostris*. Bowerbank (1851) cited the Law of Priority of the British Association Code (also known as the Strickland Code, published in 1843), which was approved by a committee that included Owen (International Commission on Zoological Nomenclature 1999; Dayrat 2010; see also Martill 2010). The Law of Priority stated that the first name of a species should be the one considered valid. Owen (1851a, b) answered Bowerbank that he had understood that the name *Pterodactylus giganteus* was proposed for a bone from the Chalk Formation that he (Owen 1842) had previously described as avian. He also pointed out that, among the material described and referred as *Pterodactylus giganteus* by Bowerbank (1846), there were at least two individuals, of very different size, the smaller one (NHMUK PV 39412) being osteologically mature (based on the fusion between scapula and coracoid), and the other one much larger. Owen (1851a, b) assumed the larger individual to be the one referred as *Pterodactylus giganteus* and thus designated *Pterodactylus conirostris* for the cranial material and the bones associated with it. He also brought up several rules of the British Association Code on which he based his designations, including exceptions to the Law of Priority in relation to inappropriate names (Owen 1851a, b; Dayrat 2010), but finally accepted the name *Pterodactylus giganteus* for the material (Owen 1851a). The name *Pterodactylus conirostris* has never been used since, but the question as to which material was the holotype of *Pterodactylus giganteus* remained overlooked for several years. Hooley (1914: 538) reviewed the species based only on the cranial material (NHMUK PV 39412). Finally, Wellnhofer (1978: 57), in his review, designated NHMUK PV 39412 as the lectotype of *Pterodactylus giganteus*, citing only the skull material and not the associated bones. *Pterodactylus giganteus* Bowerbank, 1846 and *Pterodactylus conirostris* Owen, 1850 clearly are objective synonyms because they are founded on the same type specimen, and the former binomen has priority over the latter.

#### Lonchodraco machaerorhynchus

(Seeley, 1870) comb. n.

http://species-id.net/wiki/Lonchodraco_machaerorhynchus

Fig. 5

“Ptenodactylus” machaerorhynchus Seeley: Seeley 1869: p. xvi [disclaimed]
Ornithocheirus machaerorhynvhus Seeley: Seeley 1870: p. 113, pl. XII, fig. 1–2
Lonchodectes machaeorhynchus [sic] (Seeley): Hooley 1914: p. 535
Lonchodectes machaerorhynchus (Seeley): Unwin 2001: p. 195, fig. 12D–E, table 1

##### Holotype:

CAMSM B54855, partial mandibular symphysis (Fig. 5A–F).

##### Type locality.

Cambridge, Cambridgeshire, England.

##### Type horizon.

Cambridge Greensand (Cenomanian; fossils Albian in age).

##### Diagnosis.

Lonchodraconid pterosaur with the following combination of characters that distinguishes it from other members of the clade (autapomorphies are marked with an asterisk): deep dentary crest*; ventral margin of the mandible posterior to the dentary crest ascending in lateral view*; ventral depression located posteriorly to the dentary crest*; wide mandibular groove*; approximately 4.5 alveoli per 3 cm of jaw margin.

##### Description.

CAMSM B54855 consists of a fragment of the posterior portion of the mandibular symphysis. Seeley (1870) described the mandible as narrow, with parallel alveolar margins, deep mandibular sulcus, spacing between alveoli equal to the size of their diameters, and with the lateral margins forming a sharp keel, which Unwin (2001) interpreted as a dentary crest. Seeley (1870) mentioned the presence of a suture with the angular bone, which would almost reach the tip of the mandible, as a unique character for this species in comparison to the pterosaurs from Germany. We here interpret this structure as the posterior margin of a dentary crest rather than a suture.

In addition to a deep mandibular groove, CAMSM B54855 shares with *Lonchodraco giganteus* small and well–spaced alveoli, without significant size variation. However, it differs from this species in having straight alveolar margins in dorsal view (Seeley 1870), whereas the margins diverge in *Lonchodraco giganteus*. The mandibular crest of *Lonchodraco machaerorhynchus* is deep and its apex coincident with its terminus, with a ventral depression posterior to it. In *Lonchodraco giganteus*, the mandibular crest is short and blade–like, being more restricted to the length of the symphysis; the presence of a depression cannot be determined.

#### Lonchodraco(?) microdon

(Seeley, 1870) comb. n.

Fig. 6

“Ptenodactylus” microdon Seeley: Seeley 1869: p. xvi [disclaimed]
Ornithocheirus microdon Seeley: Seeley 1870: p. 116, pl. XII, fig. 6–7
Lonchodectes microdon (Seeley): Hooley 1914: p. 535
Ornithocheirus microdon Seeley: Wellnhofer 1978: p. 58
Lonchodectes microdon (Seeley): Unwin 2001: p. 211, table 1
Ornithocheirus oweni Seeley: Seeley 1870: p. 115
Lonchodectes oweni (Seeley): Hooley 1914: p. 535
Lonchodectes microdon (Seeley): Unwin 2001: p. 195, fig. 11C–D, table 1 [synonymy]

##### Holotype.

CAMSM B54486, anterior portion of the rostrum (Fig. 6A–F).

##### Type locality.

Cambridge, Cambridgeshire, England.

##### Type horizon.

Cambridge Greensand (Cenomanian; fossils Albian in age).

##### Referred specimen.

CAMSM B 54439 (Fig. 6G–L) (also from the Cambridge Greensand).

##### Diagnosis.

Lonchodraconid pterosaur with the following combination of characters that distinguishes it from other members of the clade (autapomorphies are marked with an asterisk): premaxillary crest absent; dorsal margin of the premaxillae rounded; deep palatal ridge*; palate between the elevation of the alveolar margins and the palatal ridge concave; spacing between alveoli larger than their diameters*; approximately 4.5 alveoli per 3 cm of jaw margin.

##### Description.

*Lonchodraco(?) microdon* was described by Seeley (1870) on the basis of CAMSM B54486, a fragmentary upper jaw with a prominent palatal ridge. Seeley (1870) noted the presence of a concavity on the palate between the elevation of the alveolar margins and the palatal ridge, that the palatal ridge becomes more prominent than the alveolar borders posteriorly, and that the alveolar margins are compressed and rounded. Seeley (1870) also noted a small tip of jaw “associated” with the specimen, but this possibly does not represent the same individual. CAMSM B54486 was recently sampled for histological analysis (M. Riley, pers. comm.). The transverse section of the area that was cut is wider than high, whereas the opposite end is higher than wide, suggesting that the sampled area was located near the anterior end of the snout.

*Ornithocheirus oweni* was described in the same work (Seeley 1870) on the basis of CAMSM B 54439 (Fig. 6G–L), also an upper jaw. This species is quite similar to *Lonchodraco(?) microdon*. Both holotypes share a rostrum with parallel alveolar margins, absence of a premaxillary crest, rounded dorsal margin of the premaxillae and maxillae, deep palatal ridge and the concave palate between the alveolar margins and the palatal ridge. Seeley (1870) pointed out that *Ornithocheirus oweni* differs from *Lonchodraco(?) microdon* in that its jaw margins are not round but flattened, by the presence of rough interspaces between the alveoli, teeth (alveoli?) circular instead of oval, and the dorsal margin of the premaxillae and maxillae rounded instead of having a sharp keel. However, examination of CAMSM B 54439 established that the alveolar margins are poorly preserved so that the number, shape, and spacing of the alveoli cannot be seen. Furthermore, *Lonchodraco(?) microdon* has a rounded dorsal margin of the rostrum, as can be observed in anterior and posterior transverse sections of the holotype. As both species share the same morphological features and come from the same deposit they are here considered conspecific.

##### Remarks.

Unwin (2001) synonymized *Ornithocheirus oweni* with *Lonchodraco(?) microdon* and, acting as the First Reviser (ICZN’s article 24.2.2), gave *Lonchodraco(?) microdon* priority.

*Lonchodraco(?) microdon* has small (approximately 3 mm diameter) and evenly spaced alveoli as in *Lonchodraco giganteus* and *Lonchodraco machaerorhynchus*. However, it is distinct from *Lonchodraco giganteus*, lacking a premaxillary crest, having spacing between the alveoli larger than their diameters, and with parallel alveolar margins. These features constrast with those of the type species of the genus. *Lonchodraco(?) microdon* shares with *Lonchodraco machaerorhynchus* the parallel alveolar margins and the same alveolar density (4.5 alveoli per 3 cm of jaw margin), but they differ in the spacing between the alveoli, larger in the first. Due to these differences, it is possible that *Lonchodraco(?) microdon* actually represents a distinct genus. However, in the absence of further evidence, we refrain from naming a new genus and assign the species tentatively to *Lonchodraco*.

#### Anhangueria new clade

##### Branch–based definition.

All pteranodontoids more closely related to *Anhanguera blittersdorffi* than to *Istiodactylus latidens* and *Cimoliopterus cuvieri*.

##### Content.

*Camposipterus*, *Cearadactylus*, *Ludodactylus*, and Anhangueridae.

##### Recorded temporal range.

Late Berriasian / Valanginian to Cenomanian.

##### Recorded stratigraphic range.

Hastings Group, England; Wessex Formation, England; Elrhaz Formation, Niger; Jiufotang Formation, China; Khuren–Dukh, Dzun–Bayin Formation, Mongolia; Romualdo Formation, Brazil; Paw Paw Formation, USA; Cambridge Greensand, England; Kem Kem beds, Morocco.

##### Synapomorphies.

(18.1) Presence of an anterior expansion of the premaxillary tip with the jaw end tall, and (48.1) larger teeth located at the tip of the rostrum (see “Phylogenetic affinities of the species of the *Ornithocheirus* complex”, below).

#### Anhangueridae

Campos & Kellner, 1985

http://species-id.net/wiki/Anhangueridae

Anhangueridae Campos & Kellner: Campos and Kellner 1985: p. 459
Ornithocheiridae Seeley: Unwin 2001: p. 205

##### Type genus.

*Anhanguera* Campos and Kellner, 1985.

##### Included genera.

*Anhanguera*, *Caulkicephalus*, *Coloborhynchus*, *Liaoningopterus*, *Siroccopteryx*, *Tropeognathus*, and *Uktenadactylus*.

##### Recorded temporal range.

Late Berriasian / Valanginian to Cenomanian.

##### Recorded stratigraphic range.

Hastings Group, England; Wessex Formation, England; Elrhaz Formation, Niger; Jiufotang Formation, China; Khuren–Dukh, Dzun–Bayin Formation, Mongolia; Romualdo Formation, Brazil; Paw Paw Formation, USA; Cambridge Greensand, England; Kem Kem beds, Morocco (Table 11).

**Table 11.** Deposits where anhanguerid fossils have been found.<br/>

Late Berriasian/Valanginian	Hastings Group, England (*Coloborhynchus clavirostris*; see Owen 1874)
Barremian	Wessex Formation, England (*Caulkicephalus trimicrodon*; see Steel et al. 2005)
Aptian	Elrhaz Formation, Niger (Blackburn 2002)<br/> Jiufotang Formation, China (*Liaoningopterus gui*; see Wang and Zhou 2003)
Aptian/Albian	Khuren–Dukh, Dzun–Bayin Formation, Mongolia (Bakhurina and Unwin 1995) (spelled Hüren–Dukh, Züünbayan Formation by Unwin and Bakhurina 2000)
Albian	Romualdo Formation, Brazil (*Tropeognathus mesembrinus* and several species of *Anhanguera*; e.g., Campos and Kellner 1985)<br/> Paw Paw Formation, USA (*Uktenadactylus wadleighi*; see Lee 1994)<br/> Cambridge Greensand*, England (see Unwin 2001; the present work)
Cenomanian	Kem Kem beds, Morocco (*Siroccopteryx moroccensis*; see Mader and Kellner 1999)

* this deposit is Cenomanian but the fossils are Albian in age.

#### Coloborhynchus

Owen, 1874

http://species-id.net/wiki/Coloborhynchus

##### Type species:

*Coloborhynchus clavirostris* Owen, 1874, by monotypy.

##### Recorded temporal range.

Late Berriasian / Valanginian.

##### Recorded stratigraphic range.

Hastings Group, England.

##### Diagnosis.

As for the type species.

#### Coloborhynchus clavirostris

Owen, 1874

http://species-id.net/wiki/Coloborhynchus_clavirostris

Fig. 7

Coloborhynchus clavirostris Owen: Owen 1874: p. 6, pl. I, fig. 1–4
Criorhynchus simus (Owen): Hooley 1914: p. 537
Criorhynchus clavirostris (Owen): Arthaber 1922: p. 18, fig. 7c
Criorhynchus simus (Owen): Kuhn 1967: 38
Criorhynchus simus (Owen): Wellnhofer 1978: p. 60
Coloborhynchus clavirostris Owen: Lee 1994: p. 756, fig. 4
Coloborhynchus clavirostris Owen: Unwin 2001: p. 206
Coloborhynchus clavirostris Owen: Veldmeijer 2003: 42
Coloborhynchus clavirostris Owen: Rodrigues and Kellner 2008: p. 220, fig. 1.1, 2.1, 3.1
Coloborhynchus clavirostris Owen: Martill, Sweetman and Witton 2011: p. 380, fig. 25.8

##### Holotype.

NHMUK PV R 1822, anterior portion of the rostrum (Fig. 7A–D).

##### Type locality.

St.–Leonards–on–Sea, East Sussex, England.

##### Type horizon.

Hastings Group (late Berriasian / Valanginian).

##### Diagnosis.

Anhanguerid pterosaur with the following combination of characters that distinguishes it from other members of the clade (autapomorphies are marked with an asterisk): oval depression beneath the first pair of alveoli*; second, third and fourth pairs of alveoli located laterally*; fifth and sixth pairs of alveoli located more medially than the preceding alveoli on the base of the palatal ridge*; anterior part of the palatal ridge bordered by two shallow longitudinally elongated depressions* (from Rodrigues and Kellner 2008).

##### Description.

The holotype of *Coloborhynchus clavirostris* (NHMUK PV R 1822) is a fragment of the premaxillae and maxillae, and has previously been described in detail (Owen 1874; Veldmeijer 2003; Rodrigues and Kellner 2008). It has a flattened anterior margin, where the first pair of tooth sockets is located. The second, third and fourth pairs of alveoli face laterally, and the fifth and sixth pairs are situated closer to the midline. *Coloborhynchus clavirostris* also has an anterior expansion and a strong palatal ridge in addition a robust premaxillary crest, which begins at the tip of the rostrum.

##### Remarks.

Rodrigues and Kellner (2008) reviewed the taxonomic history of the genus *Coloborhynchus* and the species *Coloborhynchus clavirostris*, and thus only the main points are repeated here. The genus and species were erected by Owen (1874). Later authors such as Hooley (1914), Kuhn (1967) and Wellnhofer (1978) regarded it synonymous with *Ornithocheirus simus* [=*Criorhynchus simus*]. Lee (1994) revalidated *Coloborhynchus*, and Unwin (2001) not only considered it a valid genus, but also referred additional species to the genus. Rodrigues and Kellner (2008), noting several unique characters of *Coloborhynchus clavirostris*, restricted the genus to the type species. This view is followed here.

#### ‘Ornithocheirus’ capito

Seeley, 1870

Fig. 8

“Ptenodactylus” capito Seeley: Seeley 1869: p. xvi [disclaimed]
Ornithocheirus capito Seeley: Seeley 1870: p. 126
Criorhynchus capito (Seeley): Hooley 1914: p. 536
Criorhynchidae *incertae sedis*: Wellnhofer 1978: 60
Coloborhynchus capito (Seeley): Unwin 2001: p. 206, table 1
Ornithocheirus ” *capito* Seeley: Rodrigues and Kellner 2008: p. 226, fig. 4.1, 4.3 “
Ornithocheirus reedi Seeley: Seeley 1870: p. 126
Ornithocheirus reedii [sic] Seeley: Seeley 1881: p. 13, pl. I, fig. 3
Criorhynchus reedi (Seeley): Hooley 1914: p. 538
Criorhynchus reedii [sic] (Seeley): Arthaber 1922: p. 18
Criorhynchidae *incertae sedis*Wellnhofer 1978: 60
Coloborhynchus capito (Seeley): Unwin 2001: fig. 6C, 8, table 1 [synonymy]

##### Holotype.

CAMSM B 54625, anterior portion of the rostrum (Fig. 8A–F).

##### Type locality.

Chesterton, Cambridgeshire, England.

##### Type horizon.

Cambridge Greensand (Cenomanian; fossils Albian in age).

##### Referred specimen.

Holotype of *Ornithocheirus reedi* Seeley, 1870 (Fig. 8G–J) (from the Cambridge Greensand; current whereabouts unkown).

##### Diagnosis.

Anhanguerid pterosaur with the following combination of characters that distinguishes it from other members of the clade: anteriorly located and tall premaxillary crest; anterior margin of the premaxillary crest concave in lateral view; first pair of upper teeth positioned on the anterior margin of the rostrum.

##### Description.

*‘Ornithocheirus’ capito* is known from a fragmentary holotype, which has a tall, anteriorly located premaxillary crest with a concave anterior margin. Due to its fragmentary state, the presence of an anterior expansion cannot be confirmed but is suggested by the structure of the preserved right side of the specimen. These features allow its placement in Anhangueridae.

A second specimen referable to this species is the holotype of *Ornithocheirus reedi*. It has a median groove that extends along the height of the crest; such groove is usually considered a sign of abrasion among Cambridge Greensand pterosaurs, but Seeley (1881) affirmed that the bone surface is rather smooth, and abrasion is unlikely. Furthermore, a groove in the midline of the crest is present in the holotype of *Uktenadactylus wadleighi* (SMU 73058) from the Albian Paw Paw Formation of the USA (Lee 1994; Rodrigues and Kellner 2008), which also has a concave anterior margin of the crest. However, *‘Ornithocheirus’ capito* differs from SMU 73058 in the height of the crest, which is much higher just behind the second pair of alveoli.

*‘Ornithocheirus’ capito* differs from *Coloborhynchus clavirostris* (from a distinct, older deposit, the late Berriasian–Valanginian Hastings Group of the Wealden Supergroup) in the absence of a flat anterior margin of the rostrum. Rodrigues and Kellner (2008) listed four autapomorphies for *Coloborhynchus clavirostris*, none of which is present in *‘Ornithocheirus’ capito*. It also can be distinguished from *Uktenadactylus wadleighi* in the absence of an oval depression above the first pair of teeth (Lee 1994; Rodrigues and Kellner 2008).

*‘Ornithocheirus’ capito* can also be distinguished from *Ornithocheirus simus*, and from *Ornithocheirus*, by presence of the first pair of teeth in the anterior margin of the premaxillae. In *Ornithocheirus simus*, the first pair of alveoli is directed ventrally and not located at the tip of the snout. The combination of features seen in *‘Ornithocheirus’ capito* is also absent in *Lonchodraco*, *Cimoliopterus*, *Camposipterus* (see below), or any other known pterosaur, and is diagnostic for the present species even though this species has no autapomorphies.

##### Remarks.

Unwin (2001) synonymized *‘Ornithocheirus’ reedi* with *‘Ornithocheirus’ capito* (as *Coloborhynchus capito*). *‘Ornithocheirus’ reedi*, described by Seeley (1870),was referred by Hooley (1914) and provisionally by Wellnhofer (1978) to *Criorhynchus reedi*, and its name was misspelled *reedii* by Seeley (1881) and *readi* by Barrett et al. (2008). Its holotype, illustrated by Seeley (1881: pl. I, figs 3a–3b; here as Fig. 8E–H), belonged to the collection of W. Reed of York by the time it was described and could not be found in the collections of the Natural History Museum, the Sedgwick Museum of Earth Sciences or the Manchester Museum during visits in October 2009. Therefore, we base our remarks on the descriptions and illustrations provided by Seeley (1870, 1881).

Seeley (1870) first described *Ornithocheirus reedi* and interpreted the holotype as an upper jaw. Later Seeley (1881) redescribed the specimen as a lower jaw, based on the presence of a median groove. Curiously, in the same work, it was illustrated oriented as being a part of a snout. Unfortunately, the specimen was not figured in palatal view, but it is very tall and we here interpret it as the tip of the premaxillae. It bears a median crest with a markedly concave anterior margin. As *‘Ornithocheirus’ capito* and *Ornithocheirus reedi* share this feature (unknown in other Cambridge Greensand species) and come from the same deposit, we agree with Unwin (2001) that they represent the same taxon. Both were described in the same work (Seeley 1870). Acting as First Reviewer, Unwin (2001) gave priority to the former binomen.

Here, we recognize that *‘Ornithocheirus’ capito* possibly represents a new genus, but we refrain from naming a new one until better material comes to light. Therefore, we refer to it by the name given in its original description, as *‘Ornithocheirus’ capito*.

### Anhangueria *incertae sedis*

#### Camposipterus gen. n.

urn:lsid:zoobank.org:act:A2644A0F-2C01-4BF8-A558-BAFEBADBC7EF

http://species-id.net/wiki/Camposipterus

##### Etymology.

After the Brazilian paleontologist Diogenes de Almeida Campos, who made valuable contributions to the knowledge of pterosaur diversity in Brazil and was a major influence to us, and *pterus*, from the Greek *pteron*, meaning wing.

##### Type species.

*Ornithocheirus nasutus* Seeley, 1870.

##### Included species.

*Camposipterus nasutus*, *Camposipterus(?) sedgwickii* and *Camposipterus(?) colorhinus*.

##### Recorded temporal range.

Albian.

##### Recorded stratigraphic range.

Cambridge Greensand, England.

##### Diagnosis.

Pterodactyloid pterosaurs with the following combination of characters that distinguishes it from other members of the clade: anterior tip of the premaxillae and maxillae round in lateral view; premaxillary crest absent; anterior expansion of the rostrum present; palate curving dorsally; first pair of alveoli located anteriorly.

#### Camposipterus nasutus

(Seeley, 1870) comb. n.

http://species-id.net/wiki/Camposipterus_nasutus

Fig. 9

“Ptenodactylus” nasutus Seeley: Seeley 1869: p. xvi [disclaimed]
Ornithocheirus nasutus Seeley: Seeley 1870: p. 120
Ornithocheirus nasutus Seeley: Hooley 1914: p. 535
Anhanguera fittoni (Owen): Unwin 2001: fig. 10C–E, table 1 [synonymy]

##### Holotype.

CAMSM B 54556, anterior portion of the rostrum (Fig. 9A–D).

##### Type locality.

Haslingfield, Cambridgeshire, England.

##### Type horizon.

Cambridge Greensand (Cenomanian; fossils Albian in age).

##### Diagnosis.

Pterodactyloid pterosaur with the following combination of characters that distinguishes it from other members of the clade (autapomorphies are marked with an asterisk): dorsal margin of the rostrum straight to gently concave in lateral view; palatal ridge extends anteriorly until just posterior to the second pair of alveoli; spacing between alveoli irregular, with the anterior alveoli closer and the posterior ones more distant from each other; density of almost 3 alveoli each 3 cm anteriorly and 2,5 alveoli each 3 cm posteriorly*; tip of the rostrum dorsoventrally flattened, wider than high in anterior view*; second and third alveoli face lateroventrally; anterior portion of the premaxillae slightly expanded.

##### Description.

*Camposipterus nasutus* was originally described by Seeley (1870) as *Ornithocheirus nasutus*. Seeley noted that it has an expansion at the tip of the rostrum, a palatal ridge extending posteriorly to the level of the second pair of alveoli, the first pair of alveoli facing forward, and a dorsoventrally compression of the tip of the rostrum. It differs from *Cimoliopterus cuvieri*, which possesses a premaxillary crest but no anterior expansion of the rostrum, and which is higher than wide in anterior view, in contrast with the wider than high tip of the rostrum of *Camposipterus nasutus*.

##### Remarks.

Unwin (2001) synonymized the species with *Anhanguera fittoni* [=*Pterodactylus fittoni*, here considered a *nomen dubium*, see below]. We do not agree with this view because the holotype of *Camposipterus nasutus* is dorsoventrally flattened and has an anterior expansion. By contrast, *Pterodactylus fittoni* is known from a fragmentary rostrum that, although incomplete anteriorly, does not share these features. It can also be excluded from *Anhanguera* because it does not have a premaxillary crest; furthermore, no species definitely referable to *Anhanguera* has a dorsoventrally flattened rostrum. It can be expected that the description of new, more complete specimens from the Romualdo Formation of the Santana Group, currently under work by several researchers, will help shed light in its relationships with taxa such as *Brasileodactylus araripensis* Kellner, 1984 (see Kellner 1984), but so far the dorsoventrally flattened anterior end of the rostrum seems to be diagnostic for *Camposipterus nasutus*.

#### Camposipterus(?) sedgwickii

(Owen, 1859) comb. n.

Fig. 10A–D

Pterodactylus sedgwickii Owen: Owen 1859: p. 2, pl. I, fig. 1
“Ptenodactylus” sedgwicki [sic] (Owen): Seeley 1869: p. xvi [disclaimed]
Ornithocheirus sedgwicki [sic] (Owen): Seeley 1870: p. 112
Coloborhynchus sedgwickii (Owen): Owen 1874: p. 6
Ornithochirus [sic] sedgwicki [sic] (Owen): Lydekker 1888: p. 15
Ornithocheirus sedgwicki [sic] (Owen): Hooley 1914: p. 535
Ornithocheirus sedgwicki [sic] (Owen): Arthaber 1922: p. 17
Ornithocheirus sedgwicki [sic] (Owen): Wellnhofer 1978: p. 58, fig. 28
Coloborhynchus sedgwickii (Owen): Unwin 2001: p. 194, fig. 9, table 1
“Ornithocheirus” sedgwickii (Owen): Rodrigues and Kellner 2008: p. 226, fig. 4.2, 4.4

##### Holotype.

**Figure 10.**
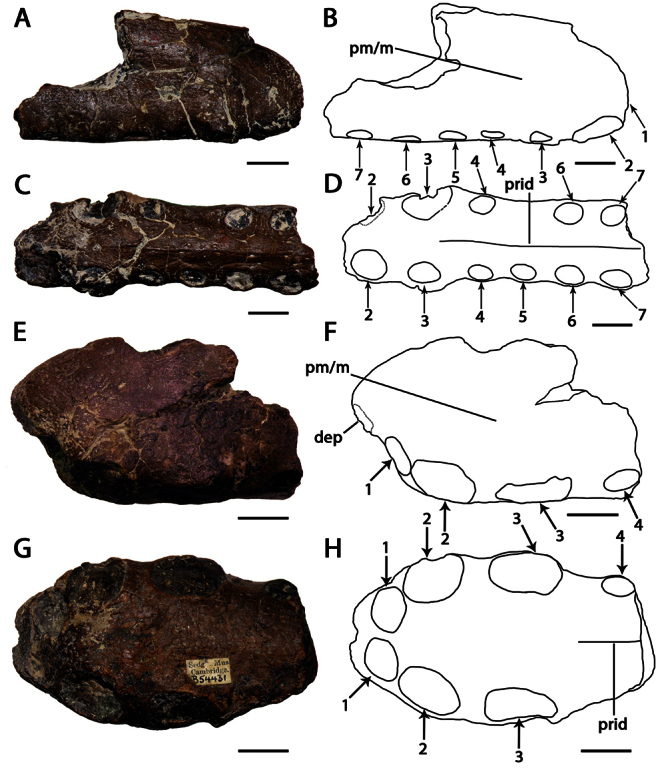
**A–D** *Camposipterus(?) sedgwickii* comb. n., holotype CAMSM B54422 (Albian, Cambridge Greensand), anterior part of the rostrum. **A** right lateral **B** respective line drawing **C** ventral view **D** respective line drawing. **E–H** *Camposipterus(?) colorhinus* comb. n., syntype CAMSM B54431 (Albian, Cambridge Greensand), anterior part of the rostrum **E** left lateral view **F** respective line drawing **G** ventral view **H** respective line drawing. Abbreviations: **dep** – depression, **m** – maxillae, **pm** – premaxillae, **prid** – palatal ridge. Arrows and numbers indicate alveoli or teeth and their respective position. Scale bar = 10 mm.

CAMSM B54422, anterior part of the rostrum (Fig. 10A–D).

##### Type locality.

Cambridge, Cambridgeshire, England.

##### Type horizon.

Cambridge Greensand (Cenomanian; fossils Albian in age).

##### Diagnosis.

Pterodactyloid pterosaur with the following combination of characters that distinguishes it from other members of the clade (autapomorphies are marked with an asterisk): tall rostrum; anterior expansion of the rostrum ends abruptly behind the third pair of alveoli*; palatal ridge extending posteriorly to the level of the third pair of alveoli; third pair of alveoli much larger than fourth*.

##### Description.

Owen (1859) described *Pterodactylus sedgwickii* based on an upper jaw, CAMSM B54422. He pointed out the presence of the first pair of alveoli at the anterior margin of the rostrum, facing forward; an anterior expansion of the rostrum, where the large second and third pairs of alveoli are located; alveoli four to six approximately same size; spacing between alveoli smaller than their diameter; and obtuse tip of the rostrum.

The third alveoli have different sizes on the left and right sides; this could be explained on taphonomic grounds (e.g., Kellner 2010). On the left side, the bone surrounding the alveolus seems to have been forced outwards, perhaps by phosphate deposition (Seeley 1870). On the other hand, the absence of the fifth alveolus on the left side, as noted by Owen (1859), is a possibly pathological feature seen in other pterosaurs, such as in the holotype of *Anhanguera robustus* (BSP 1987 I 47) and in a specimen referred to *Anhanguera blittersdorffi* (Pz–DBAV–UERJ 40).

It is noteworthy that the drawing of CAMSM B54422 was reversed in Owen (1859) and in Wellnhofer (1978) and Unwin (2001), which present illustrations based on Owen’s. Also, there is a breakage and a discontinuity between the anterior, round part of the rostrum and the more posteriorly located dorsal margin, which is not figured by the aforementioned authors.

Owen (1859) referred a lower jaw, CAMSM B54421, to the same species, partially based on a similar alveolar density as in the holotype. CAMSM B54421 is very fragmentary and does not have the tip of the mandible preserved. Furthermore, these two specimens do not fit together, with the upper jaw much broader than the lower one. There is no evidence to support placement of CAMSM B54421 in *Camposipterus(?) sedgwickii*, and we here restrict the species to its type material.

##### Remarks.

Hooley (1914) and Wellnhofer (1978) placed *Camposipterus(?) sedgwickii* in *Ornithocheirus*. It differs from this genus in the absence of a tall rostrum, the first pair of teeth facing ventrally, and other characters (see above). Owen (1874) and Unwin (2001) referred it to *Coloborhynchus*. Rodrigues and Kellner (2008) excluded *Camposipterus(?) sedgwickii* from *Coloborhynchus*, as it differs from *Coloborhynchus clavirostris* in that the anterior end of the rostrum is round rather than flat and the spacing between the first and second pairs of alveoli is larger in *Coloborhynchus clavirostris*. *Camposipterus(?) sedgwickii* shows none of the autapomorphies listed for *Coloborhynchus clavirostris*. Therefore, we tentatively place this species in the genus *Camposipterus*. *Camposipterus nasutus* and *Camposipterus(?) sedgwickii* share the presence of an expansion of the rostrum, absence of a premaxillary crest, and round rostrum in lateral view. They differ in the different height of the tip of the rostrum, which is taller than wide in *Camposipterus(?) sedgwickii*, the extension of the palatal ridge, and the size of the expansion, which is larger in *Camposipterus(?) sedgwickii* than in *Camposipterus nasutus*.

#### Camposipterus(?) colorhinus

(Seeley, 1870) comb. n.

Fig. 10E–H

“Ptenodactylus” colorhinus Seeley: Seeley 1869: p. xvi [disclaimed]
Ornithocheirus colorhinus Seeley: Seeley 1870: p. 124
Ornithocheirus colorhinus Seeley: Hooley 1914: p. 535
Anhanguera cuvieri (Bowerbank): Unwin 2001: fig. 10A, B, table 1

##### Syntypes.

CAMSM B54431 (Fig. 10E–H) and CAMSM B54432, anterior parts of the rostrum.

##### Type locality.

Cambridge, Cambridgeshire, England.

##### Type horizon.

Cambridge Greensand (Cenomanian; fossils Albian in age).

##### Diagnosis.

Pterodactyloid pterosaur with the following combination of characters that distinguishes it from other members of the clade (autapomorphies are marked with an asterisk): developed anterior expansion, lacking a marked constriction; presence of a depression above the first pair of alveoli; anterior depression faces anteroventrally*; second and third alveoli very large in size; fourth pair of alveoli much smaller than the second and third.

##### Description.

The syntypes, CAMSM B54431 (the more complete one) and CAMSM B54432, both are anterior portions of upper jaws. They are quite incomplete and abraded, but they are identical where comparable and demonstrate that their features are valid morphological characters rather than taphonomic artifacts. Seeley (1870) pointed out the presence of a slightly convex median part of the palate (seen in CAMSM B54431), an anterior expansion of the rostrum, and the large, round alveoli, the first of which faces anteroventrally. Seeley (1870) interpreted the presence of a lunate area as attachment for a lip. We verified this observation but question his interpretation, as the tips of the jaws of pterosaurs were covered by a horny beak (Seeley 1901: fig. 20; Kellner and Tomida 2000; Frey et al. 2003; Pinheiro et al. 2011).

This lunate area is a depression above the first pair of alveoli; a depression in the same location is also present in *Uktenadactylus wadleighi* but in the latter the anterior margin of the rostrum faces anteriorly (Lee 1994; Rodrigues and Kellner 2008), whereas in *Camposipterus(?) colorhinus* this edge faces anteroventrally and, consequently, the orientation of the depression is also different. Furthermore, the first pair of teeth in *Uktenadactylus* is located higher and the anterior expansion is more squarish and not round as in the species under discussion.

##### Remarks.

Unwin (2001) referred to CAMSM B54431 as “a large individual of *Anhanguera cuvieri*” [=*Cimoliopterus cuvieri*]. *Camposipterus(?) colorhinus* can be excluded from *Cimoliopterus cuvieri*, which lacks an anterior expansion of the rostrum or a depression above the first pair of alveoli.

*Camposipterus(?) colorhinus* shares with *Camposipterus nasutus* and *Camposipterus(?) sedgwickii* an anterior expansion of the rostrum and a round profile, and thus is tentatively referred to *Camposipterus*. As the syntypes are incomplete, it is uncertain if this taxon had a crest. It further shares with *Camposipterus(?) sedgwickii* the presence of a tall rostrum and robust anterior alveoli. However, *Camposipterus(?) colorhinus* represents a much larger and more robust pterosaur in comparison with the latter species.

### Pteranodontoidea *incertae sedis*

#### Cimoliopterus gen. n.

urn:lsid:zoobank.org:act:49BC7017-CEFC-4C53-8B14-61A9C40DF100

http://species-id.net/wiki/Cimoliopterus

##### Etymology.

From the Greek *kimolia*, chalk, and *pteron*, wing.

##### Type species.

*Pterodactylus cuvieri* Bowerbank, 1851.

##### Included species.

*Cimoliopterus cuvieri*.

##### Recorded temporal range.

Cenomanian / Turonian.

##### Recorded stratigraphic range.

Chalk Formation, England.

##### Diagnosis.

As for the type–species.

#### Cimoliopterus cuvieri

(Bowerbank, 1851) comb. n.

http://species-id.net/wiki/Cimoliopterus_cuvieri

Fig. 11

Pterodactylus cuvieri Bowerbank: Bowerbank 1851: p. 15, pl.IV
Pterodactylus cuvieri Bowerbank: Owen 1851b: p. 29
Pterodactylus cuvieri Bowerbank: Owen 1851a: p. 88, pl. XXVIII, fig. 1–7
“Ptenodactylus” cuvieri (Bowerbank): Seeley 1869: p. xvi [disclaimed]
Ornithocheirus cuvieri (Bowerbank): Seeley 1870: p. 113
Coloborhynchus cuvieri (Bowerbank): Owen 1874: p. 6
Ornithochirus [sic] cuvieri (Bowerbank): Lydekker 1888: p. 12
Ornithocheirus cuvieri (Bowerbank): Hooley 1914: p. 535
Ornithocheirus cuvieri (Bowerbank): Arthaber 1922: p. 16, fig. 6
Ornithocheirus cuvieri (Bowerbank): Wellnhofer 1978: p. 56, fig. 28
Anhanguera cuvieri (Bowerbank): Unwin 2001: p. 208, table 1

##### Holotype.

**Figure 11.**
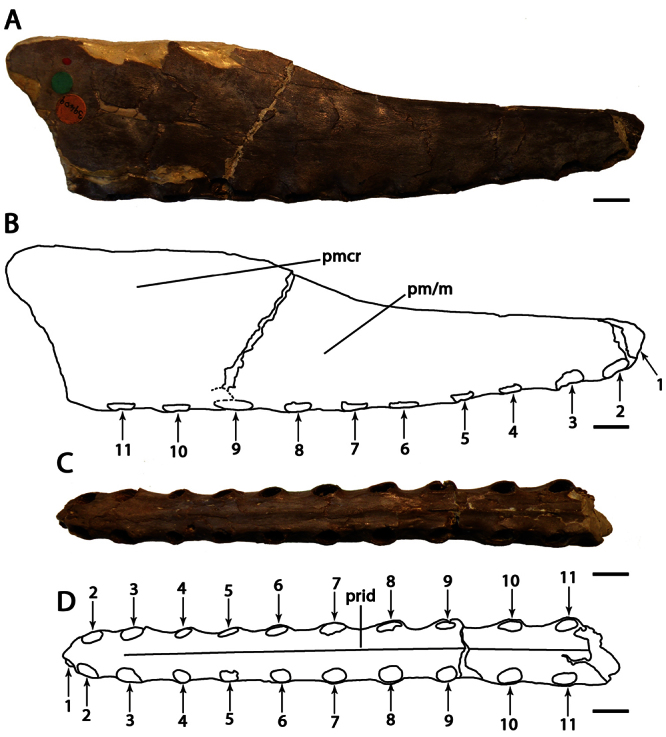
*Cimoliopterus cuvieri*. Holotype NHMUK PV 39409 (Cenomanian / Turonian, Chalk Formation), anterior part of the rostrum **A** right lateral view **B** respective line drawing **C** ventral view **D** respective line drawing. Abbreviations: **m** – maxillae, **pm** – premaxillae, **pmcr** – premaxillary crest, **prid** – palatal ridge. Arrows and numbers indicate alveoli or teeth and their respective position. Scale bar = 10 mm. Photos courtesy of The Natural History Museum.

NHMUK PV 39409, anterior portion of the rostrum (Fig. 11A–D).

##### Type locality.

Burham, Kent, England.

##### Type horizon.

Chalk Formation (Cenomanian / Turonian).

##### Diagnosis.

Pterodactyloid pterosaur with the following combination of characters that distinguishes it from other members of the clade (autapomorphies are marked with an asterisk): premaxillary crest present; premaxillary crest begins posteriorly (at the seventh pair of alveoli) but before the nasoantorbital fenestra*; palatal ridge extending anteriorly up to the third pair of alveoli; second and third alveoli similar in size and larger than the fourth; spacing between alveoli irregular, with the anterior alveoli more closely spaced and the posterior ones more widely separated from each other; almost 3 alveoli per 3 cm of jaw margin anteriorly and 2 alveoli each 3 cm posteriorly*; anterior expansion absent; palate dorsally curved.

##### Description.

Bowerbank (1851) described *Pterodactylus cuvieri* based on the holotype NHMUK PV 39409, which was recovered from the same pit in Burham as the holotype of *Lonchodraco giganteus*. NHMUK PV 39409 comprises a partial upper jaw. It is narrow in the preserved portion, without an anterior expansion of the rostrum, and presents a premaxillary crest which begins opposite to the seventh pair of alveoli (Bowerbank 1851). Bowerbank (1851) pointed out that the first pair of alveoli is located anteriorly, with the teeth projecting somewhat forwards, and that the spacing between the alveoli is about 1.5 times their diameter, the alveoli being irregularly placed and nearly equidistant. However, the spacing varies, with the first three pairs of alveoli more closely spaced.

NHMUK PV 39409 was originally reported as having a tooth preserved in the first right alveolus. During examination of the holotype in 2007 and 2009, the tooth was no longer preserved with the holotype and could not be found.

*Cimoliopterus cuvieri* differs from *Coloborhynchus clavirostris* in the lack of an anteriorly flat rostrum, premaxillary crest at the tip of the rostrum, anterior expansion, or the other diagnostic characters of that species (Rodrigues and Kellner 2008). In light of the identification of *Ornithocheirus simus* as type species of *Ornithocheirus*, *Cimoliopterus cuvieri* can be excluded from this genus by the possession of a low rostrum and the first pair of alveoli facing forwards. It can also be excluded from *Anhanguera* because it does not possess an anterior expansion of the rostrum (diagnostic for Anhangueridae) nor the fourth and fifth alveoli smaller than the third and sixth (diagnostic for *Anhanguera*). Furthermore, anhanguerids have a premaxillary crest that begins at or near the tip of the rostrum. The more posterior position of the crest in *Cimoliopterus cuvieri* may indicate that these crests evolved separately. *Anhanguera* is so far definitely known only from the Romualdo Formation of Brazil (e.g., Kellner and Tomida 2000), which is Albian in age (Pons et al. 1990). A few dozen anhanguerid crania are known, none of which has a posteriorly located premaxillary crest. Therefore, we place *Cimoliopterus cuvieri* in a new, currently monospecific genus.

#### ‘Ornithocheirus’ polyodon

Seeley, 1870

Fig. 12A–D

“Ptenodactylus” polyodon Seeley: Seeley 1869: p. xvi [disclaimed]
Ornithocheirus polyodon Seeley: Seeley 1870: p. 121
Ornithocheirus polyodon Seeley: Hooley 1914: p. 535
Anhanguera fittoni (Owen): Unwin 2001: table 1 [synonymy]

##### Holotype.

**Figure 12.**
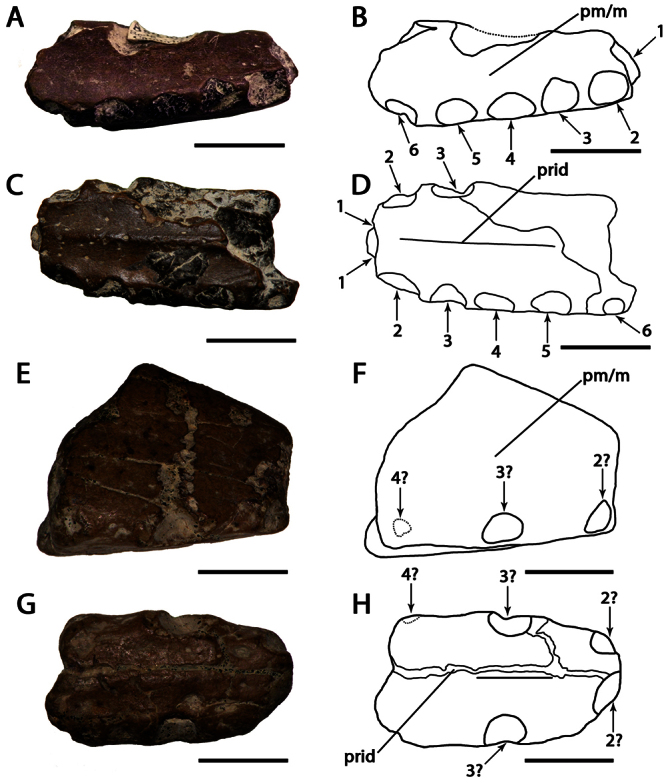
**A–D** *‘Ornithocheirus’ polyodon*, holotype CAMSM B54440 (Albian, Cambridge Greensand), anterior part of the rostrum. **A** right lateral view **B** respective line drawing **C** ventral view **D** respective line drawing. **E–H** *‘Ornithocheirus’ platystomus*, holotype CAMSM B54835 (Albian, Cambridge Greensand), anterior part of the rostrum **E** right lateral view **F** respective line drawing **G** ventral view **H** respective line drawing. Abbreviations: **m** – maxillae, **pm** – premaxillae, **prid** – palatal ridge. Arrows and numbers indicate alveoli or teeth and their respective position. Scale bar = 10 mm.

CAMSM B54440, anterior portion of the rostrum (Fig. 12A–D).

##### Type locality.

Cambridge, Cambridgeshire, England.

##### Type horizon.

Cambridge Greensand (Cenomanian; fossils Albian in age).

##### Diagnosis.

Pterodactyloid pterosaur with the following combination of characters that distinguishes it from other members of the clade (autapomorphies are marked with an asterisk): premaxillary crest absent; anterior expansion absent; palate dorsally curved; moderately developed palatal ridge; palate between the elevated alveolar rims and the palatal ridge concave; palatal ridge extending anteriorly up to the second pair of alveoli; alveoli ventrolaterally oriented; spacing between alveoli less than their diameters; approximately 5.5 alveoli per 3 cm of jaw margin*.

##### Description.

Seeley (1870) founded *‘Ornithocheirus’ polyodon* upon a fragmentary rostrum. He pointed out that CAMSM B54440 possesses a moderate palatal ridge, dorsally curved palate, first pair of alveoli facing forwards, more posterior alveoli facing more laterally than ventrally, and small spacing between the alveoli. He cited the last feature as the main difference between *‘Ornithocheirus’ polyodon* and *Pterodactylus fittoni*, thus justifying the creation of a new species.

*‘Ornithocheirus’ polyodon* can be excluded from *Ornithocheirus* because it lacks a tall rostrum. It cannot be referred to *Cimoliopterus* because the alveolar spacing is quite distinct; in *Cimoliopterus cuvieri*, only the first three pairs of alveoli are more closely positioned. It can also be excluded from *Camposipterus* because it lacks an anterior expansion of the rostrum.

*‘Ornithocheirus’ polyodon* shares with Lonchodraconidae the presence of small alveoli at the tip of the rostrum, which do not vary significantly in size. However, it differs from members of this clade in lacking an elevated alveolar margin or a prominent palatal ridge, as in *Lonchodraco(?) microdon*. However, it is possible that such elevation would get deeper posteriorly, but this cannot be confirmed in the holotype. Furthermore, the spacing between the alveoli being smaller than their diameters is not present in other lonchodraconids.

It is suggested here that *‘Ornithocheirus’ polyodon* might represent a new genus. As the known material is quite incomplete, we refrain from naming this taxon at the present time and use its originally proposed binomen.

### Pterodactyloidea *incertae sedis*

#### ‘Ornithocheirus’ platystomus

Seeley, 1870

Fig. 12E–H

“Ptenodactylus” platystomus Seeley: Seeley 1869: p. xvi [disclaimed]
Ornithocheirus platystomus Seeley: Seeley 1870: p. 120
Amblydectes platystomus (Seeley): Hooley 1914: p. 536
Criorhynchidae *incertae sedis*:Wellnhofer 1978: 60
Lonchodectes platystomus (Seeley): Unwin 2001: p. 211, table 1

##### Holotype.

CAMSM B54835, anterior portion of the rostrum (Fig. 12E–H).

##### Type locality.

Horningsea, Cambridgeshire, England.

##### Type horizon.

Cambridge Greensand (Cenomanian; fossils Albian in age).

##### Diagnosis.

Pterodactyloid pterosaur with the following combination of characters that distinguishes it from other members of the clade (autapomorphies are marked with an asterisk): alveoli small (about 4 to 5 mm in diameter); dorsal margin of the rostrum forms an angle of 27° with the ventral margin*.

##### Description.

*‘Ornithocheirus’ platystomus* is known from a partial premaxilla and maxillae. As Seeley (1870) pointed out, it is broken in its anterior tip, so that the first pair of alveoli is apparently not preserved. *‘Ornithocheirus’ platystomus* can be excluded from Lonchodraconidae because it does not have raised alveolar rims and lacks a deep palatal ridge. The absence of these characters results in the lack of a parapet–like palate (which distinguishes Lonchodectidae *sensu* Unwin 2001). *‘Ornithocheirus’ platystomus* presents a relatively tall rostrum, whose dorsal margin forms an angle with the ventral one; this angle lies between those in *Ornithocheirus simus* and *Cimoliopterus cuvieri*. The angle (about 27°) is so far unique among the species of the *Ornithocheirus* complex and confirms it as a valid taxon.

#### ‘Pterodactylus’ daviesii

Owen, 1874

Fig. 13A–D

Pterodactylus daviesii Owen: Owen 1874: p. 2, pl. I, fig. 5–6
Lonchodectes daviesii (Owen): Hooley 1914: p. 538
Ornithocheirus daviesii (Owen): Arthaber 1922: p. 16
Ornithochirus [sic] daviesi [sic] (Owen): Lydekker 1888: p. 23
Ornithocheirus daviesi [sic] (Owen): Wellnhofer 1978: p. 56, fig. 28
Lonchodectes platystomus (Seeley): Unwin 2001: fig. 12C

##### Holotype.

**Figure 13.**
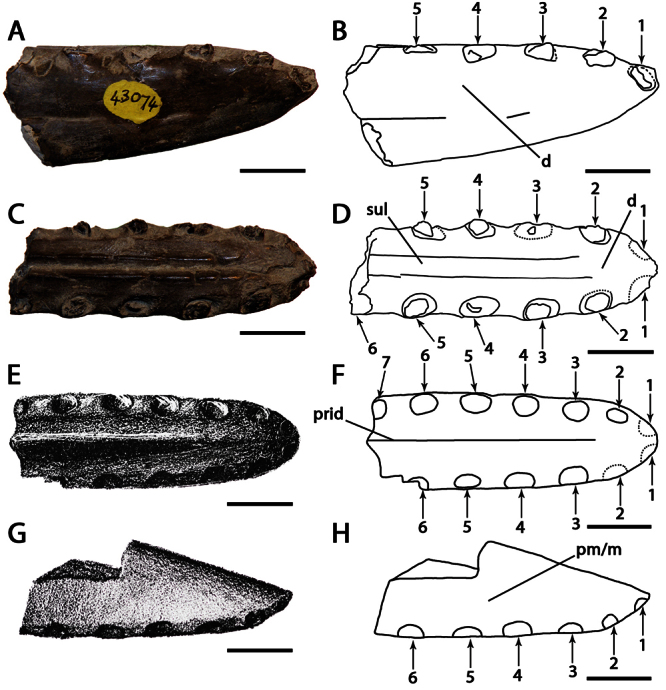
**A–D** *‘Pterodactylus’ daviesii*, holotype NHMUK PV 43074 (Albian, Gault Clay Formation), anterior part of the mandibular symphysis. **A** right lateral view **B** respective line drawing **C** dorsal view **D** respective line drawing. **E–H** *‘Ornithocheirus’ denticulatus*, holotype ?CAMSM B 54794 (Albian, Cambridge Greensand), anterior part of the rostrum **E** ventral view **F** respective line drawing **G** right lateral view **H** respective line drawing. Abbreviations: **m** – maxillae, **pm** – premaxillae, **prid**– palatal ridge, **sul** – sulcus. Arrows and numbers indicate alveoli or teeth and their respective position. Scale bar = 10 mm. **E** and **G** from Seeley 1870. Photos **A** and **C** courtesy of The Natural History Museum.

NHMUK PV 43074, partial mandibular symphysis (Fig. 13A–D).

##### Type locality.

Folkestone, Kent, England.

##### Type horizon.

Gault Clay Formation (Albian).

##### Diagnosis.

Pterodactyloid pterosaur with the following combination of characters that distinguishes it from other members of the clade (autapomorphies are marked with an asterisk): anterior expansion absent; mandibular crest absent; mandibular groove about 2.5 cm wide; mandibular groove with elevated margins; mandibular groove extends until the second pair of alveoli; alveoli of the anterior portion of the mandible without significant variation in size; alveoli equally spaced; first pair of teeth face anterodorsally; transverse section ‘V–shaped’; approximately 4 alveoli per 3 cm of jaw margin*.

##### Description.

*‘Pterodactylus’ daviesii* is known from a partial mandibular symphysis from the Gault Clay Formation. In his description, Owen (1874) stressed the presence of a mandibular sulcus and that the first pair of alveoli is less elliptical than the remaining ones and directed forwards and upwards. *‘Pterodactylus’ daviesii* presents a unique mosaic of seemingly plesiomorphic features among pterodactyloids. It lacks an anterior expansion, a crest, or any other distinctive dental feature, which would permit referral to a known genus. Instead, it is the combination of these characters that makes this species distinct from all known pterodactyloid genera.

It shares with Lonchodraconidae the presence of alveoli of the anterior portion of the mandible without significant variation in size, but can be confidently excluded from this clade because its alveoli are slightly larger and are not located on elevated alveolar margins. Although the dentary sulcus is relatively wide, it is narrower than in *Lonchodraco machaerorhynchus*. *‘Pterodactylus’ daviesii* is also distinct from *Lonchodraco giganteus*. In the latter, the mandible is very wide, with divergent margins, and rounded anteriorly. Although not comparable to *Ornithocheirus simus*, it is unlikely that it represents this much larger pterosaur.

##### Remarks.

Unwin (2001) did not cite *‘Pterodactylus’ daviesii* in the text of his review, but illustrated the holotype, referring to it as *Lonchodectes* [=*‘Ornithocheirus’*] *platystomus*, and indicated that it bears a crest. We disagree that NHMUK PV 43074 represents a crested pterosaur; the ventral margin of the dentary is sharp but there is no evidence for a crest. Furthermore, *‘Pterodactylus’ daviesii* is not directly comparable to *‘Ornithocheirus’ platystomus* because the latter is known only from an upper jaw.

*‘Pterodactylus’ daviesii* thus possibly represents a distinct taxon from the Gault Clay Formation. However, we refrain from naming it until more complete material becomes available and refer it using its original designation in single quotation marks.

#### ‘Ornithocheirus’ denticulatus

Seeley, 1870

Fig. 13E–H

Ornithocheirus denticulatus Seeley: Seeley 1870: p. 122, pl. XII, fig. 8–9
Ornithocheirus denticulatus Seeley:Hooley 1914: p. 535
Anhanguera cuvieri (Bowerbank): Unwin 2001: table 1

##### Holotype.

Anterior portion of the rostrum (?CAMSM B 54794) (Fig. 13E–H)

##### Type locality.

Cambridge, Cambridgeshire, England.

##### Type horizon.

Cambridge Greensand (Cenomanian; fossils Albian in age).

##### Diagnosis.

Pterodactyloid pterosaur with the following combination of characters that distinguishes it from other members of the clade: anterior expansion absent; palate dorsally curved; moderate palatal ridge; palatal ridge extending anteriorly up to the third pair of alveoli; spacing between alveoli approximately equal to their diameters; approximately 4.5 alveoli per 3 cm of jaw margin.

##### Description.

In the original description, Seeley (1870) contrasted this species with *‘Ornithocheirus’ polyodon* (see below). *‘Ornithocheirus’ denticulatus* shares with the latter the absence of an anterior expansion, the dorsally curved palate, and moderate size of the palatal ridge. However, in *‘Ornithocheirus’ denticulatus* the palatal ridge extends up to the third pair of alveoli, the spacing between the alveoli is larger, and the alveolar density is lower (4.5 alveoli per 3 cm). Because the dorsal margin of the premaxillae is broken, it cannot be access if *‘Ornithocheirus’ denticulatus* had a crest.

*‘Ornithocheirus’ denticulatus* does not share the combination of characters present in *Ornithocheirus*, *Lonchodraco*, *Cimoliopterus* and *Camposipterus* and thus cannot be referred to any of these genera. In particular, *‘Ornithocheirus’ denticulatus* lacks the alveolar pattern present in *Cimoliopterus cuvieri*. *‘Ornithocheirus’ denticulatus* somewhat resembles *‘Pterodactylus’ daviesii* in that both lack an anterior expansion of the jaw, sagittal crests, and have alveoli without significant variation in size and equally spaced. They differ in the alveolar density, which is slightly higher in *‘Ornithocheirus’ denticulatus*. Unfortunately, the known material of both species is not directly comparable and their taxonomic identity cannot be confirmed.

##### Remarks.

Collection data provided by the curators at the Sedgwick Museum of Earth Sciences in Cambridge list the specimen CAMSM B 54794 as the holotype of *‘Ornithocheirus’ denticulatus*. However, the specimen could not be found during review of the collection in October 2009. The specimen was, however, one of the few figured by Seeley (1870: pl. XII, figs 8 and 9).

In the lack of more complete material, we here refrain from naming a new genus based on the present specimen, and refer to it using the binomen in which it was originally proposed.

### Taxa considered *nomina dubia*

#### Palaeornis cliftii

Mantell, 1844 (nomen dubium)

Palaeornis cliftii Mantell: Mantell 1844: p. 806, fig. 149
Palaeornis cliftii Pterosaur bones: Owen 1846b: fig. 1–4
Pterodactylus sylvestris Owen: Owen 1859: p. 15
Pterodactylus ornis Owen: Owen 1861: p. 17
Ornithochirus [sic](?) clifti [sic] (Mantell): Lydekker 1888: p. 25
Ornithocheirus clifti (Mantell): Hooley 1914: p. 539
Ornithocheiridae *incertae sedis*: Wellnhofer 1978: 58
Ornithostoma sedgwicki (Seeley): Averianov 2011: p. 46

##### Holotype.

NHMUK PV 2353 and 2353a, partial left humerus.

##### Type locality.

Cuckfield, Sussex, England.

##### Type horizon.

Upper Tunbridge Wells Sand Formation (Witton et al. 2009), Hastings Group.

##### Remarks.

This species was first described as a bird (Mantell 1837, 1844) and later identified as a pterosaur bone (Owen 1846b). Newton (1888) pointed out that *Palaeornis cliftii* was a *nomen dubium*, and we concur. Witton et al. (2009) have recently thoroughly reviewed the taxonomic history and provided a detailed morphological description of *Palaeornis cliftii*, also considering it a *nomen dubium*. They also analyzed its phylogenetic affinities and pointed out that its classification as *Ornithocheirus* is problematic in part because the specimen cannot be compared with the holotype of *Ornithocheirus [simus]*. They identified the humerus as pertaining to Lonchodectidae (*sensu* Unwin 2001); however, as in the case with *Ornithocheirus simus*, no species referred in *Lonchodectes* (*sensu* Unwin 2001) or *Yixianopterus jingangshanensis* (Lü et al. 2006) has a known humerus, despite their illustration of a humerus allegedly pertaining to *Lonchodectes*. Subsequently, Averianov (2011) interpreted this specimen, along with some material from the Cambridge Greensand, as an azhdarchoid, more specifically *Ornithostoma*, which is known only from cranial material. As *Palaeornis cliftii* is funded upon non–diagnostic material, we consider it a *nomen dubium* and Azhdarchoidea indet.

#### Osteornis diomedeus

Gervais, 1844 (nomen dubium)

Osteornis diomedeus Gervais: Gervais 1844: p. 38
Cimoliornis diomedeus (Gervais): Owen 1846a: p. 545, fig. 230.
Ornithochirus [sic] diomedius [sic] (Gervais): Lydekker 1888: p. 13
Ornithocheirus diomedius [sic] (Gervais): Hooley 1914: p. 539
Ornithocheiridae *incertae sedis*: Wellnhofer 1978: 58

##### Holotype.

NHMUK PV 39418, distal end of a wing metacarpal.

##### Type locality.

Chesterton, Cambridgeshire, England.

##### Type horizon.

Chalk Formation (Cenomanian / Turonian).

##### Remarks.

Owen (1842) briefly described and figured a specimen (NHMUK PV 39418) that he considered to be the distal end of a tibia, belonging to a bird similar to an albatross, but he did not name it (Owen 1842: fig. 2). Gervais (1844) agreed with this identification and named it *Osteornis diomedeus*. Latter, Owen (1846a) remarked that Gervais used the name *Osteornis* not in the sense of a genus, but as a name he applied it to all fossil bird bones, and redesignated NHMUK PV 39418 as the type of *Cimoliornis diomedeus*. The specimen is now considered a fragment of the distal part of the wing metacarpal of a pterosaur (Owen 1859, 1874; Newton 1888; Wellnhofer 1978; Martill 2010). Hooley (1914) placed it provisionally in *Ornithocheirus*, while Wellnhofer (1978) referred it to Ornithocheiridae *incertae sedis*. Several authors (Hooley 1914; Lydekker 1888; Wellnhofer 1978; Martill 2010) misspelled the specific epithet as *diomedius*.

The two known pterosaur clades from the Chalk Formation are the Lonchodraconidae and *Cimoliopterus cuvieri*, whose metacarpals are unknown. This material is quite fragmentary, and its structure does not allow species or genus–level identification. Therefore, *Cimoliornis diomedeus* is considered a *nomen dubium*.

#### Pterodactylus compressirostris

Owen, 1851 (nomen dubium)

Fig. 14

Pterodactylus compressirostris Owen: Owen 1851b: p. 32, pl. V, figs 1–3. Owen 1851a: p. 95, pl. XXVIII, figs 8–10.
Ornithochirus [sic] compressirostris (Owen): Lydekker 1888: p. 11.
Ornithocheirus compressirostris (Owen): Arthaber 1922: p. 16, fig. 5.
Lonchodectes compressirostris (Owen): Hooley 1914: p. 535.
Ornthocheirus [sic] compressirostris (Owen): Kuhn 1967: p. 42.
Ornithocheirus compressirostris (Owen): Wellnhofer 1978: p. 56, fig. 4.
Lonchodectes compressirostris (Owen): Unwin 2001: p. 210, table 1.

##### Syntype.

**Figure 14.**
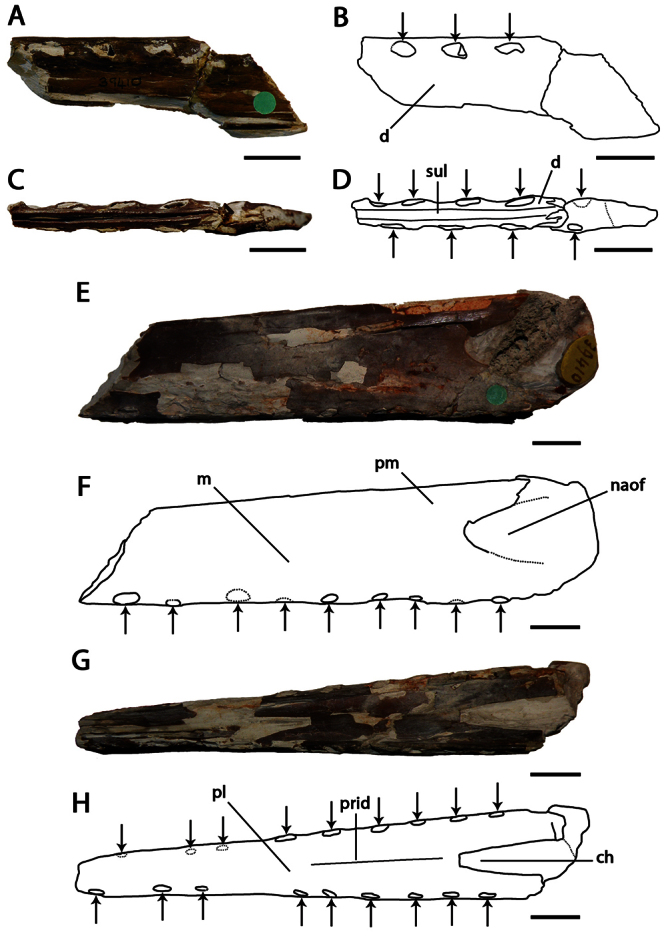
*Pterodactylus compressirostris*, holotype NHMUK PV 39410 (Cenomanian / Turonian, Chalk Formation). **A–D** proposed lectotype, fragment of the mandibular symphysis **A** left lateral view **B** respective line drawing **C** dorsal view **D** respective line drawing. **E–H** referred specimen, portion of the rostrum **E** left lateral view **F** respective line drawing **G** ventral view **H** respective line drawing. Abbreviations: **ch** – choanae, **d** – dentary, **m** – maxillae, **naof** – nasoantorbital fenestra, **pl** – palatine, **pm** – premaxillae, **prid** – palatal ridge, **sul**– sulcus. Arrows indicate alveoli or teeth. Scale bar = 10 mm. Photos courtesy of The Natural History Museum.

NHMUK PV 39410, partial rostrum and mandible (Fig. 14A–H).

##### Type locality.

Burham, Kent, England.

##### Type horizon.

Chalk Formation (Cenomanian / Turonian).

##### Description.

The holotype of *Pterodactylus compressirostris* consists of the middle portion of the rostrum (Fig. 14E–H), without the anterior end of the rostrum, and by a mandibular fragment that is strongly compressed mediolaterally (Fig. 14A–D). The rostrum has been subject to some distortion. The symphyseal fragment has been considered part of the upper jaw since its original description (Owen 1851b), until Kellner (1990: 100) demonstrated the presence of a medial groove and reidentified it as a lower jaw. Owen (1851a) referred both specimens to the same species, because they come “from the same pit, if not from the same block”. It is unclear whether these specimens were found associated, and they could well represent different individuals (even if conspecific). It is noteworthy that at least two different pterosaur species from the Chalk Formation were found in the same pit (*Cimoliopterus cuvieri* and *Lonchodraco giganteus*, according to Bowerbank 1852). Kellner (1990) suggested that these two fragments might belong to different individuals, so that (1) they should receive separate catalog numbers, and (2) one of them should be designated the lectotype. As the majority of species from the *Ornithocheirus* complex is based on jaw tips, we here designate the mandibular fragment as the lectotype, with the original catalogue number NHMUK PV 39410. The other specimen, comprising the middle part of the rostrum, should be renumbered.

The lectotype of *Pterodactylus compressirostris* does not have a mandibular crest or raised alveoli, excluding it from Lonchodraconidae. It is distinctly compressed, incomplete, and non–diagnostic. Regarding the the cranial portion, few comparisons to *Lonchodraco(?) microdon* are possible as the specimens are not directly comparable, but they differ in the depth of the palatal ridge, which is lower in *Pterodactylus compressirostris*. The cranial fragment has small and widely spaced alveoli, reminiscent of Lonchodraconidae, but it is not possible to evaluate whether this feature extended to the tips of the jaws, as in *Lonchodraco giganteus*, *Lonchodraco machaerorhynchus*, and *Lonchodraco(?) microdon*, or if the anteriormost alveoli showed size variation, as in Anhangueridae. The referred specimen shares with Anhangueridae small alveoli on raised alveolar margins in the posterior portion of the maxillae, with the spacing between them roughly equivalent to their diameters. It differs from Anhangueridae in the lack of a premaxillary crest. However, crucial information is lacking due to the absence of the anterior portion of the rostrum in this specimen, a portion very diagnostic for toothed pteranodontoids, and upon which the taxonomy of the group is largely based. Both specimens upon which the species is based are uninformative. Therefore, we here consider *Pterodactylus compressirostris* a *nomen dubium*.

##### Remarks.

*Pterodactylus compressirostris* was until recently involved in a taxonomic problem. Khozatskii and Yur’ev (1964) and Kuhn (1967) erroneously considered it the type species of *Ornithocheirus*. This proposal was adopted by latter researchers (Wellnhofer 1978; Kellner and Tomida 2000; Veldmeijer et al. 2009). Unwin (2001), however, demonstrated that the type species of *Ornithocheirus* should be *Ornithocheirus simus* by monotypy.

To further complicate the taxonomy of this species, Kuhn (1967) also referred *Pterodactylus compressirostris* as the type species of *Lonchodectes*, and considered *Lonchodectes* synonymous with *Ornithocheirus*. Because we consider *Pterodactylus compressirostris* a *nomen dubium*, the genus *Lonchodectes* Hooley, 1914 and the family Lonchodectidae Unwin, 2001 should not be used.

#### Pterodactylus fittoni

Owen, 1859 (nomen dubium)

Fig. 15A–D

Pterodactylus fittoni Owen: Owen 1859: p. 4, pl. I, fig. 3
“Ptenodactylus” fittoni (Owen): Seeley 1869: p. xvi [disclaimed]
Ornithocheirus fittoni (Owen): Seeley 1870: p. 118
Ornithochirus [sic] fittoni (Owen): Lydekker 1888: p. 15
Ornithocheirus fittoni (Owen): Hooley 1914: p. 535
Ornithocheirus fittoni (Owen): Arthaber 1922: p. 17
Ornithocheirus fittoni (Owen): Wellnhofer 1978: p. 57
Anhanguera fittoni (Owen): Unwin 2001: p. 194, fig. 10F–G, table 1

##### Holotype.

**Figure 15.**
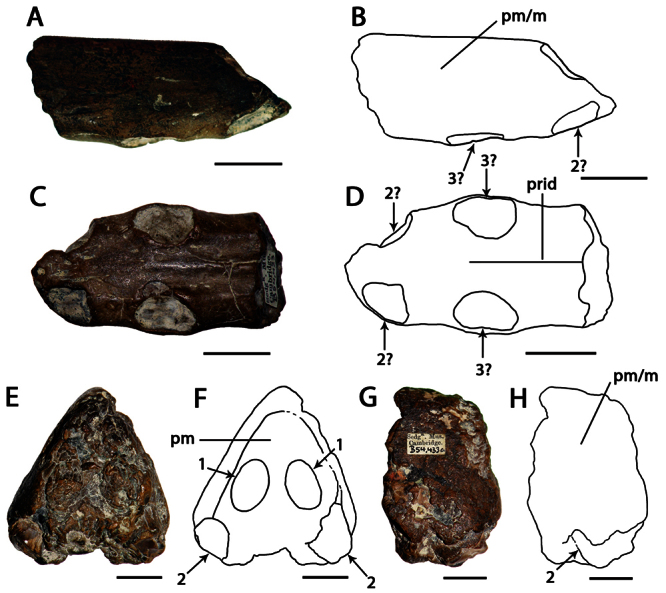
**A–D** *Pterodactylus fittoni*, holotype CAMSM B54423 (Albian, Cambridge Greensand), anterior part of the rostrum. **A** right lateral view **B** respective line drawing **C** ventral view **D** respective line drawing. **E–H** *Ornithocheirus woodwardi*, holotype CAMSM B 54433a (Albian, Cambridge Greensand), anterior part of the rostrum **E** anterior view **F** respective line drawing **G** right lateral view **H** respective line drawing. Abbreviations: **m** – maxillae, **pm** – premaxillae, **prid** – palatal ridge. Arrows and numbers indicate alveoli or teeth and their respective position. Scale bar = 10 mm.

CAMSM B54423, anterior portion of the rostrum (Fig. 15A–D).

##### Type locality.

Cambridge, Cambridgeshire, England.

##### Type horizon.

Cambridge Greensand (Cenomanian; fossils Albian in age).

##### Description.

CAMSM B54423 is a fragment of the anterior portion of the premaxillae and maxillae, lacking the anteriormost end. It is likely that the first pair of alveoli is not preserved (Unwin 2001, contra Owen 1859), as small and anteriorly located first pair of alveoli is common among Cambridge Greensand pterosaurs. This possibility becomes more likely when *Pterodactylus fittoni* is compared with *Ornithocheirus enchorhynchus*: both possess similar structure and size, but the latter has the first pair of alveoli preserved. CAMSM B54423 is low dorsoventrally and shows no signs of an anterior expansion or a premaxillary crest. However, these absences could be due to poor preservation as the specimen is fragmentary; for instance, the presence of a posteriorly located crest as in *Cimoliopterus cuvieri* cannot be ruled out. *Pterodactylus fittoni* shares with *Cimoliopterus cuvieri* a low tip of the snout, the absence of an anterior expansion, a dorsally curved palate and, if the first preserved pair of alveoli is actually the second pair, a palatal ridge extending back to the third pair of alveoli. CAMSM B54423 differs from *Cimoliopterus cuvieri* in the height of the rostrum and in that the tip of the rostrum is wider than high; this last feature could be due to the fracture of the tip. In conclusion, *Pterodactylus fittoni* cannot be excluded from *Cimoliopterus cuvieri* but it also cannot be definitely referred to that species and therefore it is considered here a *nomen dubium*.

#### Pterodactylus woodwardi

Owen, 1861 (nomen dubium)

Fig. 15E–H

Pterodactylus woodwardi Owen: Owen 1861: p. 4, pl. II, fig. 3
“Ptenodactylus” woodwardi (Owen): Seeley 1869: p. xvi [disclaimed]
Ornithocheirus woodwardi (Owen): Seeley 1870: p. 125
Ornithochirus [sic](?) simus (Owen): Lydekker 1888: p. 16
Criorhynchus woodwardi (Owen): Hooley 1914: p. 536
Criorhynchus woodwardi (Owen): Arthaber 1922: p. 18
Criorhynchus simus (Owen): Wellnhofer 1978: p. 60
Coloborhynchus sedgwickii (Owen): Unwin 2001: p. 194, table 1

##### Holotype.

CAMSM B 54433a, anterior portion of the rostrum (Fig. 15E–H).

##### Type locality.

Cambridge, Cambridgeshire, England.

##### Type horizon.

Cambridge Greensand (Cenomanian; fossils Albian in age).

##### Description.

The holotype of *Pterodactylus woodwardi* comprises a fragment of the anterior portion of the tip of the rostrum. It is quite incomplete, consisting mostly of a transverse section. The first pair of teeth is located anteriorly, and the second pair faces anteroventrally. There is no premaxillary crest at the anteriormost tip of the rostrum, but the presence of a more posteriorly located crest cannot be ruled out. The specimen is very fragmentary and several important characters cannot be observed on it. Therefore, it is considered a *nomen dubium*.

##### Remarks.

*Pterodactylus woodwardi* was listed as *Ornithocheirus woodwardi* by Seeley (1870). Lydekker (1888) also referred it to *Ornithocheirus*, but as *Ornithocheirus simus* (with the misspelling *Ornithochirus*). Wellnhofer (1978) considered it synonymous with *Criorhynchus* [=*Ornithocheirus*] *simus*. Hooley (1914) referred it as *Criorhynchus woodwardi*. Most recently, Unwin (2001) synonymized it with *Coloborhynchus* [=*Camposipterus(?)*] *sedgwickii*.

#### Ornithocheirus brachyrhinus

Seeley, 1870 (nomen dubium)

Fig. 16A–D

“Ptenodactylus” brachyrhinus Seeley: Seeley 1869: p. xvi [disclaimed]
Ornithocheirus brachyrhinus Seeley: Seeley 1870: p. 123
Ornithocheirus brachyrhinus Seeley: Hooley 1914: p. 535
Anhanguera cuvieri (Bowerbank): Unwin 2001: table 1

##### Holotype.

**Figure 16.**
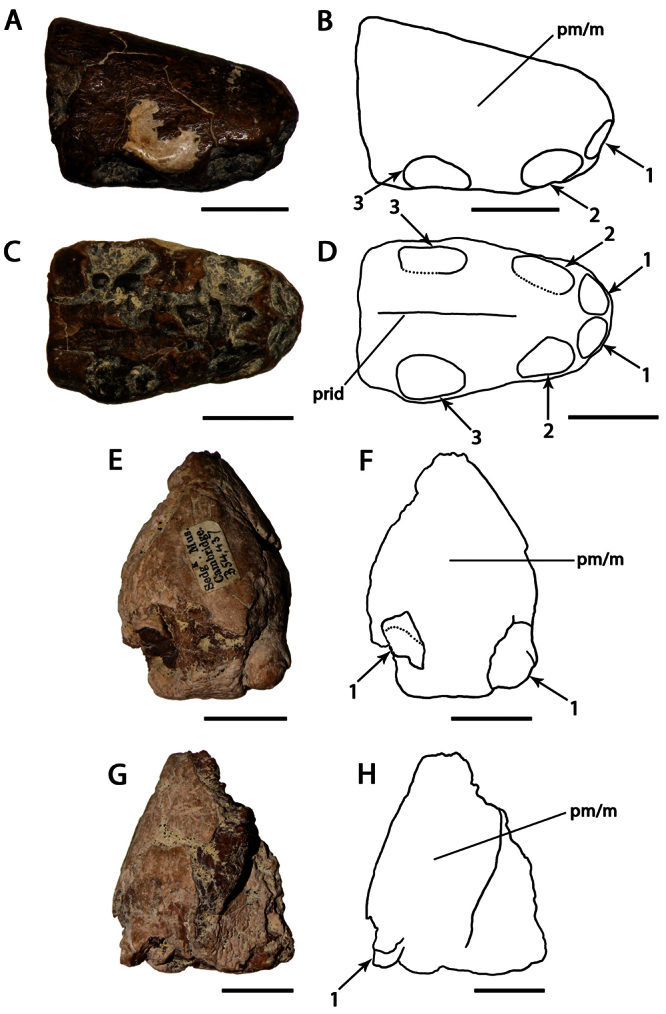
**A–D** *Ornithocheirus brachyrhinus*, holotype CAMSM B54443 (Albian, Cambridge Greensand), anterior part of the rostrum. **A** right lateral view **B** respective line drawing **C** ventral view **D** respective line drawing. **E–H** *Ornithocheirus carteri*, holotype CAMSM B 54437 (Albian, Cambridge Greensand), anterior part of the rostrum **E** anterior view **F** respective line drawing **G** left lateral view **H** respective line drawing. Abbreviations: **m** – maxillae, **pm** – premaxillae, **prid** – palatal ridge. Arrows and numbers indicate alveoli or teeth and their respective position. Scale bar = 10 mm.

CAMSM B54443, anterior portion of the rostrum (Fig. 16A–D).

##### Type locality.

Cambridge, Cambridgeshire, England.

##### Type horizon.

Cambridge Greensand (Cenomanian; fossils Albian in age).

##### Description.

*Ornithocheirus brachyrhinus* is known from the tip of a snout, with a dorsally curved palate and lacking an anteriorly located crest. It shares with *Cimoliopterus cuvieri* features such as the curved palate, the anterior end being higher than wide, lack of an anterior expansion, and absence of an anterior crest. The structure of *Ornithocheirus brachyrhinus* corresponds perfectly to the tip of the snout of *Cimoliopterus cuvieri* and it is possibly referable to that species, as Unwin (2001) has suggested. However, CAMSM B54443 is fragmentary and therefore it is not possible to establish if it had a posteriorly located crest and the alveoli size variation diagnostic of *Cimoliopterus cuvieri*. Thus, *Ornithocheirus brachyrhinus* is here considered a *nomen dubium*.

#### Ornithocheirus carteri

Seeley, 1870 (nomen dubium)

Fig. 16E–H

Ornithocheirus carteri Seeley: Seeley 1869: p. xvi [disclaimed]
Ornithocheirus carteri Seeley: Seeley 1870: p. 128
Criorhynchus carteri (Seeley): Hooley 1914: p. 536
Criorhynchus simus (Owen): Wellnhofer 1978: p. 60
Ornithocheirus simus (Owen): Unwin 2001: table 1

##### Holotype.

CAMSM B 54437, anterior portion of the rostrum (Fig. 16E–H).

##### Type locality.

Cambridge, Cambridgeshire, England.

##### Type horizon.

Cambridge Greensand (Cenomanian; fossils Albian in age).

##### Description.

Seeley (1870) named this species based on some differences between CAMSM B 54437 and the holotype of *Ornithocheirus simus*. He noted that the rostrum is not as high and narrower; the lateral surfaces bear several longitudinal furrows, which he believed to be impressions of blood vessels; and the first pair of teeth are larger, more conical, circular, and separated by a large gap. Although the rostrum is not as tall as in *Ornithocheirus simus*, it is not possible to determine if this size difference is merely ontogenetic. However, the separation between the right and left teeth of the first pair of alveoli is larger than the holotype of *Ornithocheirus simus*. Therefore, we reject referring *Ornithocheirus carteri* to *Ornithocheirus simus* and consider it a *nomen dubium*.

#### Ornithocheirus crassidens

Seeley, 1870 (nomen dubium)

Fig. 17A–D

“Ptenodactylus” crassidens Seeley: Seeley 1869: p. xvi [disclaimed]
Ornithocheirus crassidens Seeley: Seeley 1870: p. 122
Amblydectes crassidens (Seeley): Hooley 1914: p. 536
Criorhynchidae *incertae sedis*:Wellnhofer 1978: 60
Coloborhynchus sedgwickii (Owen): Unwin 2001: table 1

##### Holotype.

**Figure 17.**
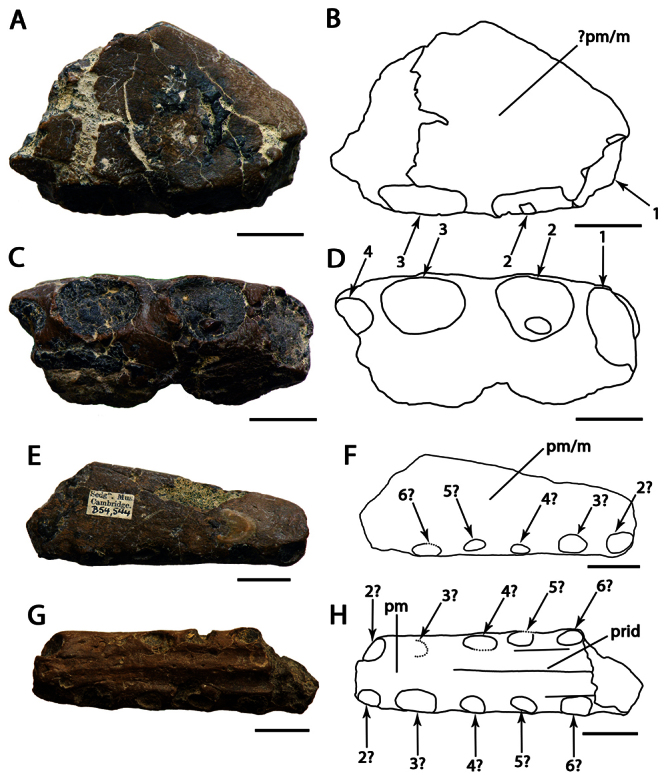
**A–D** *Ornithocheirus crassidens*, holotype CAMSM B 54499 (Albian, Cambridge Greensand), anterior fragment of the rostrum. **A** right lateral view **B** respective line drawing **C** ventral view **D** respective line drawing. **E–H** *Ornithocheirus dentatus*, holotype CAMSM B 54544 (Albian, Cambridge Greensand), anterior part of the rostrum **E** right lateral view **F** respective line drawing **G** ventral view **H** respective line drawing. Abbreviations:: **m** – maxillae, **pm** – premaxillae, **prid** – palatal ridge. Arrows and numbers indicate alveoli or teeth and their respective position. Scale bar = 10 mm.

CAMSM B 54499, anterior portion of a jaw (Fig. 17A–D).

##### Type locality.

Cambridge, Cambridgeshire, England.

##### Type horizon.

Cambridge Greensand (Cenomanian; fossils Albian in age).

Description: CAMSM B 54499 is a very fragmentary specimen, in which much of the left side, especially the oral surface, was not preserved. Seeley (1870) tentatively identified it as premaxillae. He compared it to *Camposipterus(?) colorhinus* and, among other differences, observed that *Ornithocheirus crassidens* lacks the depression above the first pair of alveoli diagnostic for *Camposipterus(?) colorhinus*, so Seeley (1870) considered it a new species. Based on its height the holotype seems to be an upper jaw, but neither a ridge nor a sulcus are evident and thus it is not possible to identify it with certainty. The species is considered here a *nomen dubium*.

##### Remarks.

The taxonomy of this species is controversial. It was placed in the genus *Ornithocheirus* by Seeley (1870). Hooley (1914) attributed *Ornithocheirus crassidens* to a new genus, *Amblydectes*, and Wellnhofer (1978) referred it as a Criorhynchidae *incertae sedis* and provisionally placed it in *Criorhynchus*. Most recently, Unwin (2001) considered it synonymous with *Coloborhynchus* [=*Camposipterus(?)*] *sedgwickii*.

#### Ornithocheirus dentatus

Seeley, 1870 (nomen dubium)

Fig. 17E–H

“Ptenodactylus” dentatus Seeley: Seeley 1869: p. xvi [disclaimed]
Ornithocheirus dentatus Seeley: Seeley 1870: p. 119
Ornithocheirus dentatus Seeley: Hooley 1914: p. 535
Anhanguera cuvieri (Bowerbank): Unwin 2001: table 1

##### Holotype.

CAMSM B 54544, anterior portion of the rostrum (Fig. 17E–H).

##### Type locality.

Cambridge, Cambridgeshire, England.

##### Type horizon.

Cambridge Greensand (Cenomanian; fossils Albian in age).

##### Description.

Seeley (1870) described *Ornithocheirus dentatus* based on a fragmentary anterior portion of an upper jaw. Interestingly, CAMSM B 54544 lacks a fully developed alveolus on the right side (possibly the third), but differing from *Camposipterus(?) sedgwickii* and *Anhanguera blittersdorffi* (Pz–DBAV UERJ 40) because its margins are slightly marked. In the other specimens, the margins are completely absent.

Seeley (1870) compared and distinguished *Ornithocheirus dentatus* from *Ornithocheirus* [=*Camposipterus(?)*] *sedgwickii* based on a wider palate and smaller teeth. It is hard to understand what he meant by wider palate, since CAMSM B 54544 is quite smaller individual in comparison to the holotype of *Camposipterus(?) sedgwickii*, but both species can be further distinguished by the lack of an anterior expansion and of a dorsally curved palate in *Ornithocheirus dentatus*, besides a lower rostrum in the latter.

Unwin (2001) synonymized *Ornithocheirus dentatus* with *Anhanguera* [=*Cimoliopterus*] *cuvieri*. However, Seeley (1870) had already noticed that both species can be differentiated by the presence of smaller alveoli, which are placed more closely together. It can also be excluded from the Lonchodraconidae as it does not possess a deep palatal ridge or alveoli placed on elevations. Due to fragmentary state of CAMSM B 54544, we regard *Ornithocheirus dentatus* as a *nomen dubium*.

#### Ornithocheirus enchorhynchus

Seeley, 1870 (nomen dubium)

Fig. 18A–D

“Ptenodactylus” enchorhynchus Seeley: Seeley 1869: p. xvi [disclaimed]
Ornithocheirus enchorhynchus Seeley: Seeley 1870: p. 123
Ornithocheirus enchorhynchus Seeley: Hooley 1914: p. 535
Anhanguera cuvieri (Bowerbank): Unwin 2001: table 1

##### Holotype.

**Figure 18.**
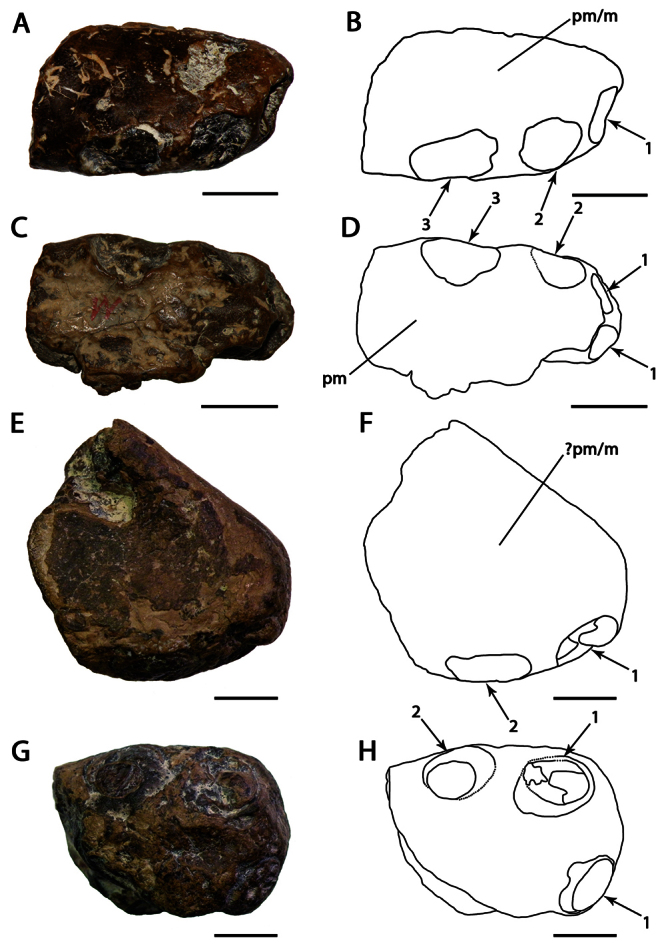
**A–D** *Ornithocheirus enchorhynchus*, holotype CAMSM B 54444 (Albian, Cambridge Greensand), anterior part of the rostrum. **A** right lateral view **B** respective line drawing **C** ventral view **D** respective line drawing. **E–H** *Ornithocheirus eurygnathus*, holotype CAMSM B54644 (Albian, Cambridge Greensand), anterior part of the rostrum **E** ?right lateral view **F** respective line drawing **G** ?ventral view **H** respective line drawing. Abbreviations: **m** – maxillae, **pm** – premaxillae. Arrows and numbers indicate alveoli or teeth and their respective position. Scale bar = 10 mm.

CAMSM B 54444, anterior portion of the rostrum (Fig. 18A–D).

##### Type locality.

Cambridge, Cambridgeshire, England.

##### Type horizon.

Cambridge Greensand (Cenomanian; fossils Albian in age).

##### Description.

CAMSM B 54444 is a fragment of the anterior portion of the premaxillae and maxillae, including three pairs of alveoli. The first pair of alveoli is located anteriorly, separated by a thin wall of bone from the second pair. The spacing between the second and third pairs is larger but still smaller than the diameter of the alveoli. Such spacing is common within species of the *Ornithocheirus* complex which share dorsal curvature of the palate, as is in the present specimen.

Seeley (1870) noted that it was similar to *Ornithocheirus brachyrhinus* but is larger, has a wider palate, lacks a palatal ridge, and has a larger first pair of alveoli. These size differences could be due to ontogeny, whereas the absence of a ridge could be explained by postmortem abrasion. Seeley (1870) compared it with *Camposipterus(?) colorhinus*, but pointed out that the latter has a diagnostic anterior depression, which is absent in CAMSM B 54444.

Unwin (2001) synonymized *Ornithocheirus enchorhynchus* with *Anhanguera* [=*Cimoliopterus*] *cuvieri*. Both species are quite similar, sharing features such as the absence of a crest on the anterior end of the rostrum, the dorsally curved palate, the first pair of teeth facing anteriorly, and the absence of an anterior expansion. However, as in the case of *Ornithocheirus brachyrhinus*, the incompleteness of the holotype of *Ornithocheirus enchorhynchus* precludes it from being compared to *Cimoliopterus cuvieri*. Therefore, *Ornithocheirus enchorhynchus* is considered here a *nomen dubium*.

##### Remarks.

Hooley (1914) agreed with Seeley (1870) that this species should be referred to *Ornithocheirus*, and Wellnhofer (1978) provisionally followed him. Unwin (2001) synonymized it with *Anhanguera* [=*Cimoliopterus*] *cuvieri*.

#### Ornithocheirus eurygnathus

Seeley, 1870 (nomen dubium)

Fig. 18E–H

“Ptenodactylus” eurygnathus Seeley: Seeley 1869: p. xvi [disclaimed]
Ornithocheirus eurygnathus Seeley: Seeley 1870: p. 123
Amblydectes eurygnathus (Seeley): Hooley 1914: p. 536
Criorhynchidae *incertae sedis*:Wellnhofer 1978: 60
Coloborhynchus capito (Seeley): Unwin 2001: table 1

##### Holotype.

CAMSM B54644, anterior fragment of an ?upper jaw (Fig. 18E–H).

##### Type locality.

Ditton, Cambridgeshire, England.

##### Type horizon.

Cambridge Greensand (Cenomanian; fossils Albian in age).

##### Description.

CAMSM B54644 was tentatively identified by Seeley (1870) as the tip of a dentary. It has very large alveoli and an oval depression between the first pair of alveoli; it also has a median crest beginning at the tip of the jaw. Upon examination, it could be observed that the crest is fairly high, so the fragment could be an upper jaw. However, the presence of neither a palatal ridge nor a dentary sulcus could be recognized in the specimen, so it is not possible to identify it as an upper jaw with certainty. Therefore, we consider the species a *nomen dubium*.

#### Ornithocheirus oxyrhinus

Seeley, 1870 (nomen dubium)

Fig. 19A–B

“Ptenodactylus” oxyrhinus Seeley: Seeley 1869: p. xvi [disclaimed]
Ornithocheirus oxyrhinus Seeley: Seeley 1870: p. 117
Ornithocheirus oxyrhinus Seeley: Hooley 1914: p. 535

##### Holotype.

**Figure 19.**
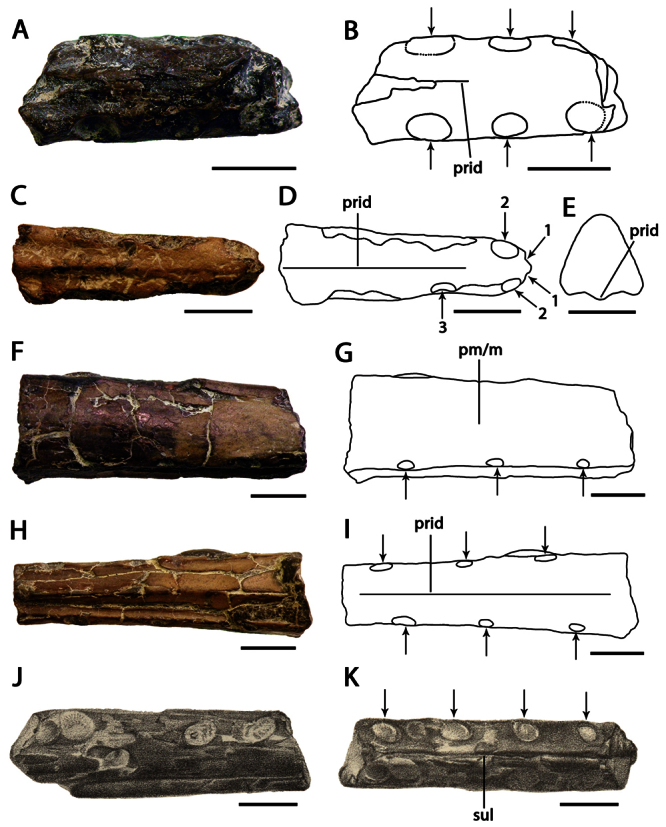
**A–B** *Ornithocheirus oxyrhinus*, holotype CAMSM B 54612 (Albian, Cambridge Greensand), anterior part of the rostrum. **A** ventral view **B** respective line drawing. **C–E** *Ornithocheirus scaphorhynchus*, holotype CAMSM B 54441 (Albian, Cambridge Greensand), anterior part of the rostrum **C** ventral view **D** respective line drawing **E** line drawing in posterior view. **F–I** *Ornithocheirus tenuirostris*, holotype CAMSM B 54584 (Albian, Cambridge Greensand), anterior part of the rostrum **F** right lateral view **G** respective line drawing **H** ventral view **I** respective line drawing. **J–K** *Ornithocheirus xyphorhynchus*, holotype (Albian, Cambridge Greensand), anterior part of the rostrum **J** lateral view **K** dorsal view. Abbreviations: **m** – maxillae, **pm** – premaxillae, **prid** – palatal ridge, **sul** – sulcus. Arrows and numbers indicate alveoli or teeth and their respective position. Scale bar = 10 mm. J and K from Seeley 1881.

CAMSM B 54612, anterior fragment of an upper jaw (Fig. 19A–B).

##### Type locality.

Smithswashing, Coton, Cambridgeshire, England.

##### Type horizon.

Cambridge Greensand (Cenomanian; fossils Albian in age).

##### Description.

*Ornithocheirus oxyrhinus* was described by Seeley (1870) based on CAMSM B 54612, a portion of an upper jaw, with a palatal ridge and some alveoli. However, the specimen is incomplete and lacks any features that justify recognition of a distinct species. We here regard it as a *nomen dubium*.

##### Remarks.

Unwin (2001) classified it as a *nomen nudum*; however, it has a proper description (like most of the new species described by Seeley in 1870) so it is not technically a *nomen nudum*.

#### Ornithocheirus scaphorhynchus

Seeley, 1870 (nomen dubium)

Fig. 19C–E

“Ptenodactylus” scaphorhynchus Seeley: Seeley 1869: p. xvi [disclaimed]
Ornithocheirus scaphorhynchus Seeley: Seeley 1870: p. 119
Lonchodectes scaphorhynchus (Seeley): Hooley 1914: p. 535
Anhanguera cuvieri (Bowerbank): Unwin 2001: table 1

##### Holotype.

CAMSM B 54441, anterior portion of the rostrum (Fig. 19C–E).

##### Type locality.

Cambridge, Cambridgeshire, England.

##### Type horizon.

Cambridge Greensand (Cenomanian; fossils Albian in age).

##### Description.

Seeley (1870) described *Ornithocheirus scaphorhynchus* based on fragmentary conjoined premaxillae and maxillae. The incompleteness of the specimen makes it difficult to refer it with certainty to any known genus. The alveolar margin is poorly preserved but it does not seem to fit the alveolar spacing pattern in the holotype of *Cimoliopterus cuvieri*. It has a somewhat raised alveolar margin but the palatal ridge is not deep, as diagnostic for *Lonchodraco*. Therefore, *Ornithocheirus scaphorhynchus* is considered here a *nomen dubium*.

#### Ornithocheirus tenuirostris

Seeley, 1870 (nomen dubium)

Fig. 19F–I

“Ptenodactylus” tenuirostris Seeley: Seeley 1869: p. xvi [disclaimed]
Ornithocheirus tenuirostris Seeley: Seeley 1870: p. 114
Lonchodectes tenuirostris (Seeley): Hooley 1914: p. 535
Lonchodectes compressirostris (Owen): Unwin 2001: fig. 11A–B, table 1

##### Holotype.

CAMSM B 54584, anterior portion of the rostrum (Fig. 19F–I).

##### Type locality.

Coton, Cambridgeshire, England.

##### Type horizon.

Cambridge Greensand (Cenomanian; fossils Albian in age).

##### Description.

*Ornithocheirus tenuirostris* was described by Seeley (1870). He noted that it is a fragment of an upper jaw, broken at both ends. The transverse section varies from elliptical in outline posteriorly to somewhat triangular anteriorly; the specimen has suffered some distortion. The alveoli are elliptical and well separated from each other. It was recently synonymized with *Lonchodectes* [=*Pterodactylus*] *compressirostris* by Unwin (2001), who stated that CAMSM B 54584 is identical to the holotype of the latter. Both holotypes, however, are not comparable. CAMSM B 54584 most probably comes from a portion more anteriorly located on the jaw than represented by the referred specimen of *Pterodactylus compressirostris*, and the holotype, previously interpreted as an upper jaw, is actually part of a mandibular symphysis (see above). Furthermore, *Ornithocheirus tenuirostris* can be excluded from Lonchodraconidae because the palatal ridge and the alveolar margins are not raised, as in the species referred to that clade. *Ornithocheirus tenuirostris* lacks any diagnostic features and is considered a *nomen dubium*.

#### Ornithocheirus xyphorhynchus

Seeley, 1870 (nomen dubium)

Fig. 19J–K

Ornithocheirus xyphorhynchus Seeley: Seeley 1870: p. 117
Ornithocheirus xyphorhynchus Seeley: Seeley 1881: p. 18, plate I, fig. 2
Ornithocheirus xyphorhynchus Seeley: Hooley 1914: p. 538
Ornithocheirus xyphorhynchus Seeley: Arthaber 1922: p. 17
Anhanguera cuvieri (Bowerbank): Unwin 2001: table 1

##### Holotype.

anterior portion of the rostrum (collection data could not be recovered) (Fig. 19J–K).

##### Type locality.

Cambridge, Cambridgeshire, England.

##### Type horizon.

Cambridge Greensand (Cenomanian; fossils Albian in age).

##### Description.

The holotype of *Ornithocheirus xyphorhynchus* was illustrated by Seeley (1881: plate I, Figs 2a and 2b), and, as the type material of *‘Ornithocheirus’ reedi*, belonged to the collection of W. Reed of York at the time when it was first described. It could not be found in the collections of the Natural History Museum, the Sedgwick Museum of Earth Sciences, or the Manchester Museum during visits in October 2009. Therefore, we base our remarks on the descriptions and illustrations provided by Seeley (1870, 1881).

The species was based on a fragmentary mandible, lacking the tip. It did not have a dentary crest (Seeley 1870). Unwin (2001) recently referred it to *Anhanguera* [=*Cimoliopterus*] *cuvieri*. However, the latter is known by a cranial material from the Chalk Formation, whereas *Ornithocheirus xyphorhynchus* is known only by a partial lower jaw from the Cambridge Greensand. As both species are known by material which is not directly comparable, the proposed synonymy can be rejected.

#### Pterodactylus sagittirostris

Owen, 1874 (nomen dubium)

Fig. 20

Pterodactylus sagittirostris Owen: Owen 1874: p. 3, pl. II, fig 1–8.
Lonchodectes sagittirostris (Owen): Hooley 1914: p. 538.
Ornithocheirus sagittirostris (Owen): Arthaber 1922: p. 16.
Ornithocheirus sagittirostris (Owen): Wellnhofer 1978.
Lonchodectes sagittirostris (Owen): Unwin 2001: 209.
Lonchodectes sagittirostris (Owen): Martill, Sweetman and Witton 2011: p. 385, fig. 25.12

##### Holotype.

**Figure 20.**
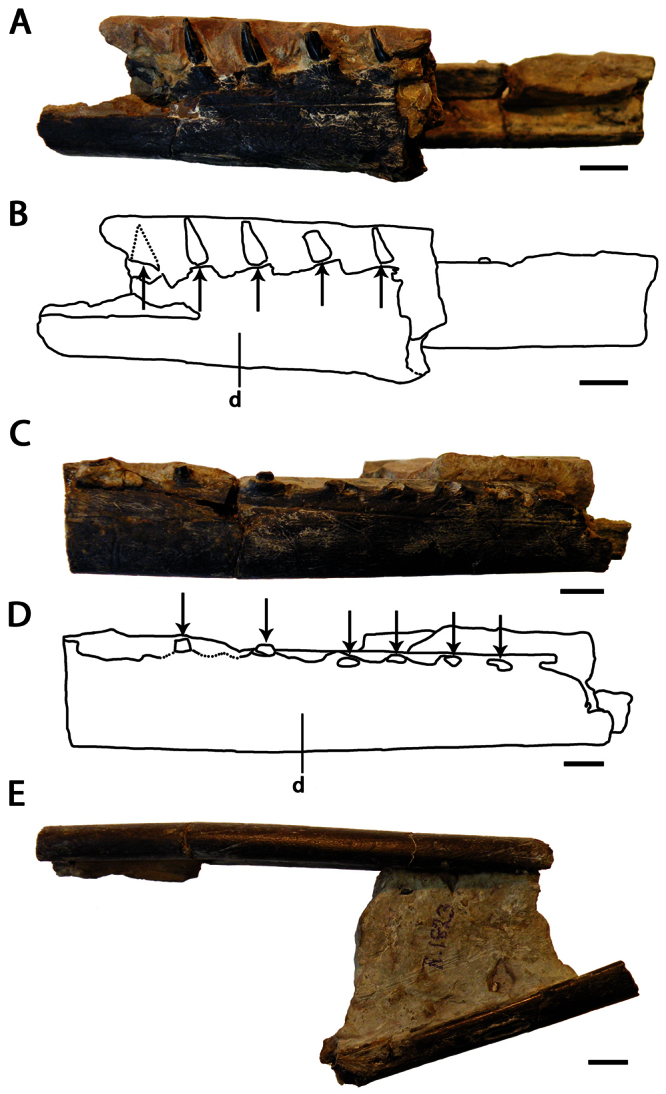
*Pterodactylus sagittirostris*, holotype NHMUK PV R 1823 (upper Berriasian / Valangianian, Hastings Group), part of the mandibular rami. **A** right lateral view **B** respective line drawing **C** left lateral view **D** respective line drawing **E** ventral view. Abbreviation: **d** – dentary. Arrows indicate alveoli or teeth. Scale bar = 10 mm. Photos courtesy of The Natural History Museum.

NHMUK PV R 1823, partial mandibular rami (Fig. 20A–E).

##### Type locality.

St.–Leonards–on–Sea, Sussex. England.

##### Type horizon.

Hastings Group, Wealden (late Berriasian / Valanginian).

##### Description.

The holotype of *Pterodactylus sagittirostris* consists of partial associated mandibular rami. The specimen is not comparable to any of the species referred in *Lonchodectes* by Hooley (1914) and Unwin (2001) because they are mostly based on jaw tips. Its teeth are elongated and differ from the shorter ones present in *Lonchodraco giganteus*, and hence *Pterodactylus sagittirostris* cannot be referred to Lonchodraconidae (which corresponds more or less to the Lonchodectidae *sensu* Unwin 2001). An apparent elevation of the alveolar margin, forming a collar around the teeth, rather seems to be an artifact of the partial preparation of the specimen; in the right ramus, which has been more extensively prepared, this collar is, in fact, smaller and resembles the one observed in other pterosaurs, such as anhanguerids. This specimen presents no diagnostic characters or character combination, and therefore *Pterodactylus sagittirostris* is considered a *nomen dubium*.

### Nomina nuda

As explained above, in 1869 Seeley created several names for pterosaur taxa from the Cretaceous of England, but these nomenclatural acts were disclaimed and, therefore, intentionally unavailable.

### Taxa from other deposits

**Figure 21.**
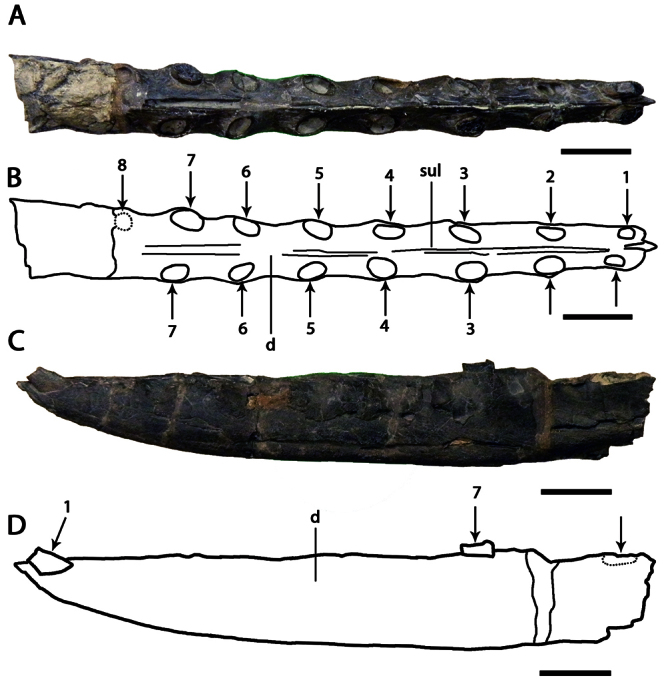
*‘Ornithocheirus’ wiedenrothi*, holotype SMNS 56628 (Hauterivian, Engelbostel clay pit, Hannover), anterior part of the mandibular symphysis. **A** dorsal view **B** respective line drawing **C** left lateral view **D** respective line drawing. Abbreviations: **d** – dentary, **sul** – sulcus. Arrows and numbers indicate alveoli or teeth and their respective position. Scale bar = 10 mm.

With the genus *Ornithocheirus* used as a wastebasket for the Cambridge Greensand species with unknown relationships, fragmentary specimens from other regions of the world have ended up being referred to *Ornithocheirus* as well. Among them is *‘Ornithocheirus’ wiedenrothi* Wild, 1990 (holotype SMNS 56628; Fig. 21A–D), known from two pieces of a mandibular symphysis, a right articular, and fragments of wing bones from the Hauterivian of Germany (Wild 1990). In light of the identification of *Ornithocheirus simus* as the type species of *Ornithocheirus*, it is clear that, although not directly comparable, *‘Ornithocheirus’ wiedenrothi* can be excluded from this genus, as recently noted by Fletcher and Salisbury (2010). *‘Ornithocheirus’ wiedenrothi* is quite interesting as it possesses a large and sharp process on the tip of the symphysis (Wild 1990), unknown in all so far described pterosaurs. It also has a comparatively large first pair of alveoli at the tip of the mandible; in the British taxa, the lower jaw has a smaller first pair of alveoli followed by larger ones posteriorly.

Another occurrence of the *Ornithocheirus* complex is *‘Ornithocheirus’* sp. A, based on NHMUK PV R 11958, a mandible from the Purbeck Limestone Formation of England (Berriasian) (Howse and Milner 1995). The specimen lacks the posterior ends of the mandibular rami but shows an elongate symphysis with a slight anterior expansion. Unfortunately, the specimen is unprepared and only exposed in ventral view, precluding observations on its dentition. A small prepared area and viewing from the sides established that it bears alveoli and a mandibular sulcus. Other than that, it is not possible to refer it to a known clade without more extensive preparation.

ZIN PNT–S50–1, a partial mandibular symphysis from the Cenomanian of Lysaya Gora Hill, Saratov district, in the southern European part of Russia, has been referred either as *Ornithocheirus(?)* sp. (Khozatskii 1995), *Anhanguera* cf. *Anhanguera cuvieri* (Bakhurina and Unwin 1995), and as cf. *Anhanguera* (Unwin and Bakhurina 2000). Unwin and Bakhurina (2000) state that it has an anterior expansion with relatively large teeth, a mandibular groove, and an upward curved mandible. Based on the available illustration, the specimen represents a pteranodontoid based on the presence of a dentary dorsal groove. The symphysis is narrow and the alveoli are uniform in size and distribution, as in many of the species of the *Ornithocheirus* complex. An upward curvature of the upper jaw is common among these species, but has not been reported in lower jaws and deserves further investigation. Contrary to the description, no anterior expansion or larger anterior teeth can be observed. Furthermore, no sagittal crest was reported, and thus this Russian specimen can be excluded from *Anhanguera* and the clade Anhangueridae and from *Lonchodraco*. In any case, the geographic and temporal separation of ZIN PNT–S50–1 from definite species of the *Ornithocheirus* complex hints at the possibility that it represents a distinct taxon. The same locality has yielded a pelvis referable to Anhangueridae or a related taxon (Averianov 2004).

Additional remains from the territories of the former Soviet Union referred as ornithocheirids come from different localities in Russia and in Uzbekistan and include cranial and postcranial elements and isolated teeth. These records include a partial tip of the rostrum (specimen ZIN PH no. 50/44), referred as *Ornithocheirus* sp., from the Khodzhakul locality in Uzbekistan (upper Albian or lower Cenomanian; Averianov 2007). Unfortunately, this specimen is fragmentary, being represented by part of a transverse section of a jaw and is non–diagnostic.

QM F10613, a mandibular symphysis from Albian Toolebuc Formation of Australia, has been referred to aff. *Ornithocheirus* sp. (Molnar and Thulborn 1980), *Anhanguera*? *cuvieri* (Unwin et al. 2000), aff. *Lonchodectes* sp. (Molnar and Thulborn 2007), and aff. *Ornithocheirus* (Myers 2010). Revision and comparison to the British pterosaurs of the *Ornithocheirus* complex and other pterosaur species has established that it represents a new genus and species, *Aussiedraco molnari* (Kellner et al. 2011).

*Aetodactylus halli*, from the Tarrant Formation (middle Cenomanian) of Texas, is known only from its holotype, SMU 76383. This specimen consists of a complete mandible, whose tip bears resemblances to those from the *Ornithocheirus* complex (Myers 2010). The species has been diagnosed by a subtle lateral expansion of the tip of the mandible, symphysis strongly compressed dorsoventrally, relatively constant spacing between the alveoli, and mandibular rami dorsally inflected (Myers 2010). Although the specimen was crushed, the symphysis was likely flattened in life (Myers 2010), but its position relative to the mandibular rami seems to be due to taphonomic factors. Myers (2010) assigned *Aetodactylus halli* to Ornithocheiridae based mostly on the presence of an anterior expansion. He also followed a taxonomic scenario that considers Anhangueridae and Boreopteridae as junior synonyms of Ornithocheiridae. In the present work, we propose that Ornithocheiridae should be restricted to *Ornithocheirus simus*, with which *Aetodactylus halli* cannot be directly compared. *Aetodactylus halli* has slightly raised alveolar collars but does not have the elevated alveolar margins present in lonchodraconids; it also lacks the dentary crest present in *Lonchodraco giganteus* and *Lonchodraco machaerorhynchus*. The presence of a slight anterior expansion distinguishes it from *‘Pterodactylus’ daviesii*. Myers (2010) provided a table with comparative measurements of different ‘ornithocheirid’ mandibles. Among these features, the most outstanding one is the proportion of the mandible bearing alveoli (toothed length %): 74% of the mandible of *Aetodactylus halli* bear alveoli, a number much higher than in anhanguerids, which compares well only with *Boreopterus cuiae* Lü & Ji, 2005 from the Barremian – Aptian Yixian Formation of China (see Lü and Ji 2005). *Boreopterus* and Boreopteridae represent a taxonomic problem of their own and are in need of review, which is beyond the scope of this work.

**Figure 22.**
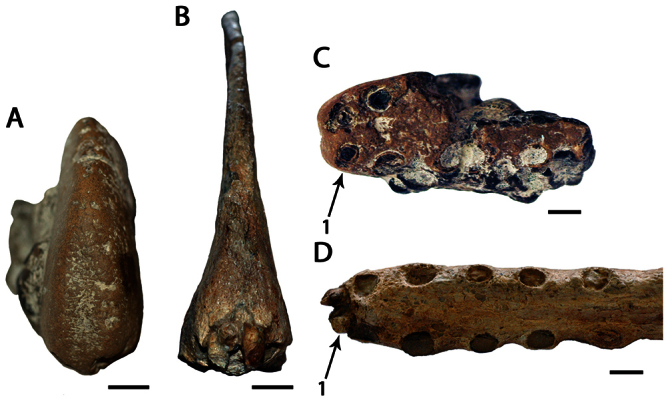
Comparison between *Ornithocheirus simus* and *Tropeognathus mesembrinus*. **A** and **C** *Ornithocheirus simus*, holotype CAMSM B54428 (Albian, Cambridge Greensand), anterior part of the rostrum **A** anterior view **C** ventral view **B** and **D** *Tropeognathus mesembrinus*, holotype BSP 1987 I 46 (Aptian / Albian, Romualdo Formation), anterior part of the rostrum **B** anterior view **D** ventral view. Arrows and numbers mark the position of the first pair of alveoli. Scale bar = 10 mm.

Lastly, the species of the *Ornithocheirus* complex from England have been compared with the anhanguerids and related taxa from the Romualdo and Crato formations of Brazil. As shown above, no species from the Cambridge Greensand or the Chalk Formation shows a combination of characters only found in the genus *Anhanguera*: the presence of an anterior expansion of the rostrum, a premaxillary crest that begins near the tip of the rostrum, and the fifth and sixth upper alveoli smaller than the fourth and seventh (Kellner 2003). Thus, this genus is here considered restricted to the Romualdo Formation in Brazil. In addition, *Tropeognathus mesembrinus* has been referred to *Ornithocheirus* (or *Criorhynchus*) (e.g., Unwin 2001, 2003, 2006). There is even one case where this species is regarded in three different genera within the same publication (Martill 2011): *Criorhynchus* (Introduction, first page), *Tropeognathus* (Table 1, second page), and *Ornithocheirus* (Affinities, fourth page). The holotype of *Tropeognathus mesembrinus* is a complete rostrum and mandible (BSP 1987 I 46), and there are two referred specimens: an almost complete mandible (SMNS 55414; Veldmeijer 2002) and a partial skeleton that includes an incomplete rostrum and mandible (MN 6594–V; Kellner et al. 2013). *Tropeognathus* is distinguished from *Ornithocheirus* by the position of the first pair of alveoli (Veldmeijer 2003). Whereas in all specimens referable to *Ornithocheirus simus* the first alveoli are located slightly posterior to the ventral margin of the premaxillae and are directed downwards (Figs 1, 2, 3, 22A, C), they are located on the anterior tip of the rostrum in *Tropeognathus mesembrinus* on a dorsally reflected palate (Fig. 22B, D), a derived feature present in different degrees in several other toothed pteranodontoids, especially anhanguerids, but absent in all specimens referrable to *Ornithocheirus simus*. One reviewer suggested that perhaps the use of other methods, such as computed tomography scans, could help clarify the problem of the synonymy between *Tropeognathus* and *Ornithocheirus*, since it is unlikely that more complete specimens of *Ornithocheirus simus* will ever be recovered from the Cambridge Greensand. Maybe paleohistological analyses could bring useful data to the matter as well. Despite being known by several complete crania (including some still undescribed), the range of individual, ontogenetic and sexual variation in anhanguerids is still poorly understood. This is especially true because most of these crania are isolated, and associated postcranial material that would enable the identification, for instance, of the osteological maturity of the individuals based on size–independent criteria, is very rare (Wellnhofer 1991b; Kellner and Tomida 2000; Veldmeijer 2003; Kellner et al. 2013). Therefore, in the present study, we chose to group into genera species that unambiguously share characters, and separate those species that have different features, with the aim of not forming non–monophyletic groups.

## Phylogenetic affinities of the species of the *Ornithocheirus* complex

The phylogenetic position of the species of the *Ornithocheirus* complexwithin Pterodactyloidea has been poorly studied. Kellner (2003) used *Pterodactylus compressirostris* (as *Ornithocheirus compressirostris*, but here considered a *nomen dubium*) in his cladistic analysis of the Pterosauria and recovered it as the sister group of Anhangueridae (*Anhanguera* and *Tropeognathus*). Such a result was also found in later, expanded versions of this analysis (Kellner 2004; Wang et al. 2005, 2008, 2009). The clade *Pterodactylus compressirostris* + Anhangueridae is supported by a single synapomorphy: presence of a discrete palatal ridge, tapering anteriorly (ch. 27.1) (Kellner 2003).

Lü and Ji (2006) used a modified version of the matrix compiled by Kellner (2004), adding 17 taxa of pterosaurs known from Liaoning at that time. It is beyond the scope of this paper to review this analysis in detail, but we note here some problems regarding the coding of characters in *Liaoningopterus gui*. This species was found as a basal anhanguerid, in a sistergroup relationship with *Anhanguera* and *Tropeognathus*. According to their codings, *Liaoningopterus* differs from the other anhanguerids in the possession of a straight dorsal margin of the skull (ch 1.0), which was coded as wave–like (ch. 1.2) in *Anhanguera* and *Tropeognathus*. *Tropeognathus* would have a comparatively broad lower jaw (ch. 2.1), rather than mediolaterally compressed as scored by Kellner (2003, 2004). *Liaoningopterus* would have the rostrum low with a straight or concave dorsal outline (ch. 16.1), whereas *Anhanguera* and *Tropeognathus* have a tall anterior region of rostrum but low antorbital region (ch. 16.3). *Liaoningopterus* would have teeth confined to the anterior part of the jaws (ch. 36.2), whereas in *Anhanguera* and *Tropeognathus* the teeth would be evenly distributed along the jaws (ch. 36.0). *Liaoningopterus* would have peg–like teeth, 15 or fewer on each side of the jaws (ch. 41.1), whereas *Anhanguera* and *Tropeognathus* would not have peg–like teeth (ch. 41.0). First–hand examination of the holotype of *Liaoningopterus gui* (IVPP V13291), however, demonstrates that it is remarkably similar to *Anhanguera* and shows that these different codings are incorrect. Instead, *Liaoningopterus gui* should have identical codings to *Anhanguera* for all of the aforementioned characters, and the view that it is a basal anhanguerid can no longer be supported. Furthermore, *Tropeognathus* has a mediolaterally compressed rather than broad lower jaw.

Andres and Ji (2008) also included *Pterodactylus compressirostris* (as *Lonchodectes compressirostris*) in their phylogenetic analysis of Pterodactyloidea and, as by Kellner (2003), *Pterodactylus compressirostris* was recovered as the sister group of Anhangueridae (*Anhanguera*, *Liaoningopterus* and *Tropeognathus*). This clade was supported by two synapomorphies: presence of a palate with ridge and mandible with sulcus (ch. 39.1) and presence of spike–shaped teeth with wide, subcircular bases (ch. 56.5). *Liaoningopterus* was not recovered as a basal anhanguerid, but rather formed a trichotomy with *Tropeognathus* and *Anhanguera*.

The phylogenetic studies of Kellner (2003) and Andres and Ji (2008) pointed out that *Pterodactylus compressirostris* is placed close to but outside Anhangueridae. Because *Pterodactylus compressirostris* is considered a *nomen dubium* here, the aforementioned phylogenies have little to add concerning the position of *Ornithocheirus*, *Lonchodraco*, or other species of the *Ornithocheirus* complex discussed here.

Unwin (1995) proposed a phylogeny for pterosaurs, using supra–specific taxa (genera or families) as terminal groups. In this scheme, Ornithocheiridae was recovered as the sister group of *Pteranodon*, with *Ornithodesmus* [=*Istiodactylus*] as its immediate sister group, forming a clade Unwin named Ornithocheiroidea (which has a different composition from the homonymous clade of Kellner 2003). There is a list of apomorphies but, unfortunately, no data matrix was provided to allow testing his results. A similar cladogram was presented by Unwin (2001) in his review of the Cambridge Greensand pterosaurs, with Pteranodontidae as the sister group of Ornithocheiridae, and *Istiodactylus* as their sister group. *Nyctosaurus* was added and positioned as the sister group of the remaining ornithocheiroids. Lonchodectidae (*sensu* Unwin 2001) was added and placed outside Ornithocheiroidea, in a trichotomy with Ctenochasmatoidea and a clade including Dsungaripteroidea (which is also different from the homonymous clade of Kellner 2003) and Azhdarchoidea. Unwin (2001) notes that this phylogeny was based on Unwin (1995), Unwin and Lü (1997) and Unwin et al. (2000). However, none of these papers included a data matrix, only lists of putative synapomorphies, so these results are not testable.

Unwin (2003) analyzed the phylogenetic relationships of Pterosauria, again using genera and families as terminal taxa. This time, a data matrix was provided and Ornithocheiridae was found in a clade Unwin named Euornithocheira, as the sister group of Pteranodontidae + *Nyctosaurus*, with *Istiodactylus* as their sister group. Euornithocheira was supported by three synapomorphies: concave posterior margin of nasoantorbital fenestra (ch. 39.1), basal region of orbit infilled (ch. 40.1), and coracoid facets of the sternum lateral to each other (ch. 41.1). Lonchodectidae (*sensu* Unwin 2001) was recovered in the clade Euctenochasmatia, which consisted of a trichotomy between *Pterodactylus*, Ctenochasmatidae and Lonchodectidae, with *Cycnorhamphus* as their sister group. Euctenochasmatia was supported by two synapomorphies: neural arch of the mid–series cervicals depressed and with low neural spine (ch. 52.1) and elongate mid–series cervicals (ch. 53.1) (Unwin 2003).

Lü et al. (2010) undertook a cladistic analysis of the Pterosauria, using genera as terminal groups, and yet another position for Lonchodectidae (*sensu* Unwin 2001) was found. In this work, *Lonchodectes* (*sensu* Unwin 2001) was recovered in a clade which all other representatives are edentulous pterosaurs: tapejarines, thalassodromines, azhdarchids and *Chaoyangopterus* (Azhdarchoidea). The matrix was analyzed with the software PAUP* (Swofford 2003); however, the search was interrupted after finding 500,000 trees due to computer memory limitations.

The cladistic matrix of Lü et al. (2010) was, therefore, reanalyzed through the heuristic search in PAUP*, without interruptions, in a Dell computer with Intel Core i5 2.67 GHz processor and 6 GB RAM memory. The search lasted 4 hours and 23 minutes and yielded 845,093 equally parsimonious trees, with a length of 374 steps each. These trees are shorter than the ones reported by Lü et al. (2010), which had 400 steps. The consistency index (CI) was 0.44, homoplasy index (HI) 0.56, retention index (RI) 0.80 and rescaled consistency index (RC) 0.35. It is worth noticing that all these indexes are lower than the ones reported by Lü et al. (2010), despite using the same character–taxon matrix and the same software for phylogenetic analysis.

**Figure 23.**
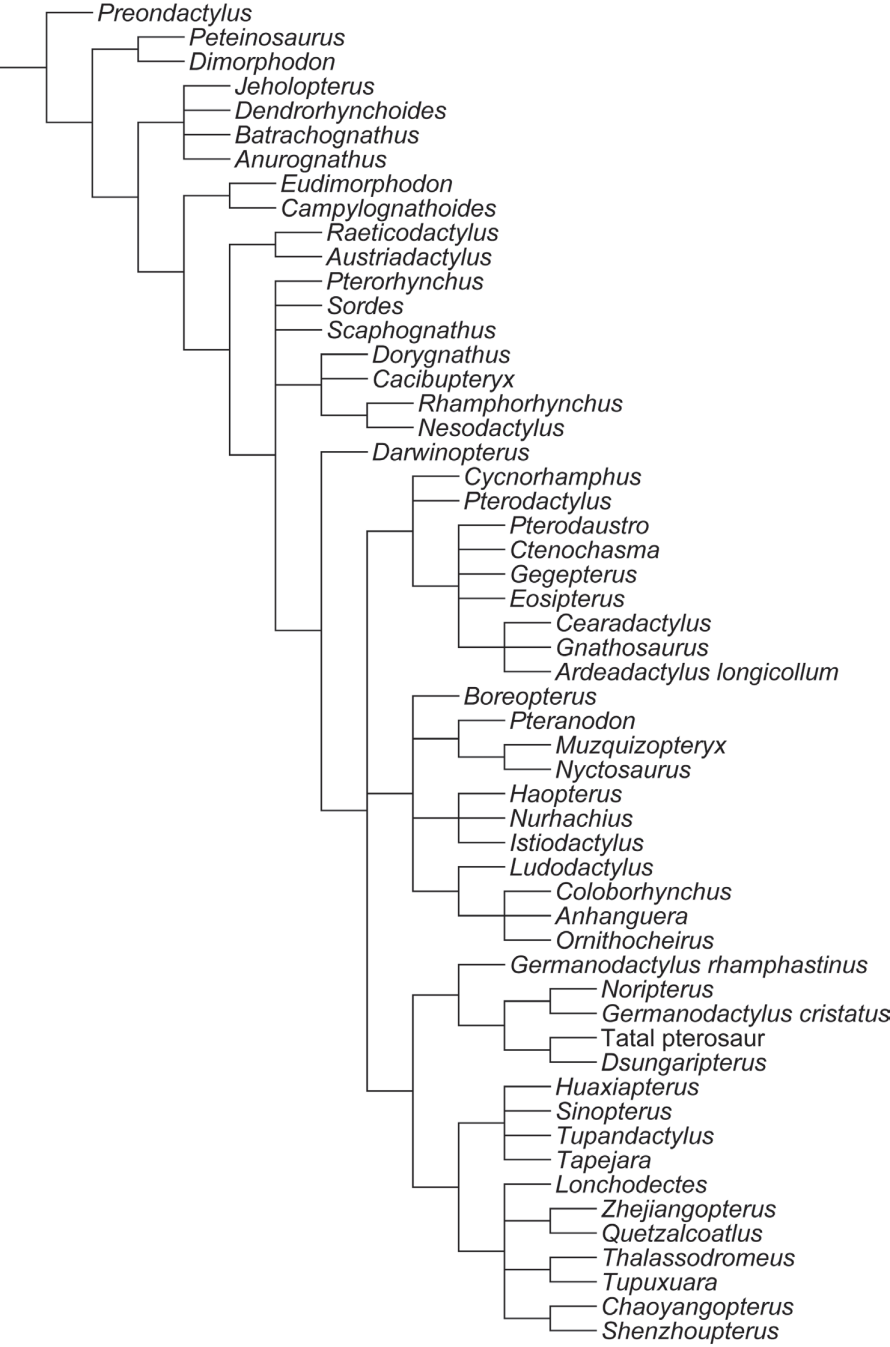
Strict consensus tree of the reanalysis of the matrix by Lü et al. (2010). *Lonchodectes* is used *sensu* Unwin (2001).

In addition, the strict consensus tree from this reanalysis shows some differences in relation to the one published by Lü et al. (2010) (Fig. 23; the terminal taxon *Pterodactylus longicollum* is here named *Ardeadactylus longicollum* following Bennett [2012]). Among non–pterodactyloid pterosaurs, *Campylognathoides* and *Eudimorphodon* were recovered in more basal positions, and *Austriadactylus* and *Raeticodactylus* were recovered as a monophyletic group. Among pterodactyloids, the published consensus tree does not include *Noripterus*, whereas the matrix (provided as supplementary material), does. As a result, in the reanalysis *Germanodactylus* was recovered as paraphyletic. In both analyses *Lonchodectes* (*sensu* Unwin 2001) was found in Azhdarchoidea. However, in the analysis by Lü et al. (2010), Azhdarchoidea is presented as a large polytomy, with only Tapejarinae (*Tapejara*, *Tupandactylus*, *Sinopterus* and *Huaxiapterus*) and Azhdarchidae (*Quetzalcoatlus*, *Zhejiangopterus* and *Azhdarcho*) recovered as monophyletic. The available data matrix does not include *Azhdarcho* and thus this species is not present in the reanalysis. The reanalysis recovered Azhdarchoidea divided into two clades, Tapejarinae and a second including Azhdarchidae, Thalassodrominae, Chaoyangopteridae and *Lonchodectes* (*sensu* Unwin 2001) in a polytomy.

Identical topology to the one found in the strict consensus tree of the reanalysis using PAUP* was recovered analyzing the same matrix in TNT (Goloboff et al. 2003, 2008), through the traditional search and the software’s default options, which, according to Goloboff et al. (2008), is roughly equivalent to the heuristic search with random addition sequences in PAUP*. Very little memory was required for the data and the search took less than one second.

It is interesting to note that the analyses of Unwin (2003) and Lü et al. (2010), which included all species of *Lonchodectes sensu* Unwin (2001) recovered *Lonchodectes* in positions among the Pterodactyloidea that are very distinct from the positions recovered by Kellner (2003) and Andres and Ji (2008) for *Pterodactylus compressirostris*. Therefore, the reasons that led to such position are analyzed here.

The analyses of Unwin (2003) and Lü et al. (2010) are problematic in two ways. First, the use of genera as operational taxonomic units (OTUs) is problematical in the case of speciose genera, as is the case for *Lonchodectes sensu* Unwin (2001). In the present work, two species referred by Unwin (2001) to *Lonchodectes* are considered *nomina dubia*, including the type species, what resulted in three species being transferred to a new genus, and another is referred to a possibly distinct genus; such referrals alone question the position of *Lonchodectes* found by the aforementioned works because the genus is probably not monophyletic.

A second dispute involves the use of postcranial material in the analyses of Unwin (2003) and Lü et al. (2010). As indicated previously (Hooley 1914; Kellner 1990; Unwin 2001; Martill 2011), pterosaur cranial and postcranial material have not been found in association in the Cambridge Greensand. In the case of specimens from the Chalk Formation, only the holotype of *Lonchodraco giganteus* [=*Lonchodectes giganteus* *sensu* Unwin 2001] has associated postcranial material. Hence, there are no ways to objectively associate postcranial material with *Lonchodectes* (*sensu* Unwin 2001), except for scapulocoracoids similar to *Lonchodraco giganteus* (which is incomplete). Despite present in expressive number, certain cervical vertebrae and humeri known from the Cambridge Greensand (excluding those similar to Anhangueridae) cannot be objectively referred to taxa mostly known by upper and lower jaw tips. It is also worth noting that an edentulous pterosaur is known from the Cambridge Greensand, *Ornithostoma* (Unwin 2001).

The cervical vertebrae that Unwin (2001, 2003) and Lü et al. (2010) refer to Lonchodectidae (*sensu* Unwin 2001) (for example, NHMUK PV R 2287c; Unwin 2003) were originally described as caudals (e.g., Seeley 1875). They are elongated (Unwin 2001) and have vertebral centra with very reduced or without pneumatic foramina. Their neural spines are low (Unwin 2001). These characters are reminiscent of Azhdarchidae. Similarly, the humeri possibly referred as lonchodectids (*sensu* Unwin 2001) (for instance, CAMSM B54081; Unwin 2003) have a straight deltopectoral crest (Unwin 2001) and ventral pneumatic foramina in the proximal and distal portions, similar to Azhdarchoidea. Both elements were also referred to azhdarchoids by Averianov (2012), specifically to *Ornithostoma sedgwicki*.

**Figure 24.**
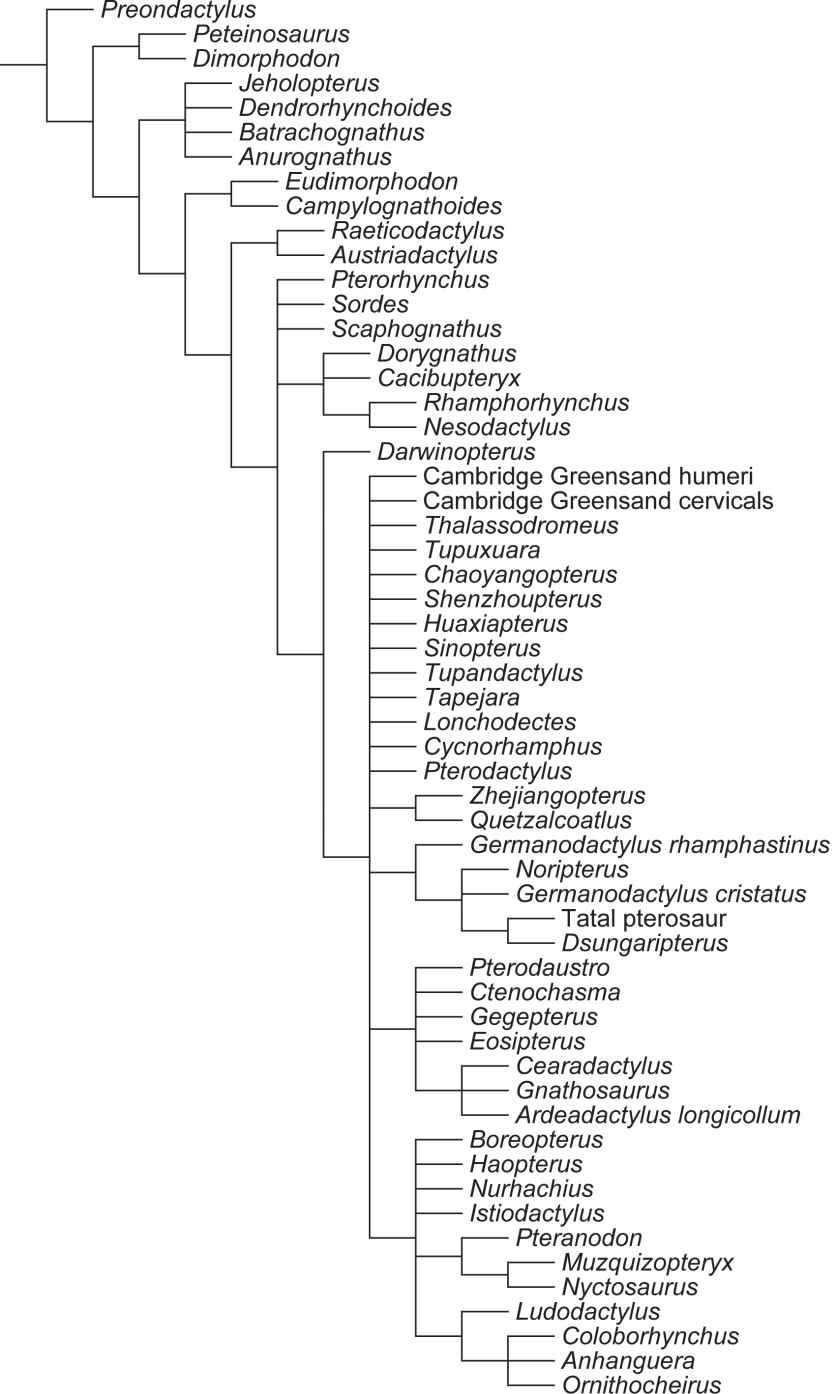
Strict consensus tree of the reanalysis of the matrix by Lü et al. (2010), with the codings of the humeri and cervical vertebrae in *Lonchodectes* (*sensu* Unwin 2001) as separate OTUs.

**Figure 25.**
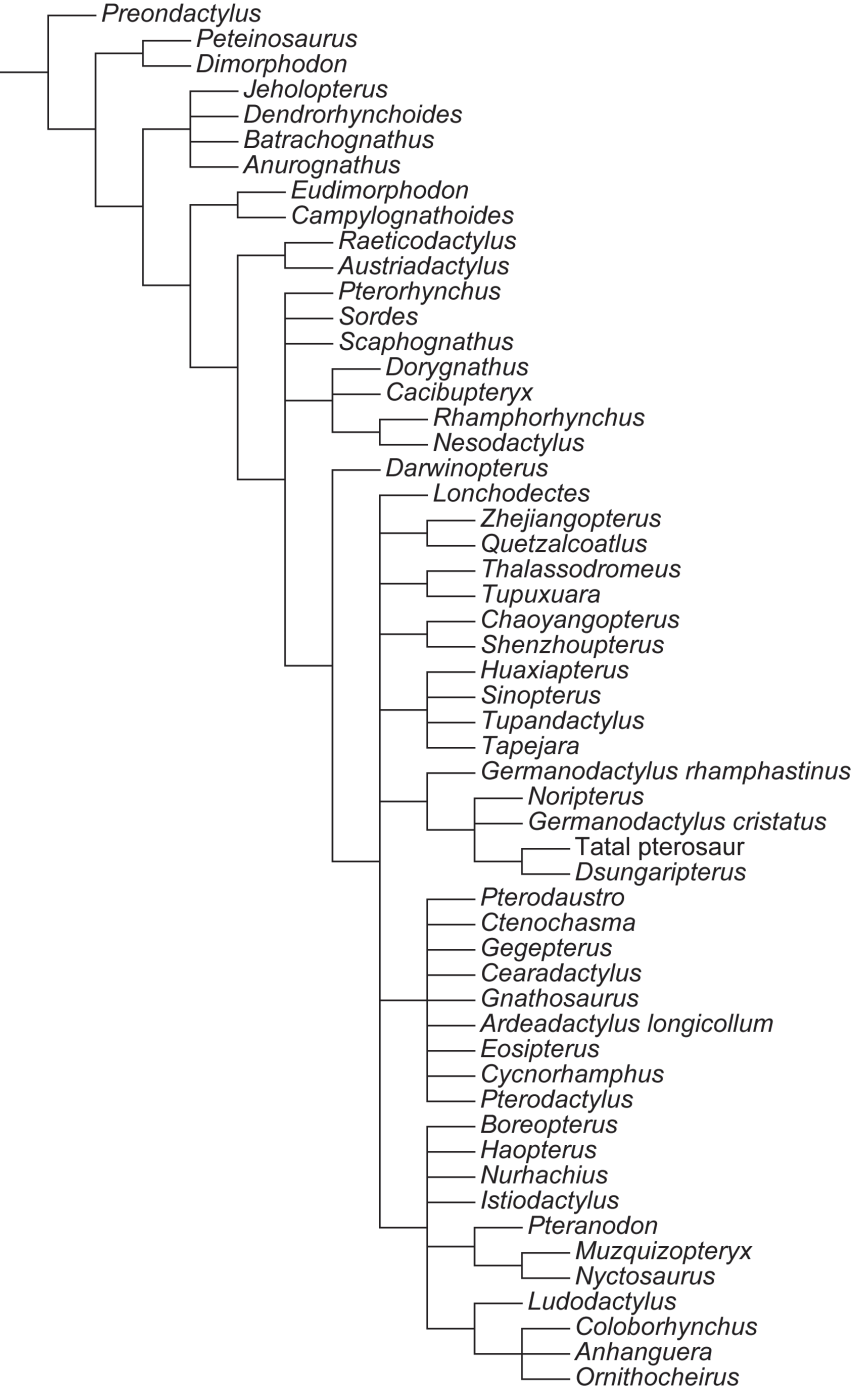
Strict consensus tree of the reanalysis of the matrix by Lü et al. (2010), with the OTU *Lonchodectes* (*sensu* Unwin 2001) restricted to codings of skull and scapulocoracoid characters.

Therefore, reanalyses using the humeri and cervical vertebrae as separate OTUs were undertaken, using the data matrices of Lü et al. (2010) and Wang et al. (2008). In the matrix by Lü et al. (2010), only the rostrum, mandible and scapulocoracoid characters were maintained in the OTU *Lonchodectes* (*sensu* Unwin 2001), and two new OTUs were inserted with the character states of cervical vertebrae and humeri, as present in the original matrix. Traditional search in TNT resulted in 40 equally parsimonious trees (noting that the TNT algorithm works with global optima or tree islands, and does not recover all possible trees) with a length of 373 steps each. In the strict consensus tree (Fig. 24), the main groups of Pterodactyloidea were collapsed to its base. Even when the two new OTUs are *a priori* excluded, the results (60 equally parsimonious trees, 373 steps each) show low resolution in the position of *Lonchodectes* (*sensu* Unwin 2001) (Fig. 25). These results indicate that the phylogeny by Lü et al. (2010) loses resolution when *Lonchodectes* is restricted only to material that can be undoubtlessly referred to the nominal species of this genus, *sensu* Unwin (2001).

**Figure 26.**
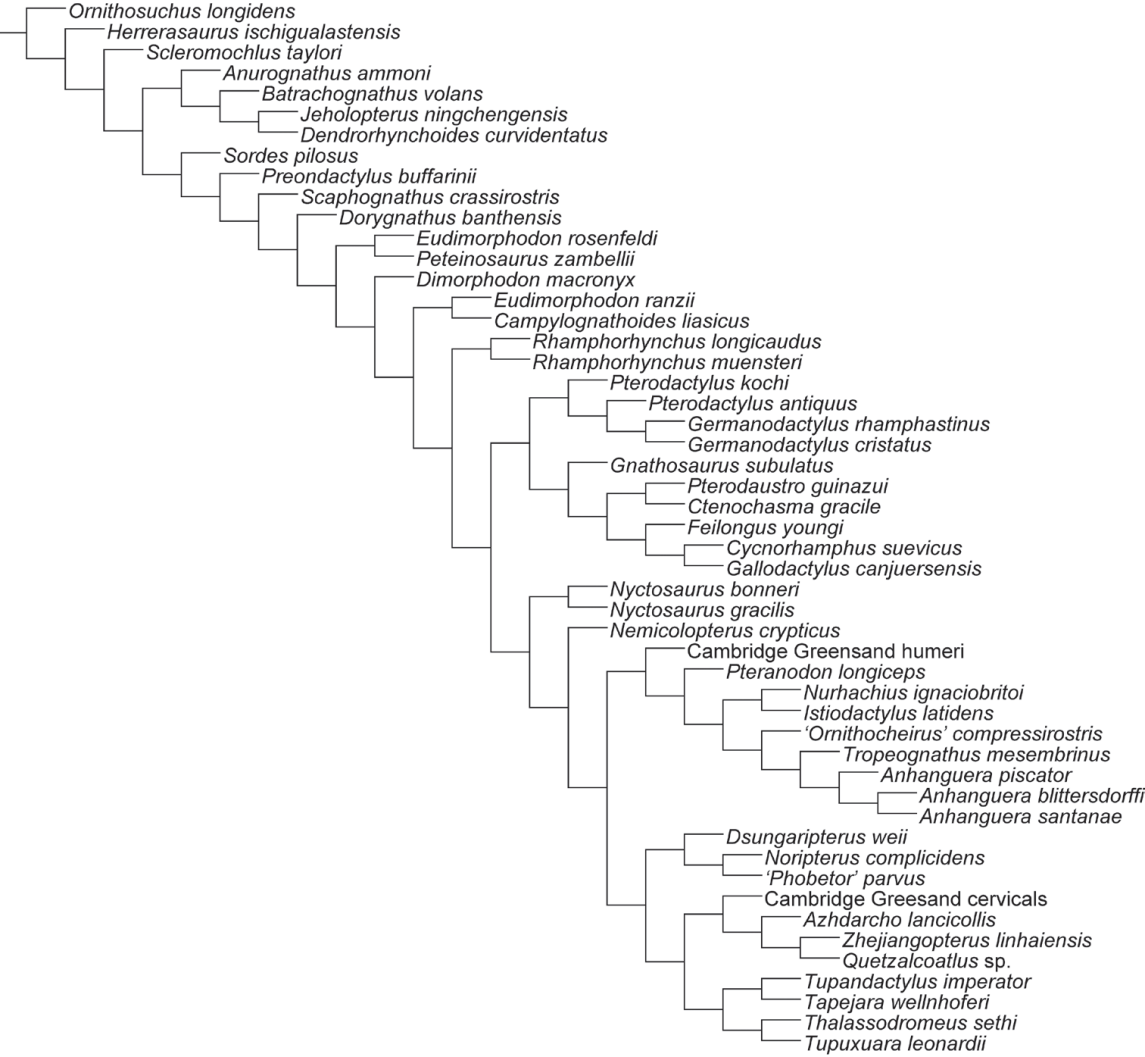
Strict consensus tree of the reanalysis of the matrix by Wang et al. (2008), with the humeri and cervical vertebrae referred to *Lonchodectes* by Lü et al. (2010) included as separate OTUs.

In the case of the matrix by Wang et al. (2008), both OTUs (cervical vertebrae and humeri) were inserted and the matrix was analyzed through the traditional search in TNT. The search resulted in a single tree with 195 steps (Fig. 26). The elongated cervical vertebrae were recovered in the Azhdarchidae, while the humeri were positioned basally in the Pteranodontoidea. Such results suggest that these elements pertain to distinct clades.

In order to access the phylogenetic relationships of the species of the *Ornithocheirus* complex, we used a slightly modified version of the character matrix of Wang et al. (2009; see appendix), with the addition of 24 taxa: *Anhanguera robustus*, *Anhanguera spielbergi*, *Anhanguera araripensis*, *Liaoningopterus gui*, *Coloborhynchus clavirostris*, *‘Ornithocheirus’ capito*, *Caulkicephalus trimicrodon*, *Ludodactylus sibbicki*, *Brasileodactylus araripensis*, *Camposipterus nasutus*, *Camposipterus(?) sedgwickii*, *Camposipterus(?) colorhinus*, *Cimoliopterus cuvieri*, *Ornithocheirus simus*, *Cearadactylus atrox*, *‘Cearadactylus’ ligabuei*, *Lonchodraco giganteus*, *Lonchodraco machaerorhynchus*, *Lonchodraco(?) microdon*, *‘Ornithocheirus’ platystomus*, *‘Pterodactylus’ daviesii*, *‘Ornithocheirus’ denticulatus*, *‘Ornithocheirus’ polyodon*, and *Aussiedraco molnari*, and the exclusion of *Pterodactylus compressirostris*. The matrix was analyzed in TNT, using the traditional search option with default parameters.

**Figure 27.**
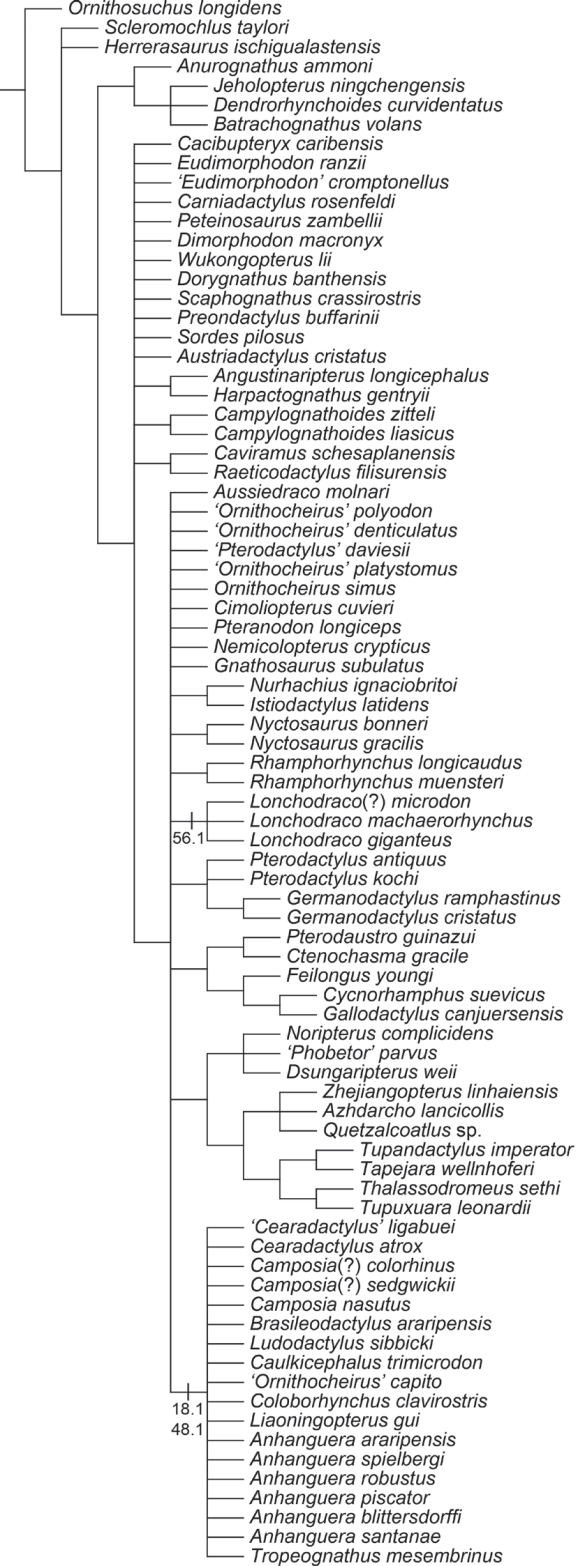
Strict consensus tree of the analysis including the species of the *Ornithocheirus* complex (see text for details).

The run with all OTUs (with a total of 81 taxa, three of them outgroups, all characters treated as unordered) resulted in 30 most parsimonious trees with a length of 246 steps each. Several nodes were collapsed in the strict consensus tree, including some non–pterodactyloid taxa (as noted by Wang et al. 2009) and many dsungaripteroids (Fig. 27). This is expected due to the large quantity of missing data added. Although the main pterosaur families were recovered as monophyletic entities (i.e., Istiodactylidae, Nyctosauridae, Dsungaripteridae, Azhdarchidae, Tapejaridae), their relationships with one another were obscured. All anhanguerids were recovered in a large polytomy that also included *Cearadactylus atrox*, *‘Cearadactylus’ ligabuei*, *Camposipterus nasutus*, *Camposipterus(?) sedgwickii*, *Camposipterus(?) colorhinus*, *Brasileodactylus araripensis*, and *Ludodactylus sibbicki*; this clade is defined by the anterior expansion of the premaxillary tip with the jaw end high (ch. 18.1) and the larger teeth located at the tip of the rostrum (ch. 48.1, a new state added to a character from the original matrix of Wang et al. 2009). The recovery of *Camposipterus* as paraphyletic is possibly due to the incompleteness of the specimens, with a large amount of missing data, and does not necessarily reflect their true relationships. The Lonchodraconidae was recovered as monophyletic (*Lonchodraco giganteus*, *Lonchodraco machaerorhynchus*, and *Lonchodraco(?) microdon*), supported by the presence of “parapet–like” alveoli (ch. 56.1, new character), as proposed by Unwin (2001) for the Lonchodectidae. The other added taxa, including *Ornithocheirus simus*, were found in a large polytomy.

The low resolution of the consensus tree is likely due to only a few taxa whose position changes greatly between different input trees (e.g., Butler and Upchurch 2007). Goloboff et al. (2008) proposed several options to detect these wildcard taxa using TNT. One of them, the agreement subtree, is a constriction tool that only shows the subset of taxa identically related in all input trees (Goloboff et al. 2008), thus excluding all polytomies. Because it prunes taxa after the search, this tree retains information retrieved from these taxa, while this does not occur when they are deleted before the search (e.g., Butler and Upchurch 2007). With pterosaurs, pruning of wildcard taxa has already been tested with non–pterodactyloids, with different results in comparison to their deletion (Rodrigues and Kellner 2010).

**Figure 28.**
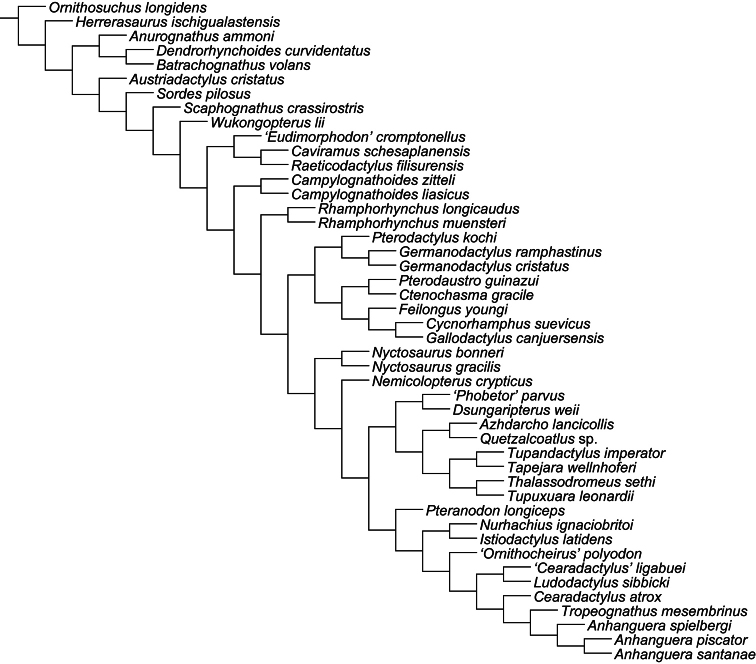
Agreement subtree of the analysis including the species of the *Ornithocheirus* complex.

The agreement subtree of our analysis had only 46 taxa of the original 81 and presented a monophyletic *Anhanguera*, with *Tropeognathus mesembrinus*, *Cearadactylus atrox*, the clade comprising *Ludodactylus sibbicki* and *‘Cearadactylus’ ligabuei*, and *‘Ornithocheirus’ polyodon* as successive sister groups (Fig. 28). The recovery of *Anhanguera piscator* and *Anhanguera santanae* as sister groups, and *Anhanguera spielbergi* as sister group to the both, is an artifact: the synapomorphy of both species is the loss of the notarium, but this structure is present only in adult specimens, while *Anhanguera piscator* and *Anhanguera santanae* (AMNH 22555) are known by only immature individuals. The recovery of *Cearadatylus atrox* as the proximate sister group of Anhangueridae was first shown by Vila Nova et al. (2010, in press). Although interesting, this tree does not have information on the relationships of the other species of the *Ornithocheirus* complex, so we used it as a base to add some of the unstable species. Knowing the synapomorphies of the agreement tree and which characters could be coded for the unstable taxa worked as a guide to choose which species not to prune.

**Figure 29.**
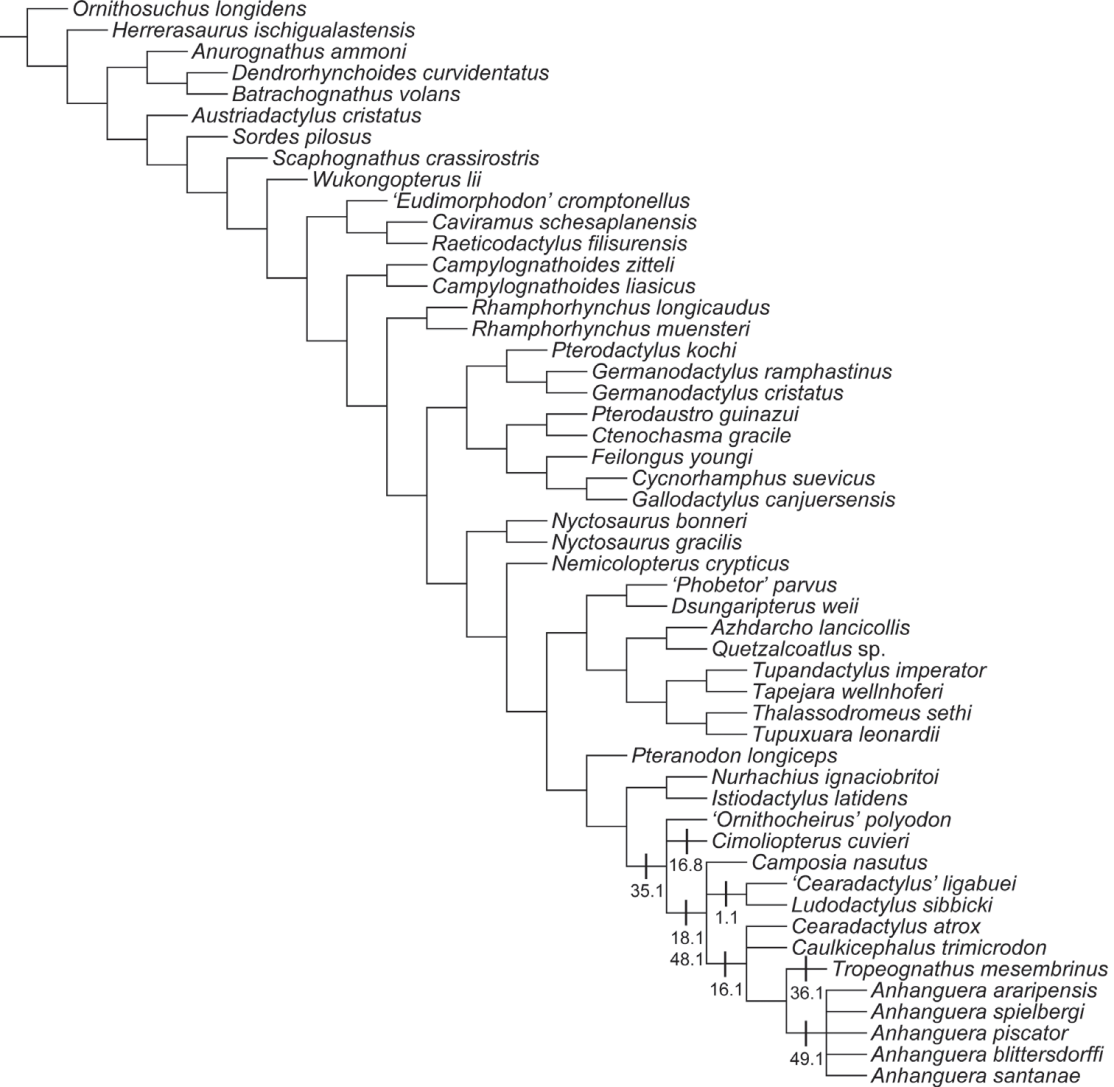
Agreement subtree of the analysis including the species of the *Ornithocheirus* complex with additional five taxa not pruned.

**Figure 30.**
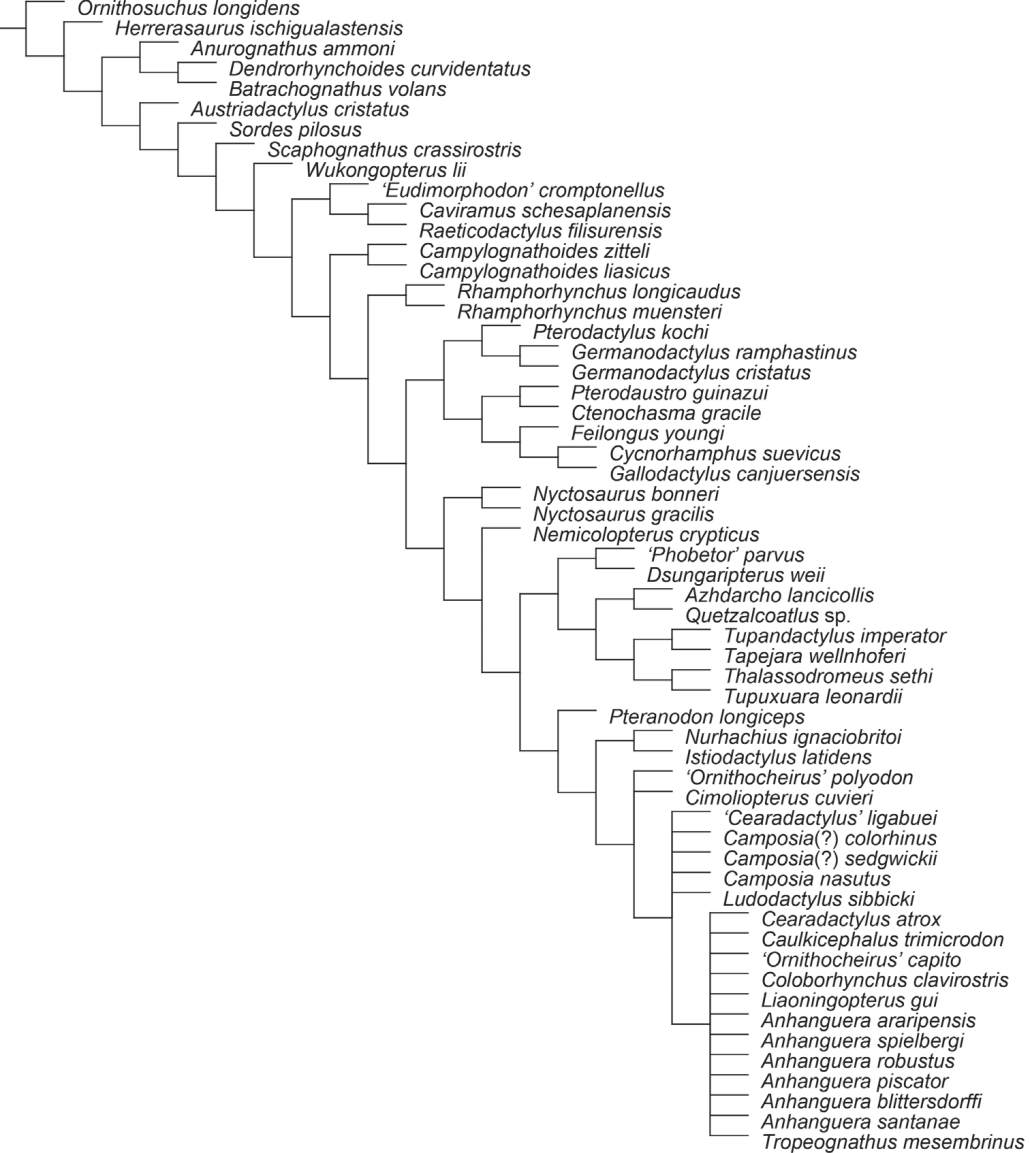
Agreement subtree of the analysis including the species of the *Ornithocheirus* complex with additional 11 taxa not pruned.

A new analysis was undertaken and, in comparison to the agreement subtree, five additional species were not pruned from the analysis: *Anhanguera araripensis*, *Anhanguera blittersdorffi*, *Caulkicephalus trimicrodon*, *Camposipterus nasutus*, and *Cimoliopterus cuvieri*. The resulting strict consensus tree (Fig. 29) recovered *Pteranodon longiceps* as the sister group to all other pteranodontoids, and Istiodactylidae as sister group to the remaining ones, which formed a monophyletic group, supported by the presence of a ridge on the palate and a sulcus on the mandible (ch. 35.1). This clade is composed of *Cimoliopterus cuvieri*, *‘Ornithocheirus’ polyodon*, and a clade with the remaining pteranodontoids, in a trichotomy. The latter, more restricted clade, is supported by the presence of an anterior expansion of the premaxillary tip with the jaw end high (ch. 18.1) and larger teeth located at the tip of the rostrum (ch. 48.1), and includes all pteranodontoids more closely related to *Anhanguera blittersdorffi* than to *Istiodactylus latidens* and *Cimoliopterus cuvieri*. This clade was also recovered when no taxa were pruned, and is here named Anhangueria (see above). Among anhanguerians, *Cearadactylus* was recovered as polyphyletic, but it is worthy of notice that this information could never have been retrieved if not for the complete preparation and subsequent redescription of the holotype of *Cearadactylus atrox* by Vila Nova et al. (2010, in press), years after *‘Cearadactylus’ ligabuei* was described by Dalla Vecchia (1993). *‘Cearadactylus’ ligabuei* was found in a sister group relationship with *Ludodactylus sibbicki*, united by the presence of a concave dorsal margin of the skull (ch. 1.1); due to incompleteness, it is unknown if *‘Cearadactylus’ ligabuei* had a frontal crest like *Ludodactylus*. Kellner and Tomida (2000) noted that part of the holotype may pertain to another individual. In any case, the rostrum of *‘Cearadactylus’ ligabuei* possessesa spatulate tip (Dalla Vecchia 1993), which is absent in *Ludodactylus*. Therefore, these taxa are distinct and most probably concern different genera. *Cearadactylus atrox* and *Caulkicephalus trimicrodon* were recovered in a polytomy with Anhangueridae; this clade was supported by the presence of an anteriorly located premaxillary crest (ch. 16.1). The ambiguous positionof *Caulkicephalus* is due to the fact that the lower jaw is still unknown, and the the presence of a dentary crest cannot be ascertained. All five species of *Anhanguera* were recovered as a monophyletic group, supported by the fifth and sixth superior alveoli smaller than the fourth and seventh as a synapomorphy (ch. 49.1), and *Tropeognathus mesembrinus* was recovered as a sister group of *Anhanguera*, as first retrieved by Kellner (2003). As more taxa are not pruned from the agreement subtree, they collapse some nodes, creating polytomies (Fig. 30).

**Figure 31.**
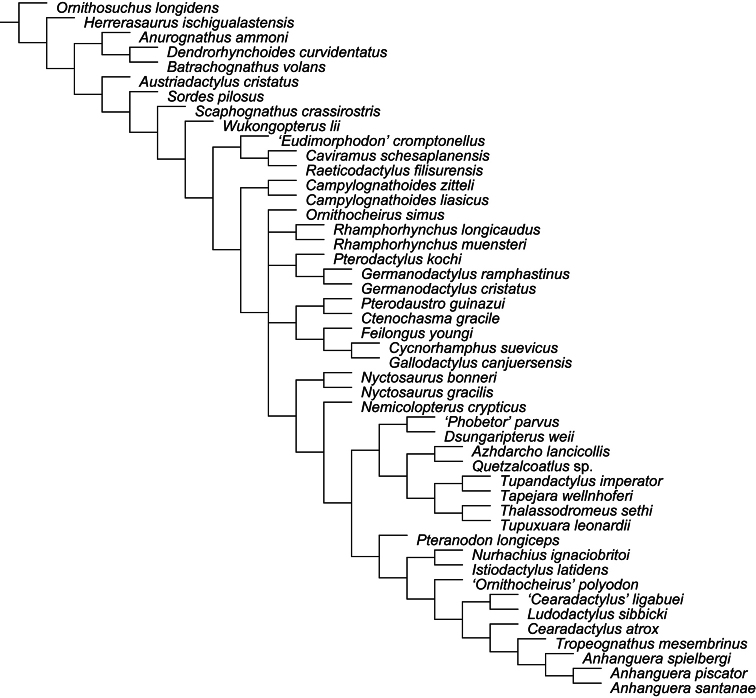
Agreement subtree of the analysis including the species of the *Ornithocheirus* complex with *Ornithocheirus simus* not pruned.

**Figure 32.**
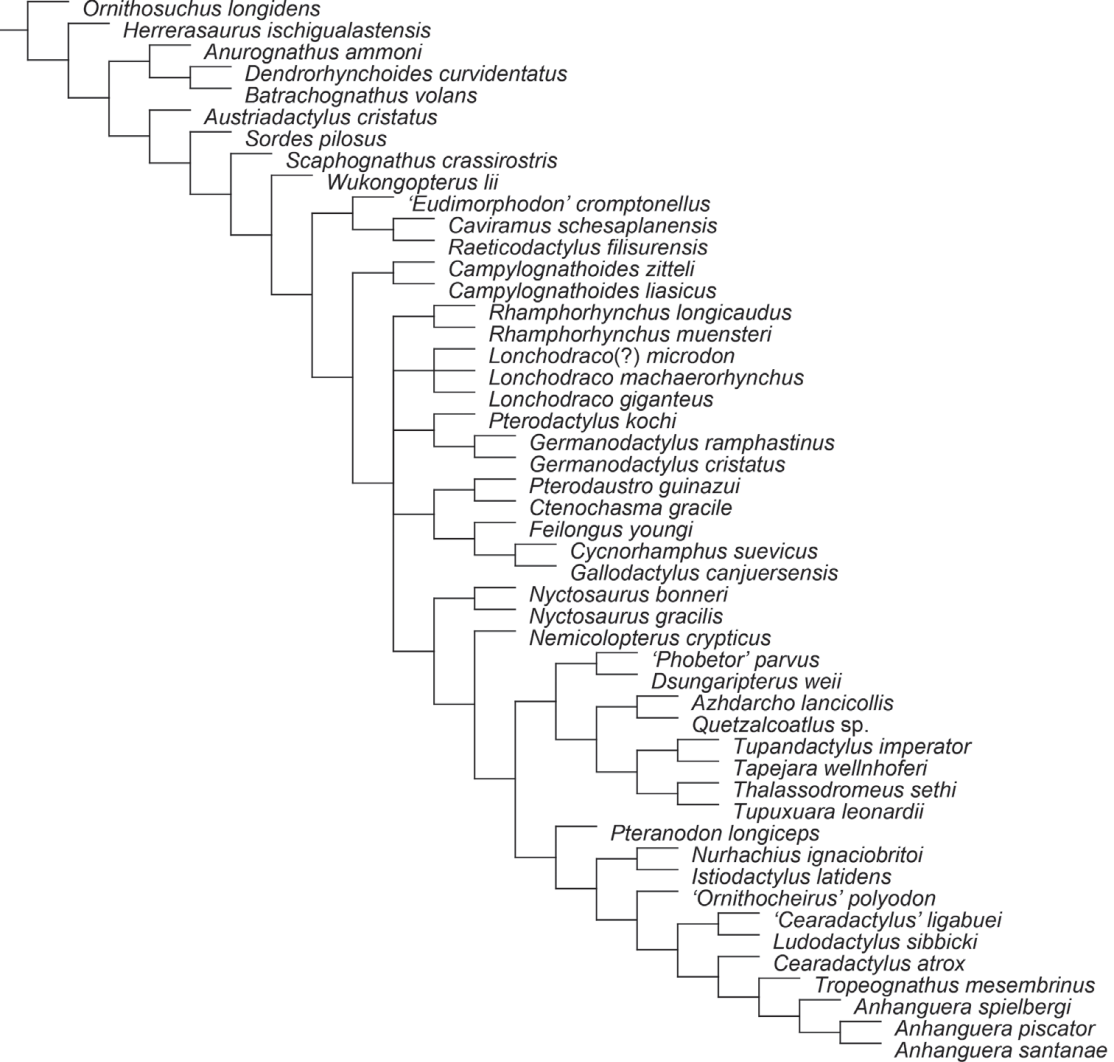
Agreement subtree of the analysis including the species of the *Ornithocheirus* complex with the Lonchodraconidae (more or less equivalent to the Lonchodectidae *sensu* Unwin 2001) not pruned.

The agreement subtree plus *Ornithocheirus simus* recovers this taxon in a polytomy at the base of Pterodactyloidea + Rhamphorhynchidae (Fig. 31). The same happens when Lonchodraconidae is not pruned in the agreement subtree (Fig. 32). This is possibly an artifact caused by the incompleteness of the known specimens, and they most likely nest closer to anhanguerians. New characters and more complete specimens, besides perhaps preparation (either mechanical or virtual) of the holotype and only known specimen of *Lonchodraco giganteus*, are needed to better evaluate this question.

## Conclusion

The species of the *Ornithocheirus* complex have been reviewed by several authors (e.g., Hooley 1914; Wellnhofer 1978; Unwin 2001). Review of such fragmentary specimens is challenging, both because many nomenclatural problems had to be sorted out, and because many important characters are missing in the holotypes. Some species were first cited in oral communications but not in print (for instance, Seeley 1864a, b). Several species and nomenclatural acts are not available (*nomina nuda*), because the nomenclatural acts were disclaimed (Seeley 1869). In this matter, the only species where we do not follow Unwin (2001) in treating them as *nomina nuda* are *Ornithocheirus oxyrhinus* Seeley, 1870, because it has a proper description and the publication is valid, and “*Ornithocheirus macrorhinus* Jukes–Browne, 1875” because Jukes–Browne (1875) referred to Seeley concerning its description and there clearly was no intent to name a species.

One would expect that, having so many species based on fragmentary material, several would prove to be non–diagnosable. However, Unwin (2001), the most recent reviewer, did not consider any species as *nomina dubia* (contra Martill 2010). In the present work, we consider 16 species *nomina dubia* (14 based on jaws; two are based on postcranial material and were not reviewed by Unwin [2001]). Unwin (2001) concluded that, among the species of the *Ornithocheirus* complex from the Cretaceous of England, only 13 are valid. We here consider 14 species valid.

Some species regarded by Unwin (2001) as valid are here considered non–diagnosable, such as *Pterodactylus compressirostris*, *Pterodactylus fittoni*, and *Pterodactylus sagittirostris*. On the other hand, some species here considered valid were synonymized with others by Unwin (2001), such as *Camposipterus nasutus*, *Camposipterus(?) colorhinus*, *‘Pterodactylus’ daviesii*, *‘Ornithocheirus’ denticulatus*, and *‘Ornithocheirus’ polyodon*. There are also species that Unwin (2001) considered synonymous with others but that are here regarded as *nomina dubia*: *Pterodactylus woodwardi*, *Ornithocheirus brachyrhinus*, *Ornithocheirus carteri*, *Ornithocheirus crassidens*, *Ornithocheirus dentatus*, *Ornithocheirus enchorhynchus*, *Ornithocheirus eurygnathus*, *Ornithocheirus oxyrhinus*, *Ornithocheirus scaphorhynchus*, *Ornithocheirus tenuirostris*, and *Ornithocheirus xyphorhynchus*.

Another major difference between the present work and the review done by Unwin (2001) concerns the identification of genera. Whereas Unwin (2001) placed the species of the *Ornithocheirus* complexin the genera *Ornithocheirus*, *Anhanguera*, *Coloborhynchus* and *Lonchodectes*, we here argue that *Anhanguera* and *Coloborhynchus* are restricted to the Romualdo Formation and the Hastings Group, respectively, and create two new genera for these forms, *Cimoliopterus* (with one species, *Cimoliopterus cuvieri*), and *Camposipterus* (with three species, two of them tentatively assigned). Although not formally erecting more genera, we suggest that some species (*‘Ornithocheirus’ capito*, *‘Pterodactylus’ daviesii*, *‘Ornithocheirus’ denticulatus*, *‘Ornithocheirus’ polyodon*, and *‘Ornithocheirus’ platystomus*) might represent additional genera, in line with Newton’s (1888) predictions.

The pterosaur assemblage from the Cretaceous of England possesses a high diversity. One reviewer pointed out that this may be an artifact caused by lack of knowledge of intraspecific variation. This may well be true, but, as previously noted (e.g., Unwin 2001), pterosaurs from this assemblage are too poorly known. Nevertheless, available material shows significant variation that hints at the occurrence of several species. Most of them come from the Cambridge Greensand, whose fossils were originally deposited in the Gault Clay Formation (Jukes–Browne 1875; Reed 1897; Barrett and Evans 2002) and were subsequently reworked into Cenomanian deposits.

The Cambridge Greensand is a remanié deposit (Unwin 2001), which implies time averaging. In view of this phenomenon, high pterosaur diversity is not surprisingly. Most probably, the comparatively high number of pterosaur species, as proposed here, has never coexisted in eastern England during the ‘middle’ Cretaceous, and the Cambridge Greensand pterosaur assemblage actually comprises a mixture of faunas.

Analysis of the phylogenetic relationships of these species, especially with the ones from the more or less coeval Romualdo and Crato formations of the Santana Group in Brazil, proved to be challenging, as expected. Using a modified version of the matrix by Wang et al. (2009), only a few characters could be scored, and missing data is a problem for this database. Several species were not recovered in a more inclusive group, including *Ornithocheirus simus* and *Lonchodraco giganteus*. Other species, however, had their phylogenetic positions retrieved with more confidence. *Cimoliopterus cuvieri* and *‘Ornithocheirus’ polyodon* are the sister groups of a newly recognized clade, Anhangueria. *Camposipterus*, *Ludodactylus*, *Brasileodactylus* and *‘Cearadactylus’ ligabuei* were found at the base of Anhangueria, and *Cearadactylus atrox* was confirmed as the sister group of Anhangueridae. Therefore, *‘Cearadactylus’ ligabuei*, described and tentatively referred to *Cearadactylus* by Dalla Vecchia (1993) belongs to a different genus, but further study of other crestless species from the Romualdo Formation is needed in order to determine if it is referable to *Brasileodactylus araripensis* (known only from a mandible) or another species. More importantly, the results of the phylogenetic analysis demonstrate that Ornithocheiridae *sensu* Unwin (2001) cannot be confirmed as a monophyletic entity, and should be restricted to its type species. Furthermore, Lonchodectidae (*sensu* Unwin 2001; here considered more or less equivalent to Lonchodraconidae) can be excluded from Azhdarchoidea.

## Supplementary Material

XML Treatment for Ornithocheiridae

XML Treatment for Ornithocheirus

XML Treatment for Ornithocheirus simus

XML Treatment for Lonchodraconidae

XML Treatment for Lonchodraco

XML Treatment for Lonchodraco giganteus

XML Treatment for Lonchodraco machaerorhynchus

XML Treatment for Lonchodraco(?) microdon

XML Treatment for Anhangueria

XML Treatment for Anhangueridae

XML Treatment for Coloborhynchus

XML Treatment for Coloborhynchus clavirostris

XML Treatment for ‘Ornithocheirus’ capito

XML Treatment for Camposipterus

XML Treatment for Camposipterus nasutus

XML Treatment for Camposipterus(?) sedgwickii

XML Treatment for Camposipterus(?) colorhinus

XML Treatment for Cimoliopterus

XML Treatment for Cimoliopterus cuvieri

XML Treatment for ‘Ornithocheirus’ polyodon

XML Treatment for ‘Ornithocheirus’ platystomus

XML Treatment for ‘Pterodactylus’ daviesii

XML Treatment for ‘Ornithocheirus’ denticulatus

XML Treatment for Palaeornis cliftii

XML Treatment for Osteornis diomedeus

XML Treatment for Pterodactylus compressirostris

XML Treatment for Pterodactylus fittoni

XML Treatment for Pterodactylus woodwardi

XML Treatment for Ornithocheirus brachyrhinus

XML Treatment for Ornithocheirus carteri

XML Treatment for Ornithocheirus crassidens

XML Treatment for Ornithocheirus dentatus

XML Treatment for Ornithocheirus enchorhynchus

XML Treatment for Ornithocheirus eurygnathus

XML Treatment for Ornithocheirus oxyrhinus

XML Treatment for Ornithocheirus scaphorhynchus

XML Treatment for Ornithocheirus tenuirostris

XML Treatment for Ornithocheirus xyphorhynchus

XML Treatment for Pterodactylus sagittirostris

## Appendix I

Character list (modified from Wang et al. 2009).

**1.** Dorsal margin of the skull:

0 – straight or curved downward; 1 – concave; 2 – only rostrum curved upward

**2.** Upper and lower jaw:

0 – laterally compressed; 1 – comparatively broad

**3.** Rostral part of the skull anterior to the external nares (modified):

0 – reduced; 1 – elongated

**4.** Rostral end of premaxillae/maxillae downturned:

0 – absent; 1 – present

**5.** Anterior tip of the rostrum with a dorsally reflected palatal surface (added from Andres and Ji 2008):

0 – absent; 1 – present

**6.** Position of the external naris:

0 – above the premaxillary tooth row; 1 – displaced posterior to the premaxillary tooth row

**7.** Process separating the external nares:

0 – broad; 1 – narrow

**8.** Dorsoventrally compressed and elongated naris:

0 – absent; 1 – present

**9.** Naris size relative to the antorbital fenestra:

0 – naris smaller than antorbital fenestra; 1 – naris larger than antorbital fenestra; 2 – both very reduced (slit–like)

**10.** Naris and antorbital fenestra:

0 – separated; 1 – confluent, shorter than 45% of the skull length; 2 – confluent, longer than 45% of the skull length

**11.** Antorbital fenestra, shape:

0 – eliptical or ovoid; 1 – triangular, with base and height subequal; 2 – triangular with height larger than base; 3 – very elongated anteroposteriorly

**12.** Orbit comparatively small and positioned very high in the skull:

0 – absent; 1 – present

**13.** Orbit pear–shaped:

0 – absent; 1 – present

**14.** Position of the orbit relative to the nasoantorbital fenestra (naris + antorbital fenestra):

0 – same level or higher; 1 – orbit lower than the dorsal rim of the nasoantorbital fenestra

**15.** Suborbital opening:

0 – absent; 1 – present

**16.** Premaxillary sagittal crest, position (modified):

0 – absent; 1 – confined to the anterior portion of the skull, beginning near or at the tip of the rostrum; 2 – starting anterior to the anterior margin of the nasoantorbital fenestra, not reaching the skull roof above the orbit; 3 – starting anterior to the anterior margin of the nasoantorbital fenestra, extending beyond occipital region; 4 – starting at about the anterior margin of the nasoantorbital fenestra, reaching the skull roof above the orbit but not extending over the occipital region; 5 – starting close or at the anterior portion of the skull and extended over the occipital region; 6 – starting close or at the anterior portion of the skull, reaching orbit but not extended over the occipital region; 7 – starting at the posterior half of the nasoantorbital fenestra; 8 – located at the anterior portion of the skull, beginning more posteriorly but before the anterior margin of the nasoantorbital fenestra.

**17.** Premaxillary sagittal crest shape (modified):

0 – striated, low with a nearly straight dorsal margin; 1 – striated, high with a nearly straight dorsal margin; 2 – striated, high, spike–like; 3 – round dorsal margin, bladeshaped; 4 – smooth, moderately expanded anteriorly and forming a low rod–like extension posteriorly; 5 – smooth, very expanded anteriorly and forming a low rod–like extension posteriorly; 6 – smooth, starting low anteriorly and very expanded posteriorly; 7 – smooth, with a straight anterior margin, forming a triangular lateral outline

**18.** Expansion of the premaxillary tip:

0 – absent; 1 – present, with jaw end high; 2 – present, with the jaw end dorsoventrally flattened

**19.** Posterior ventral expansion of the maxilla:

0 – absent; 1 – present

**20.** Maxilla–nasal contact:

0 – broad; 1 – narrow; 2 – absent

**21.** Nasal process:

0 – absent; 1 – placed laterally, long, straight, and directed ventrally (not fused with maxillae); 2 – placed laterally, reduced; 3 – placed medially, long; 4 – placed medially, reduced; 5 – placed laterally, short and directed anteriorly

**22.** Foramen on nasal process:

0 – absent; 1 – present

**23.** Anterior process of jugal rodlike and deflected dorsally

0 – absent; 1 – present

**24.** Lacrimal process of the jugal:

0 – broad; 1 – thin, subvertical; 2 – thin, strongly inclined posteriorly

**25.** Bony frontal crest:

0 – absent; 1 – low and blunt; 2 – low and elongated; 3 – high and expanded posteriorly

**26.** Bony parietal crest:

0 – absent; 1 – present, blunt; 2 – present, laterally compressed and posteriorly expanded, with a rounded posterior margin; 3 – present, constituting the base of the posterior portion of the cranial crest

**27.** Posterior region of the skull rounded with the squamosal displaced ventrally:

0 – absent; 1 – present

**28.** Position of the quadrate relative to the ventral margin of the skull:

0 – vertical or subvertical; 1 – inclined about 120° backwards; 2 – inclined about 150°backwards

**29.** Position of the articulation between skull and mandible:

0 – under the posterior half of the orbit or further backwards; 1 – under the middle part of the orbit; 2 – under the anterior half of the orbit

**30.** Helical jaw joint:

0 – absent; 1 – present

**31.** Supraoccipital:

0 – does not extend backwards; 1 – extends backwards

**32.** Foramen pneumaticum piercing the supraoccipital:

0 – absent; 1 – present

**33.** Expanded distal ends of the paroccipital processes:

0 – absent; 1 – present

**34.** Basisphenoid:

0 – short; 1 – elongated

**35.** Palate with ridge and mandible with sulcus (added from Bennett 1994; Andres and Ji 2008):

0 – absent; 1 – present

**36.** Palatal ridge (modified):

0 – discrete, tapering anteriorly; 1 – strong, tapering anteriorly; 2 – strong, confined to the posterior portion of the palate

**37.** Maxilla excluded from the internal naris:

0 – absent; 1 – present

**38.** Opening between pterygoids and basisphenoid (interpterygoid opening):

0 – absent or very reduced; 1 – present and larger than subtemporal fenestra; 2 – present but smaller than subtemporal fenestra

**39.** Large distinct foramina (cup–shaped structures) on the lateral side anterior portion of the dentary:

0 – absent; 1 – present

**40.** Mandibular symphysis:

0 – absent or very short; 1 – present, at least 30% of mandible length

**41.** Expansion of the dentary tip (new):

0 – absent; 1 – present, with jaw end high; 2 – present, with the jaw end dorsoventrally flattened

**42.** Anterior tip of the dentary downturned:

0 – absent; 1 – present

**43.** Tip of the dentary projected anteriorly:

0 – absent; 1 – present

**44.** Dentary bony sagittal crest, presence:

0 – absent; 1 – present

**45.** Dentary bony sagittal crest, shape:

0 –blade–like and short, placed anteriorly; 1 – massive and deep

**46.** Distinctively elongated and posteriorly oriented articular and retroarticular process:

0 – absent; 1 – present

**47.** Position and presence of teeth:

0 – teeth present, evenly distributed along the jaws; 1 – teeth absent from the anterior portion of the jaws; 2 – teeth confined to the anterior part of the jaws; 3 – jaws toothless

**48.** Teeth size distribution (modified):

0 – all teeth roughly the same size; 1 – larger teeth located at the tip of the rostrum; 2 – larger teeth located posteriorly

**49.** Variation in the size of the anterior teeth with the 5th and 6th smaller than the 4th and 7th:

0 – absent; 1 – present

**50.** Teeth with a broad and oval base:

0 – absent; 1 – present

**51.** Multicusped teeth:

0 – absent; 1 – present

**52.** Teeth finely serrated (modified):

0 – present; 1 – absent

**53.** Peg–like teeth:

0 – absent; 1 – present, 15 or less on each side of the upper jaws; 2 – present, more than 15 on each side of the upper jaws

**54.** Long slender teeth:

0 – absent or less than 150; 1 – present, more than 150

**55.** Laterally compressed and triangular teeth:

0 – absent; 1 – present

**56.** Alveoli located on an elevation at the anterior end of the upper and lower jaws (“parapet–like” alveoli) (new, based on Unwin 2001):

0 – absent; 1 – present

**57.** Notarium:

0 – absent; 1 – present

**58.** Atlas and axis:

0 – unfused; 1 – fused

**59.** Postexapophyses on cervical vertebrae:

0 – absent; 1 – present

**60.** Lateral pneumatic foramen on the centrum of the cervical vertebrae:

0 – absent; 1 – present

**61.** Midcervical vertebrae:

0 – short, sub–equal in length; 1 – elongated; 2 – extremely elongated

**62.** Cervical ribs on midcervical vertebrae:

0 – present; 1 – absent

**63.** Neural spines of the midcervical vertebrae:

0 – tall, blade–like; 1 – tall, spike–like; 2 – low, blade–like; 3 – extremely reduced or absent

**64.** Number of caudal vertebrae:

0 – more than 15; 1 – 15 or less

**65.** Caudal vertebrae with elongated zygapophyses forming rod–like bony processes:

0 – absent; 1 – present

**66.** Length of the scapula:

0 – subequal or longer than coracoid; 1 – scapula shorter than coracoid (1 > sca/cor > 0.80); 2 – substantially shorter than coracoid (sca/cor ≤ 0.80)

**67.** Proximal surface of scapula:

0 – elongated; 1 – sub–oval

**68.** Shape of scapula:

0 – elongated; 1 – stout, with constructed shaft

**69.** Coracoidal contact surface with sternum:

0 – no developed articulation surface; 1 – articulation surface flattened, lacking posterior expansion; 2 – articulation surface oval, with posterior expansion

**70.** Deep coracoidal flange:

0 – absent; 1 – present

**71.** Broad tubercle on ventroposterior margin of coracoid:

0 – absent; 1 – present

**72.** Cristospine:

0 – absent; 1 – shallow and elongated; 2 – deep and short

**73.** Proportional length of the humerus relative to the metacarpal IV (hu/mcIV):

0 – hu/mcIV > 2.50; 1 – 1.50 < hu/mcIV < 2.50; 2 – 0.40 < hu/mcIV < 1.50; 3 – hu/mcIV < 0.40

**74.** Proportional length of the humerus relative to the femur (hu/fe):

0 – hu/fe ≤ 0.80; 1 – 1.4 > hu/fe > 0.80; 2 – hu/fe > 1.40

**75.** Proportional length of the humerus plus ulna relative to the femur plus tibia (hu+ul/fe+ti):

0 – humerus plus ulna about 0.80% or less of femur plus tibia length (hu+ul/fe+ti ≤ 0.80); 1 – humerus plus ulna larger than 0.80% of femur plus tibia length (hu+ul/fe+ti > 0.80)

**76.** Pneumatic foramen on the ventral side of the proximal part of the humerus:

0 – absent; 1 – present

**77.** Pneumatic foramen present on dorsal side of the proximal part of the humerus:

0 – absent; 1 – present

**78.** Deltopectoral crest of the humerus:

0 – reduced, positioned close to the humerus shaft; 1 – enlarged, proximally placed, with almost straight proximal margin; 2 – subrectangular, extending down the humerus shaft for at least 30% of humerus length; 3 – distally expanded; 4 – enlarged, hatchet shaped, proximally placed; 5 – enlarged, hatched shaped, positioned further down the humerus shaft; 6 – enlarged, warped; 7 – long, proximally placed, curving ventrally

**79.** Medial (= ulnar) crest of the humerus:

0 – absent or reduced; 1 – present, directed posteriorly; 2 – present, massive, with a developed proximal ridge

**80.** Distal end of the humerus:

0 – oval or D–shaped; 1 – subtriangular

**81.** Proportional length of the ulna relative to the metacarpal IV (ul/mcIV):

0 – ulna 3.6 times longer than metacarpal IV (ul/mcIV > 3.6); 1 – length of ulna between 3.6 and two times the length of metacarpal IV (3.6 > ul/mcIV > 2); 2 – ulna less than two times the length of metacarpal IV (ul/mcIV < 2)

**82.** Diameter of radius and ulna:

0 – subequal; 1 – diameter of the radius about half that of the ulna; 2 – diameter of the radius less than half that of the ulna

**83.** Distal syncarpals:

0 – unfused; 1 – fused in a rectangular unit; 2 – fused in a triangular unit

**84.** Pteroid:

0 – absent; 1 – shorter than half the length of the ulna; 2 – longer that half the length of the ulna

**85.** Metacarpals I–III:

0 – articulating with carpus; 1 – metacarpal III articulates with carpus, metacarpals I and II reduced; 2 – not articulating with carpus

**86.** Proportional length of the first phalanx of manual digit IV relative to the metacarpal IV (ph1d4/mcIV):

0 – both small and reduced; 1 – both enlarged with ph1d4 over four times the length of mcIV (ph1d4/mcIV> 4.0); 2 – both enlarged with ph1d4 between two and four times the length of mcIV (4.0 > ph1d4/mcIV≥ 2.0); 3 – both enlarged with ph1d4 less than two times the length of mcIV (ph1d4/mcIV< 2.0)

**87.** Proportional length of the first phalanx of manual digit IV relative to the tibiotarsus (ph1d4/ti):

0 – ph1d4 reduced; 1 – ph1d4 elongated and less than twice the length of ti (ph1d4/ti smaller than 2.00); 2 – ph1d4 elongated about or longer than twice the length of ti (ph1d4/ti subequal/larger than 2.00)

**88.** Proportional length of the second phalanx of manual digit IV relative to the first phalanx of manual digit IV (ph2d4/ph1d4):

0 – both short or absent; 1 – elongated with second phalanx about the same size or longer than first (ph2d4/ph1d4 larger than 1.00); 2 – elongated with second phalanx up to 30% shorter than first (ph2d4/ph1d4 between 0.70 – 1.00); 3 – elongated with second phalanx more than 30% shorter than first (ph2d4/ph1d4 smaller than 0.70)

**89.** Proportional length of the third phalanx of manual digit IV relative to the first phalanx of manual digit IV (ph3d4/ph1d4):

0 – both short or absent; 1 – ph3d4 about the same length or larger than ph1d4; 2 – ph3d4 shorter than ph1d4

**90.** Proportional length of the third phalanx of manual digit IV relative to the second phalanx of manual digit IV (ph3d4/ph2d4):

0 – both short or absent; 1 – ph3d4 about the same size or longer than ph2d4; 2 – ph3d4 shorter than ph2d4

**91.** Proportional length of the femur relative to the metacarpal IV (fe/mcIV):

0 – femur about twice or longer than metacarpal IV (fe/ mcIV ≥ 2.00); 1 – femur longer but less than twice the length of metacarpal IV (1.00 < fe/mcIV < 2.00); 2 – femur about the same length or shorter than metacarpal IV (fe/mcIV ≤ 1.00)

**92.** Length of metatarsal III:

0 – more than 30% of tibia length; 1 – less than 30% of tibia length

**93.** Fifth pedal digit:

0 – with four phalanges; 1 – with 2 phalanges; 2 – with 1 or no phalanx (extremely reduced)

**94.** Last phalanx of pedal digit V:

0 – reduced or absent; 1 – elongated, straight; 2 – elongated, curved; 3 – elongated, very curved (boomerang shape)

## Appendix 2

Data matrix (modified from Wang et al. 2009).

Ornithosuchus longidens

0000000000 000000–000 0–00000000 00000–0000 0000–00000 0000000000 0000000000 0000000000 0000000000 0000

Scleromochlus taylori

000?00?000 00?000–000 0–0?00000? ????????0? 00?0–000?0 0?00?00??? 00?00????? ???00??0?? ?0?0?????? ????

Herrerasaurus ischigualastensis

0000000000 000000–000 0–00000000 00000–0000 0000–00000 0000000000 0000000000 0000000000 0000000000 0020

Anurognathus ammoni

010000100? ??0??0–00? 0–?00????? ????????00 0000–00000 0110000??? 0??10????? ??011??100 00?1011??? 001?

Jeholopterus ningchengensis

01000010?? ?????0–00? ????00??0? ????????0? 0000–00000 0110000??? 01?1000010 0?021??1?? 00?1011222 1011

Dendrorhynchoides curvidentatus

010000100? ?????0–00? 0–??0????? ????????0? 0000–00000 0110000??? 00?100?010 0?021??1?? 00?1011222 00??

Batrachognathus volans

?10000100? ?????0–00? 0–??0????? ??????0?00 0000–00000 011000??0? 0?0???001? ???21??10? ?????????? ????

Cacibupteryx caribensis

001??10000 000000–?00 0–00000000 00000–10?? ??????00?? ?????????? ?????????? ?????????? ?????????? ????

Eudimorphodon ranzii

0010010000 000000–000 0–0000010? 0?0?????00 0100–00000 1100000?00 00?0?00010 0?11?00200 11?10????? 1???

‘Eudimorphodon’ cromptonellus

?????????? ?????????? ????00???? ?000?????? ?????0???0 11??000??? ?????????? ??111????? 1???021211 101?

Carniadactylus rosenfeldi

?01??1??00 ????????0? ?????????? ?????????0 ??????0000 1100000?00 0????0???0 0?1110???? 1????21111 00??

Peteinosaurus zambellii

?0???????? ?????????? ?????????? ?????????? ?????000?0 1100000??? ???010001? ??111??100 11?1021111 0011

Dimorphodon macronyx

0010010010 200000–001 0–0000000? 0?0?????00 0000–00000 0100000?00 0000100010 ??11100100 1111021111 1011

Wukongopterus lii

0010010??? ???????00? ??0????1?? ????????00 0000–00000 0120000??0 10201000?0 ??111?01?? 10?1021111 1013

Dorygnathus banthensis

0010010010 000000–000 0–00000100 0?0????101 0010–00000 0100000??? 0000100010 ??11100300 10?1021111 1013

Scaphognathus crassirostris

0010010000 000000–000 0–00000100 0?000–010? 0000–00000 0100000?00 0000100010 0111100100 10?1021111 0013

Preondactylus buffarinii

00100100?0 ?0?000–00? 0–??000?0? ?????????0 0000–?0?00 ??00000??? 0??0?????? ??111??1?? 10?1021111 00??

Sordes pilosus

0010010000 ?00000–00? 0–?000010? 0?0?????00 0000–00000 01?0000?0? 0000100010 0?011??100 00?1021111 0013

Austriadactylus cristatus

0010010010 0000061000 0–000?000? ?????????? ??????0000 000000???? 00?00????0 ?????????? ?0???????1 ????

Angustinaripterus longicephalus

0010010100 3000020101 ??00????0? ????????00 ??????0000 01000????? ?????????? ?????????? ?????????? ????

Harpactognathus gentryii

00100101?0 ?????2?0?? ?????????? ????0–1??? ??????0??? ?????????? ?????????? ?????????? ?????????? ????

Campylognathoides zitteli

0010010010 100000–000 0–1000010? 0???????00 0100–00000 0100000000 0000100010 0?1???12?? 11?1012122 0010

Campylognathoides liasicus

0010010010 100000–000 0–10000100 0?000–1100 0100–00000 0100000000 0000100010 0111100200 11?1012122 1010

Caviramus schesaplanensis

?0???????? ?????????? ?????????? ????????10 ?000–100?0 110000???? ?????????? ?????????? ?????????? ????

Raeticodactylus filisurensis

0010010000 0000022000 0–00??010? ????????10 0001110000 1100000??? 00???????? ???21??20? ??????1211 ?0??

Rhamphorhynchus longicaudus

0010010020 000000–000 0–00000110 0?000–??01 0010–00000 010000000? 0000100010 0111100400 11?1022222 1012

Rhamphorhynchus muensteri

0010010020 000000–000 0–00000110 00000–1101 0010–00000 0100000000 0000100010 0111100400 11?1012222 1012

Pterodactylus antiquus

00100100–1 –00000–002 100000121? 0?0?0–??01 0000–00000 0120000000 1121000010 0121100700 21?1031222 2020

Pterodactylus kochi

00100100–1 –00000–002 100000121? 0?0?0–??01 0000–00000 0120000000 1121000010 0121100700 21?2031222 2020

Germanodactylus ramphastinus

00100100–1 –000040002 100000121? 0???????0? 0000–00000 0120000??? 11??0?001? ??211??7?0 21???31??? 2???

Germanodactylus cristatus

00100100–1 –000040002 100000121? 0?0?????01 0000–00000 0120000??? 11???00010 0?211??7?0 21???31222 21??

Gnathosaurus subulatus

00100100–1 –000020202 500000121? 0?0???0201 2000–00100 010000???? ?????????? ?????????? ?????????? ????

Pterodaustro guinazui

10100100–1 –00000–002 100000121? 0?0?????01 0000–00000 010100???0 11210?001? ??211??7?0 2?1??31222 2020

Ctenochasma gracile

10100100–1 –0?000–002 0–0?00121? 0?0?????01 0000–00000 010100??00 112?0?001? ??211??7?? 21???312?? 2020

Feilongus youngi

10100100–1 –000010002 10000212?0 0?0?0–??0? 0000–02000 010000???? ?????????? ?????????? ?????????? ????

Cycnorhamphus suevicus

10100100–1 –00000–002 200002121? ??0?0–??01 0000–02000 010000?00? 112??0001? ??210??700 21??031222 21??

Gallodactylus canjuersensis

10100100–1 –0?000–002 20??02??1? ??0?????0? 0?00–02000 010000???? ?????0001? ?????????? ??????1222 ?1??

Nyctosaurus bonneri

00100100–1 –00000–002 0–00000?1? 0?0?????01 0000–03–00 0100001?1? 010?0????? ??31???50? ????232222 2???

Nyctosaurus gracilis

00100100–1 –00000–002 0–00000?11 0?010–1201 0000–03–00 0100001110 0101000010 013111050? 2122232222 20??

Nemicolopterus crypticus

00100100–1 –00000–002 6001000?2? ????????01 0000–03–00 0100000??0 01?10000?0 ???11?17?? 2????31?22 2120

Aussiedraco molnari

?0???????? ?????????? ?????????? ????1???01 0000–?00?0 ???000???? ?????????? ?????????? ?????????? ????

‘Ornithocheirus’ polyodon

?010110??? ?????0–0?? ?????????? ????10???? ??????00?0 ???000???? ?????????? ?????????? ?????????? ????

‘Ornithocheirus’ denticulatus

?010110??? ?????0–0?? ?????????? ????10???? ??????0000 ???000???? ?????????? ?????????? ?????????? ????

‘Pterodactylus’ daviesii

?0???????? ?????????? ?????????? ????1???01 0000–?0000 ???000???? ?????????? ?????????? ?????????? ????

‘Ornithocheirus’ platystomus

?010?10??? ?????170?? ?????????? ????10???? ??????0??0 ???000???? ?????????? ?????????? ?????????? ????

Ornithocheirus simus

?010010??? ?????0–0?? ?????????? ????10???? ??????0000 ?100?0???? ?????????? ?????????? ?????????? ????

Cimoliopterus cuvieri

?010110??? ?????830?? ?????????? ????10???? ??????0000 01?000???? ?????????? ?????????? ?????????? ????

Pteranodon longiceps

10100100–1 –00000–002 4000330121 01010–1201 0000–03–00 0100001111 0111011010 0121110611 2122232222 2020

Nurhachius ignaciobritoi

00101100–2 –00000–002 ??02???12? ????????0? 0000–02000 01001010?? 011??1?1?0 ?22110?61? 222?1312?? 21??

Istiodactylus latidens

00101100–2 –?0??0–0?2 0–?2?????0 ????0–???1 0000–?2000 0100101?1? 0????11110 02?1???611 ?2?????1?? ????

Noripterus complicidens

?????????? ?????????? ?????????? ?????????? ?????????1 0?00?0??11 010??????? ??20001720 211??312?? 2120

‘Phobetor’ parvus

00100100–1 –100131012 0–0023012? 1?1?????01 0000–01–01 010000???? ?????????? ?????????? ??1??????? ????

Dsungaripterus weii

20100100–1 –100131012 0–00230121 1111101201 0000–01–01 0100001111 0101000010 ?2200??7?0 211?131222 2120

Lonchodraco(?) microdon

001??10??? ?????0–?0? ?????????? ????11???? ??????0000 ???001???? ?????????? ?????????? ?????????? ????

Lonchodraco machaerorhynchus

?0???????? ?????????? ?????????? ????1???01 ???11?00?0 ???001???? ?????????? ?????????? ?????????? ????

Lonchodraco giganteus

?010010??? ?????170?? ?????????? ????11??01 00010?0000 010001???? ?????????? ?????????? ?????????? ????

Zhejiangopterus linhaiensis

00100100–1 –00100–002 0–000?012? 1???????0? 0000–03–00 01000011?? 213?0000?1 0?200??7?? 21?2?313?? 2???

Azhdarcho lancicollis

?????????? ?????????? ?????????? ?????????? ??????3–00 0100001110 213??????? ?????107?0 ?????????? ????

*Quetzalcoatlus* sp.

00100100–1 –00107?002 0–00???121 ????0–1?01 0000–03–00 0100001110 213?000011 0?20010720 211??31322 2?20

Tupandactylus imperator

00110100–2 –011055002 ??0123012? 1????????1 ?001103–00 010000???? ?????????? ?????????? ?????????? ????

Tapejara wellnhoferi

00110100–2 –011055002 ??01230120 11110–1201 0001103–00 0100000?11 010?000010 1?20011720 2112?31??? 2120

Thalassodromeus sethi

00100100–2 –011056002 4001230121 1111121201 0000–03–00 010000???? ?????????? ?????????? ?????????? ????

Tupuxuara leonardii

00100100–2 –011056002 ??01230121 1111121201 0000–03–00 0100001111 010??00010 1220010720 211??313?? 2???

‘Cearadactylus’ ligabuei

10101100–? ?????0–102 ?????????? ????101??? ??????0100 010000???? ?????????? ?????????? ?????????? ????

Camposipterus(?) colorhinus

?01011???? ?????0–1?? ?????????? ????10???? ??????01?0 ???000???? ?????????? ?????????? ?????????? ????

Camposipterus(?) sedgwickii

?01011???? ?????0–1?? ?????????? ????10???? ??????0100 ???000???? ?????????? ?????????? ?????????? ????

Camposipterus nasutus

0010110??? ?????0–10? ?????????? ????10???? ??????0100 ???000???? ?????????? ?????????? ?????????? ????

Brasileodactylus araripensis

?0???????? ?????????? ?????????? ????1???01 1000–?01?0 0??000???? ?????????? ?????????? ?????????? ????

Ludodactylus sibbicki

10101100–1 –00000–?02 3100330121 ????101?01 ?000–00100 010000???? ?????????? ?????????? ?????????? ????

Cearadactylus atrox

00101100–1 –00001?102 ??00???121 ????101??1 1000–001?0 010000???? ?????????? ?????????? ?????????? ????

Caulkicephalus trimicrodon

?0101100–? –????13102 ????33???? ????101??? ??????0100 010000???? ?????????? ?????????? ?????????? ????

‘Ornithocheirus’ capito

?010110??? ?????1?1?? ?????????? ?????????? ??????01?0 010000???? ?????????? ?????????? ?????????? ????

Coloborhynchus clavirostris

?010110??? ?????1?1?? ?????????? ????10???? ??????01?0 ??00?0???? ?????????? ?????????? ?????????? ????

Liaoningopterus gui

?0101100–? –000013102 ??00??0121 ????????01 ?0010001?0 010000??11 01???????? ?????????? ?????????? ????

Anhanguera araripensis

00101100–1 –000013102 ??00110121 0101101201 ?????00110 0?0000???? ?????????? ?????01611 ?20??????? ????

Anhanguera spielbergi

00101100–1 –000013102 ?–00110121 0101101201 1001000110 0100001?11 ?????21120 02?1101611 ?2???????? ????

Anhanguera robustus

?0???????? ?????????? ?????????1 ????1???01 10010001?0 010000???? ?????????? ?????????? ?????????? ????

Anhanguera piscator

00101100–1 –000013102 3100110121 0101??1201 1001000110 0100000111 0111021120 0221101611 222?1????? 2120

Anhanguera blittersdorffi

00101100–1 –000013102 ??00110121 0101101201 1001000110 010000???? ?????????? ?????????? ?????????? ????

Anhanguera santanae

00101100–1 –00001?102 3100110121 0101101201 ?00??00110 0100000111 0111?21120 0????01611 ?22?1????? ????

Tropeognathus mesembrinus

00101100–1 –000013102 ??00110121 0101111201 1001000100 010000???? ?????????? ?????????? ?????????? ????
